# Computational design of environmental sensors for the potent opioid fentanyl

**DOI:** 10.7554/eLife.28909

**Published:** 2017-09-19

**Authors:** Matthew J Bick, Per J Greisen, Kevin J Morey, Mauricio S Antunes, David La, Banumathi Sankaran, Luc Reymond, Kai Johnsson, June I Medford, David Baker

**Affiliations:** 1 Department of BiochemistryUniversity of WashingtonSeattleUnited States; 2 Department of BiologyColorado State UniversityFort CollinsUnited States; 3 Molecular Biophysics and Integrated Bioimaging, Berkeley Center for Structural BiologyLawrence Berkeley National LaboratoryBerkeleyUnited States; 4 Ecole Polytechnique Fédérale de LausanneInstitute of Chemical Sciences and EngineeringLausanneSwitzerland; 5 Department of Chemical BiologyMax-Planck-Institute for Medical ResearchHeidelbergGermany; 6 Howard Hughes Medical InstituteUniversity of WashingtonSeattleUnited States; The Scripps Research InstituteUnited States

**Keywords:** protein design, biosensors, transgenic plants, *A. thaliana*, *E. coli*, *S. cerevisiae*

## Abstract

We describe the computational design of proteins that bind the potent analgesic fentanyl. Our approach employs a fast docking algorithm to find shape complementary ligand placement in protein scaffolds, followed by design of the surrounding residues to optimize binding affinity. Co-crystal structures of the highest affinity binder reveal a highly preorganized binding site, and an overall architecture and ligand placement in close agreement with the design model. We use the designs to generate plant sensors for fentanyl by coupling ligand binding to design stability. The method should be generally useful for detecting toxic hydrophobic compounds in the environment.

## eLife digest

Many small molecules, including toxins and some medicines, have flexible structures, which makes it difficult to detect and/or neutralize them. The pain medication fentanyl, for example, can rotate to adopt many shapes. In recent years, fentanyl drug abuse has become increasingly common, and the drug is often illegally produced. The number of deaths caused by fentanyl has risen greatly, which provides a strong reason to find new ways to detect this and other drugs.

Now, Baker et al. have created new sensors that are able to detect fentanyl. First, the 11 most likely shapes that fentanyl could adopt were identified based on known information about the structure of the molecule. Then, a computer program was used to design proteins that were predicted to strongly bind to these most common shapes. Next, genes that coded for these proteins were synthesized in the laboratory and introduced into bacteria, which read the genes to build the proteins.

Similar to a well-fitted lock and key, the shape of the newly designed protein had to complement a likely shape of the fentanyl molecule. Baker et al. used a technique called X-ray crystallography to visualize the proteins in atomic detail and confirm that these fentanyl-binders matched their corresponding computational models. Those proteins that bound fentanyl best were then engineered into plant cells, and later into whole plants, together with reporter systems that gave signals when the sensors detected fentanyl.

In future, these specifically synthesized proteins could be integrated into entire panels of plants or other systems to detect toxins and other harmful chemicals. Such systems would be of interest in a medical setting and for detecting environmental contamination.

## Introduction

Fentanyl is a potent agonist of the μ-opioid receptor (MOR), with an affinity of approximately 1 nM and a potency 100-times that of morphine (Volpe et al., 2011). It is used both pre- and post-operatively as a pain management agent. The fast acting nature and strength of fentanyl have been attributed to its high degree of lipophilicity (Peckham and Traynor, 2006). Fentanyl has become a widespread drug of abuse, and has played a central role in the growing opioid epidemic. Reports of illegal manufacturing and fentanyl-related deaths across the country and other parts of the world have increased significantly in recent years (Drug Enforcement Administration, 2017).

Custom-designed ligand-binding proteins offer the possibility of both detecting and counteracting toxins such as fentanyl. Antibodies raised against small molecules generally require mammalian expression systems and conjugation of the compound (hapten) to an immunogenic carrier protein. In addition, elicitation of antibodies by immunization does not provide control over the interactions that the protein makes with the ligand. In contrast, computationally designed proteins can be readily expressed in bacterial and other low-cost expression systems, and specific interactions can be directly programmed. However, computational design of precise protein–ligand interactions for flexible, predominantly hydrophobic compounds is challenging. As these molecules are in some sense ‘featureless’, due to their overall hydrophobic character, binding depends heavily on the protein-ligand shape complementarity. We previously reported a method for generating binders for relatively rigid molecules containing hydrogen bonding functional groups, where the focus was on solutions with optimal hydrogen bonding geometry (Tinberg et al., 2013). However, this approach is not well suited for flexible, nonpolar compounds such as fentanyl.

## Results

**Figure 1.**
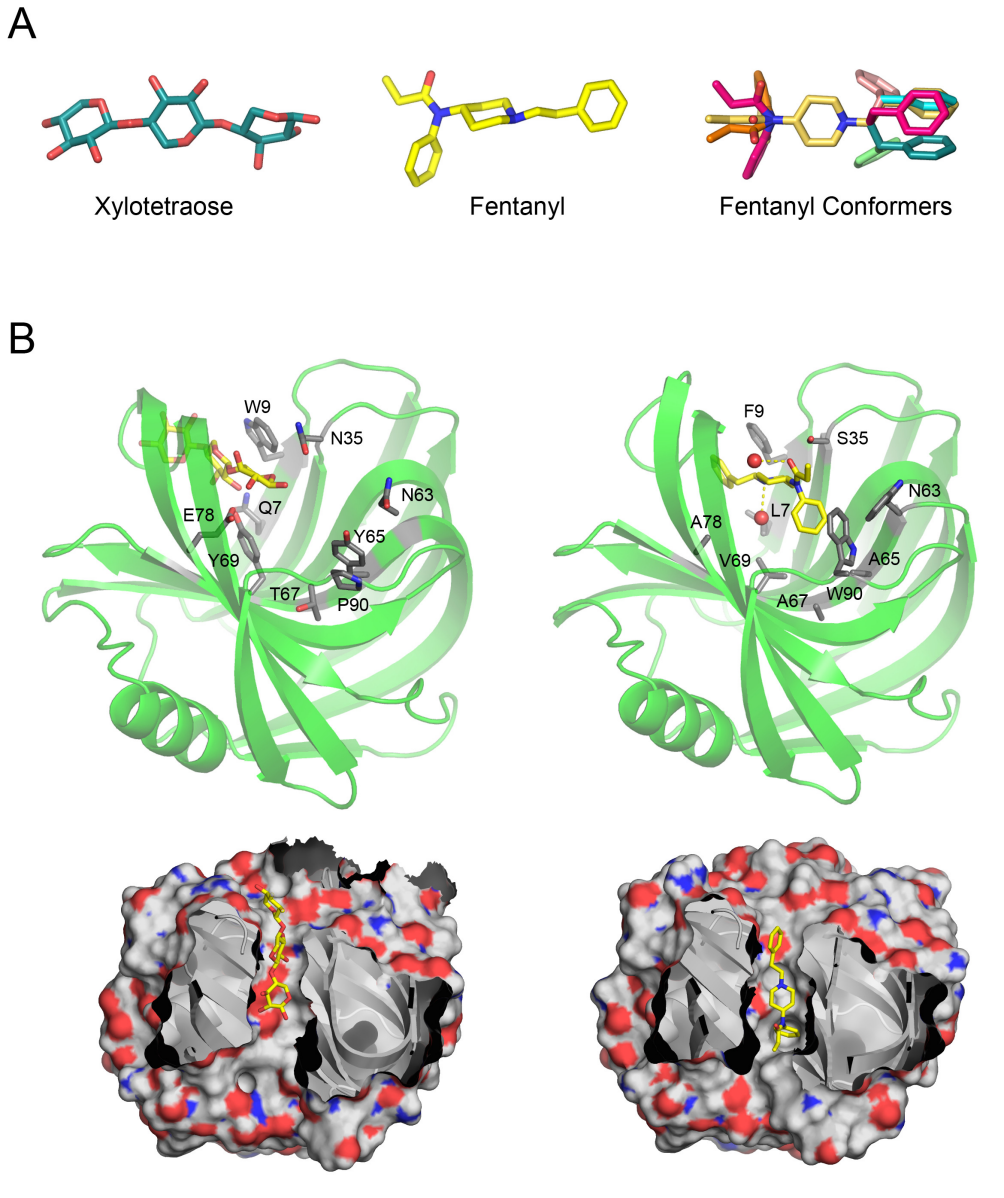
Overview of Fen49 and a comparison with the parent scaffold. (**a**) The sugar bound to the native scaffold (2QZ3), xylotetraose (left), is much more polar than fentanyl (middle), a predominantly hydrophobic compound. The right panel shows the 11 non-solvated fentanyl conformers used in design. (**b**) Cartoon representation of the 2QZ3 crystal structure (left) and the Fen49 computational model (right). Amino acid side chains colored grey represent the computationally introduced mutations in Fen49, and their native counterparts in 2QZ3. Designed fentanyl-associated waters are shown as red spheres, connected by yellow dashed lines. The corresponding surface representations of 2QZ3 and Fen49 (below) highlight the difference in charge distribution within the binding cavities and the shape complementarity between Fen49 and fentanyl.

We pursued a two-step approach to designing fentanyl binders (Figure 1—figure supplement 1). Fentanyl contains 6 rotatable bonds, which increases the combinatorial complexity of possible protein–ligand interactions to be considered. Starting from the structure of a fentanyl-citrate toluene solvate (Peeters et al., 1979), we generated 11 conformers plus an additional hydrated model of fentanyl, based on the small molecule structure, with non-covalently bound water atoms at both the tertiary amine (3 Å nitrogen to water distance, 109° carbon-nitrogen-water angle) and the carbonyl oxygen (3 Å oxygen to water distance, 120° carbon-oxygen-water angle) (Figure 1a). For each fentanyl conformer, we identified a large number of shape complementary placements of fentanyl within protein scaffolds from the MOAD database (Hu et al., 2005) using the fast docking algorithm PatchDock, which identifies shape complementary interactions between binding partners (Duhovny et al., 2002). Multipose binding has been observed in many naturally occurring protein-ligand complexes (Kulp et al., 2012; Blum et al., 2011; Barelier et al., 2015), but for our approach we sought precise control of the fentanyl pose by considering only a single conformer per protein scaffold.

**Figure 1—figure supplement 1.**
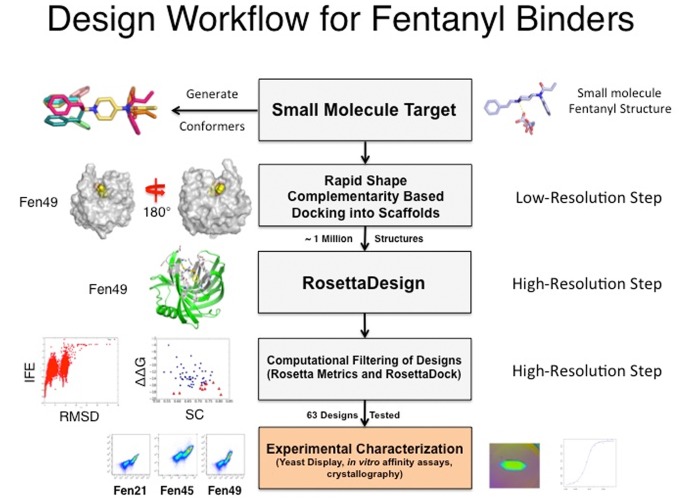
Computational and experimental flowchart.

In the second design step, we selected the top 20 scoring docks from PatchDock for each scaffold and optimized the identities and rotamer conformations of amino acids within 8 Å of fentanyl for shape complementarity and specific protein–ligand interactions. Similar to other MOR agonists, fentanyl possesses a charged tertiary amine, one of only two sites capable of making electrostatic interactions. We sought to exploit the tertiary amine to confer directionality and allow atomic level control over the placement of the otherwise hydrophobic molecule. Two design strategies were pursued: (1) The introduction of specific side chain–fentanyl interactions, either acidic (Asp or Glu) or cation-pi (Phe, Tyr, Trp) with the tertiary amine, and (2) the use of the hydrated fentanyl, as described above, for bridging indirect fentanyl-protein interactions. Designs were filtered based on shape complementarity, protein–fentanyl interface energy and the solvent-accessible-surface-area (SASA), and 62 were selected for experimental characterization.

**Figure 2.**
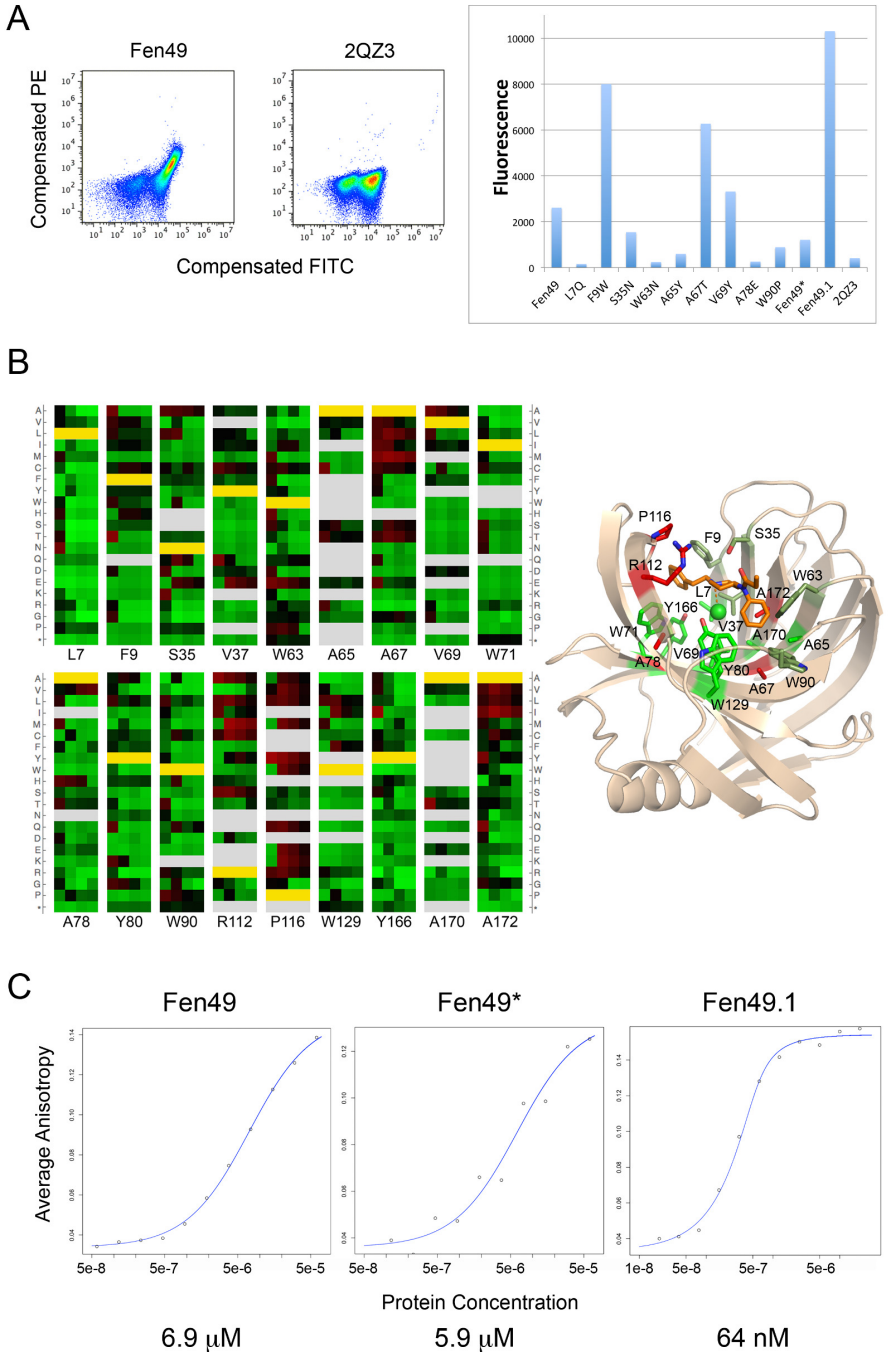
Experimental characterization of Fen49 binding and affinity enhancement. (**a**) left, Expression of Fen49 (FITC coupled anti-C-myc, x-axis) and binding of Fen-BSA (phycoerythrin (PE)-conjugated streptavidin, y-axis) on yeast. The Fen49 parent scaffold, 2QZ3, expresses well but does not bind Fen-BSA. (**a**) right, Mean PE fluorescence of individual Fen49 point mutants, Fen49* (Y88A), Fen49.1 (A78V A172I) and 2QZ3. (**b**) Enrichment map from 4 rounds of affinity maturation following site-saturation mutagenesis of the full Fen49 coding sequence. Binding site residues are highlighted (full SSM results are given in Figure 2—figure supplement 1). Green and red represent depletion and enrichment, respectively. Positions for which insufficient data were obtained in the naive library to make a comparison are colored grey. Fen49 design amino acid identities are colored yellow. Binding site side-chains are represented by sticks in the accompanying cartoon model of the Fen49*-complex structure, and colored according to enrichment (green = no enrichment away from the designed residue; red = strong enrichment away from the designed residue; olive = enrichment in early rounds of selection with depletion in later rounds. (**c**) Binding affinities (Kd) determined by equilibrium fluorescence anisotropy, using Fen-PEG-Alexa488 and Fen49 (left), Fen49* (middle) or Fen49.1 (right).

The designs were expressed using yeast surface display and probed for binding with a bovine serum albumin-fentanyl (Fen-BSA) conjugate. Sixty-one of the 62 designs expressed well, and three bound fentanyl with low micromolar to high nanomolar affinities. Fen49, the strongest binder (500 nM affinity for Fen-BSA) on yeast (Figure 2a), and Fen21 (10 μM) were chosen for further experimental characterization, as they represent two different scaffold classes. Of these two designs, recombinantly expressed Fen49 proved to be more stable and amenable to crystallization (see below).

**Figure 2—figure supplement 1.**
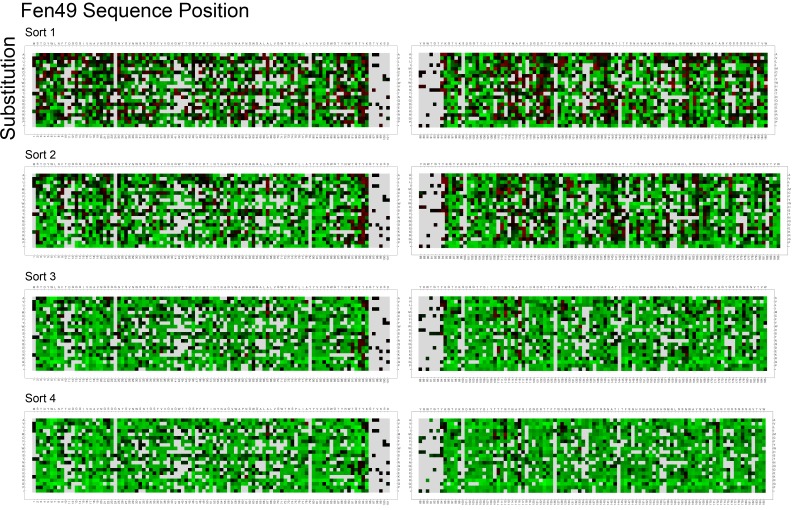
Enrichment results for the full Fen49 coding sequence after SSM and affinity maturation. A fentanyl-streptavidin tetramer (4-fold avidity) was used as the affinity reagent, with decreasing concentrations of 4 μM, 2 μM, 1 μM and 500 nM in subsequent rounds of selection. Enhancement and depletion are colored red and green, respectively. Positions with insufficient data in the naive library to make a comparison are colored grey.

**Figure 2—figure supplement 2.**
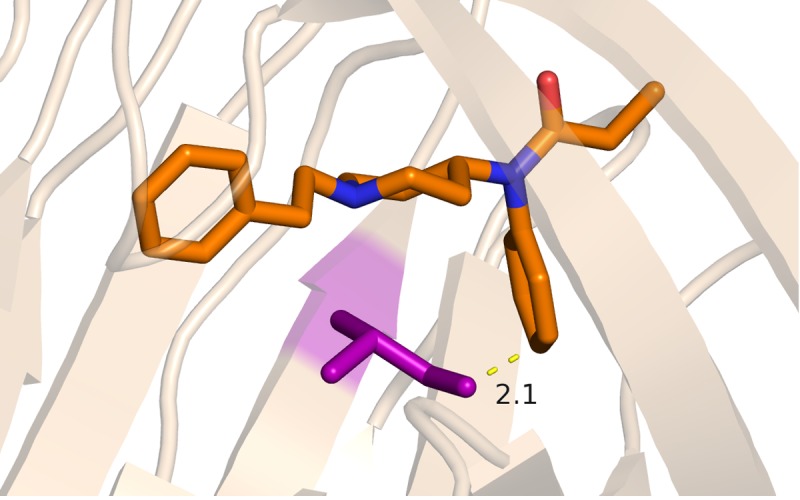
Fen49*-complex with isoleucine modeled at residue 172, an affinity-enriching mutation identified from SSM selection. Given the coordinates of fentanyl in the crystal structure, an isoleucine at position 172 would result in overlapping Van der Waals radii with fentanyl, suggesting that a slight shift in the position of fentanyl in Fen49.1 relative to Fen49* is likely. Shown is the typical C-C distance between I172 and fentanyl obtained from modeling different Ile rotamers. The fentanyl-associated chloride has been removed for clarity. Distance is in Å.

Following the placement of the hydrated fentanyl into the binding pocket via PatchDock, RosettaDesign introduced 9 mutations to the Fen49 scaffold to optimize the protein–ligand interactions (Figure 1b). Yeast–binding experiments of individual Fen49 point mutants corresponding to the computationally substituted positions showed that most are crucial for recognizing fentanyl (Figure 2b). Fentanyl does not bind the unmodified Fen49 scaffold (Figure 2a), a glycoside hydrolase (PDB 2QZ3). Purified Fen49 displayed an affinity of 6.9 μM for a fentanyl-Alexa-488 conjugate by fluorescence polarization (Figure 2c). We chose to conjugate the Alexa-488 fluorophore at the 4-phenyl position, as this site is compatible with the designed binding mode, and is also the site of fentanyl conjugation to the BSA probe used in our initial yeast display experiments (see Materials and Methods). 2QZ3 was cocrystallized with xylotetraose (only 3 of the 4 xylose molecules were placed in the final 2QZ3 model), a sugar molecule with a high degree of polarity compared with fentanyl (Figure 1b)(Vandermarliere et al., 2008). Such a dramatic repurposing of a sugar–binding protein is possible because the initial low-resolution docking step is agnostic to the polar character of the scaffold–binding cavity, as shape complementarity is the primary focus.

**Figure 3.**
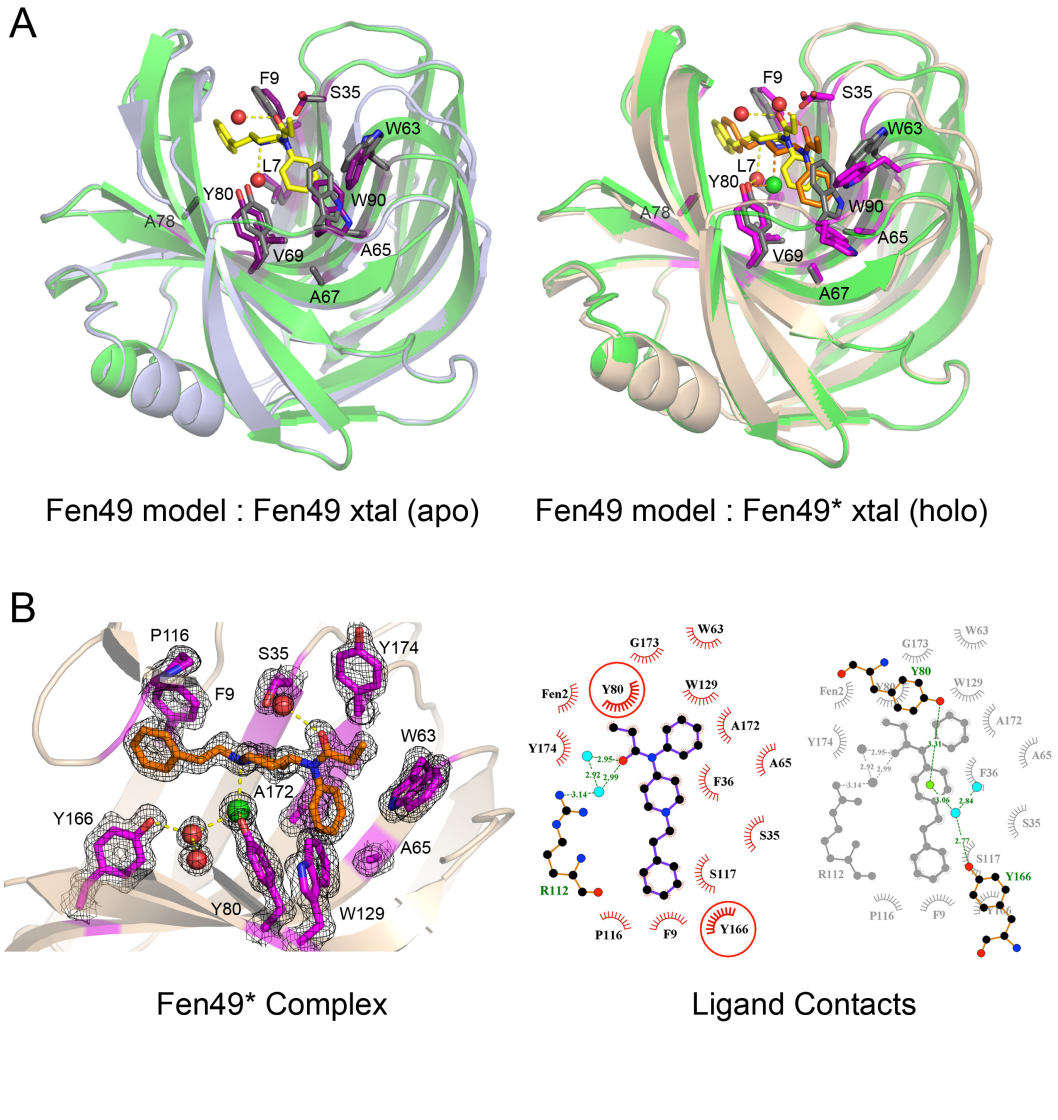
Structural characterization of Fen49. (**a**) left, Comparison of the Fen49 computational model (green, grey side-chains, yellow fentanyl) with the apo Fen49 crystal structure (light blue, purple side-chains). For clarity, the molecule of PEG observed in the crystal structure has been omitted. Computationally designed side-chains, plus Y80, are shown as sticks. Fentanyl-associated water molecules are shown as red spheres and connected with yellow dashed lines. (**a**) right, Fen49 model aligned with the Fen49*-fentanyl complex crystal structure (wheat, magenta side-chains, orange fentanyl). The fentanyl tertiary amine-associated chloride and carbonyl water are shown as green and red spheres, respectively. (**b**) left, The Fen49*-complex binding site, colored as in Figure 3a. Side-chains involved in binding fentanyl are shown, with the corresponding 2*F_o_-F_c_* electron density from the refined maps, contoured at 1.0 σ (Figure 3—figure supplement 8). (**b**) right, Ligplot (Laskowski and Swindells, 2011) 2D representation of all residues contacting fentanyl. Two superimposed layers are shown, keeping Y80 and Y166 fixed for orientation. Residues making hydrophobic and polar contacts are labeled with black and green font, respectively. Water molecules and the chloride ion are shown as blue circles and green circles, respectively. Green dashed lines represent hydrogen bonds (distances in Å). A second molecule of fentanyl was observed on the periphery of the binding cavity (Figure 3—figure supplement 9).

We solved an atomic resolution (1.00 Å) X-ray crystal structure of Fen49 in the apo state, one of the first examples of an original (non-optimized) computational design that has been structurally characterized (Figure 3a). The structure reveals a highly preorganized binding cavity (28 of 30 non-alanine/non-glycine side chains within ~8Å of fentanyl adopt the designed rotamer) and an overall structure in very close agreement with the design model; the r.m.s.d. of the design model to the parent structure is 0.26 over 184 of 185 residues (TM_align (Zhang and Skolnick, 2005) score of 0.99). The Fen49 apo crystals were obtained from a condition containing 25% polyethylene glycol (PEG) 3350 as the precipitant. During model building, a well-ordered portion of PEG was observed in the binding cavity (Figure 3—figure supplement 1). Soaking experiments with fentanyl tended to crack the crystals and destroy X-ray diffraction, likely as a result of PEG being displaced from the binding cavity. This, coupled with a lack of alternate crystal forms, prevented us from obtaining a structure of the parent Fen49-fentanyl complex.

**Figure 3—figure supplement 1.**
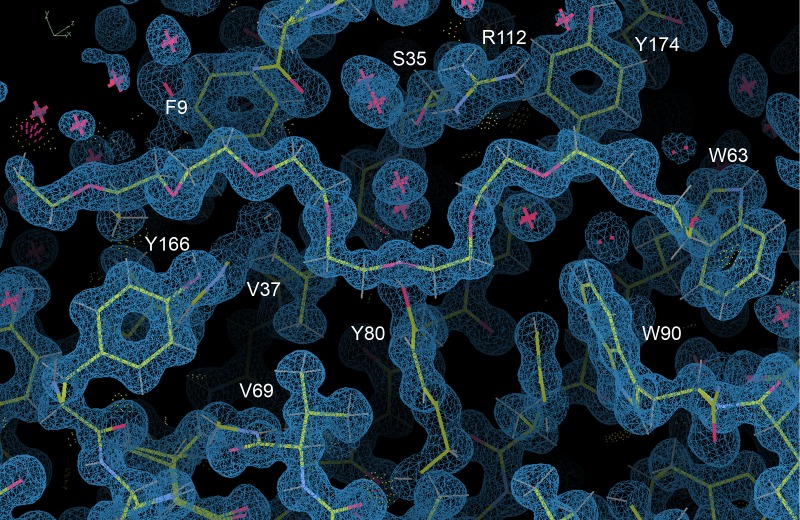
Fen49 binding site, showing a well-ordered portion of PEG 3350 from the crystallization solution. 2*F_o_-F_c_* map contoured at 1.0 σ. Image taken from Coot (Emsley et al., 2010).

**Figure 3—figure supplement 2.**
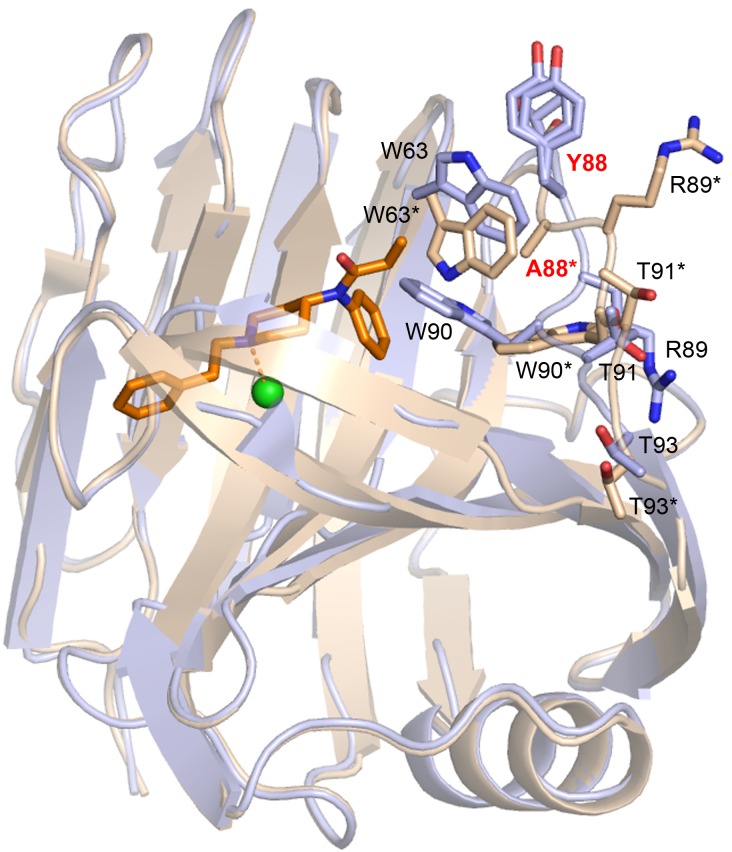
The Thr87 - Thr93 loop. containing the Y88A mutation displays a high degree of structural variability between Fen49 and Fen49*, which allows an exchange of the designed Trp90-fentanyl stacking interaction with a Trp63-fentanyl dipole-quadrupole. Fen49 (light blue) and Fen49*-complex (wheat) residues are labeled without and with an *, respectively.

**Figure 3—figure supplement 3.**
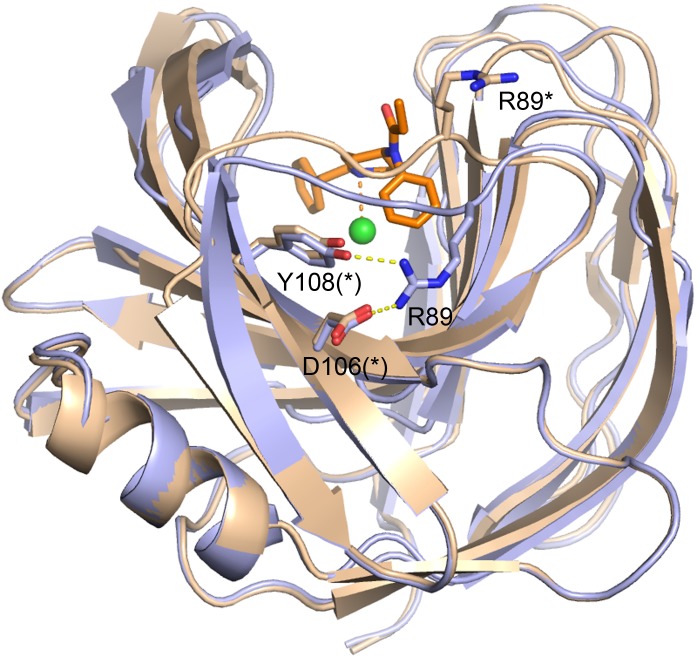
Disruption of the R89-D106-Y108 polar network on the back side of the binding cavity as a result of the altered Thr87 - Thr93 loop in Fen49* (wheat). Fen49 is colored light blue.

**Figure 3—figure supplement 4.**
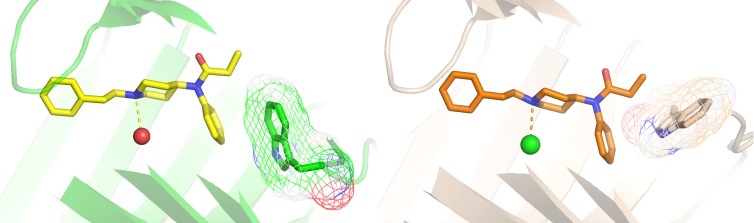
The W90-stacking interaction from the Fen49 design model (green) is replaced with a W63-fentanyl dipole-quadrupole (wheat).

**Figure 3—figure supplement 5.**
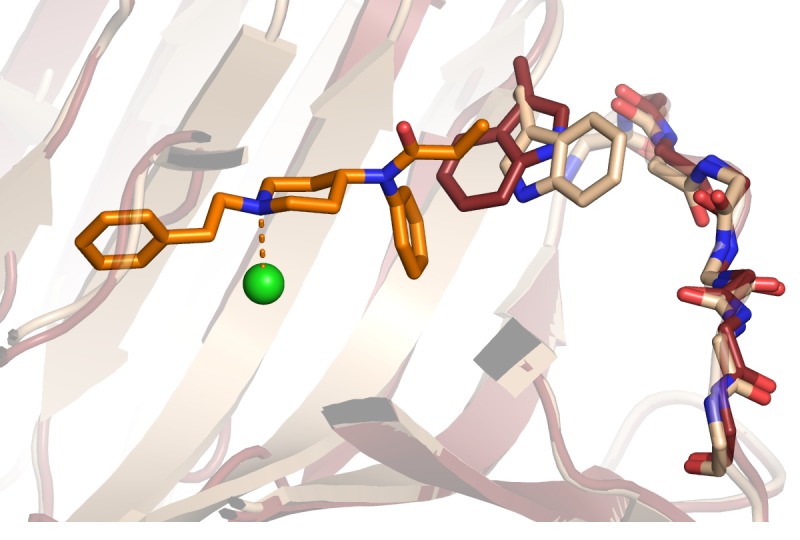
Difference between the Fen49*-apo (ruby) and Fen49*-complex (wheat) Trp63 side-chain rotamers. Also highlighted is the backbone of the Thr87 - Thr93 loop, which adopts the same conformation in both structures.

**Figure 3—figure supplement 6.**
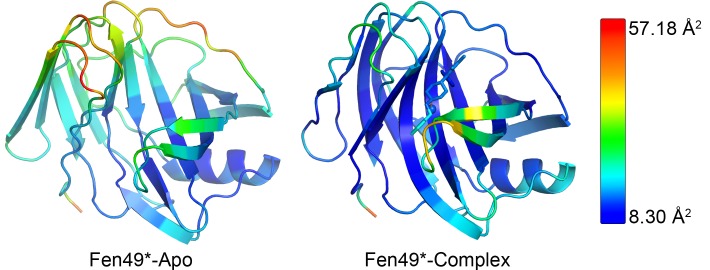
Chain A from the Fen49* crystal structures colored by B factor (Å^2^) of the C-alpha atoms, plus all fentanyl non-hydrogen atoms and the single molecule of chloride. B factors ranged from 10.86 to 57.18 (average B = 21.86) and 8.30–50.05 (average B = 15.89) for the apo and complex structures, respectively. Binding of fentanyl appears to stabilize the overall protein structure.

**Figure 3—figure supplement 7.**
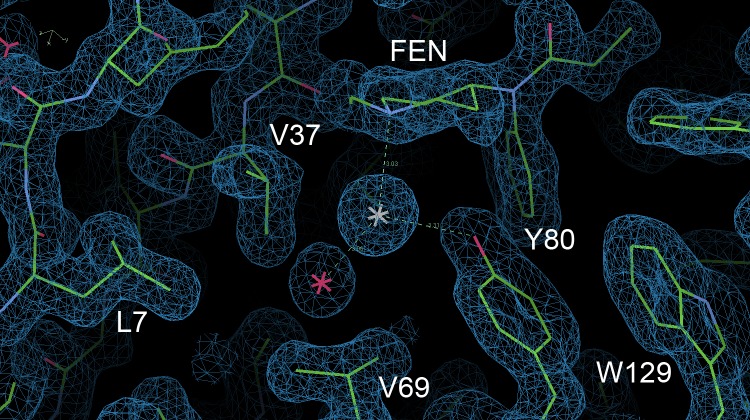
Trigonal planar coordination of chloride with fentanyl, Tyr80 and water in the binding cavity. 2*F_o_-F_c_* map contoured at 1.0 σ.

**Figure 3—figure supplement 8.**
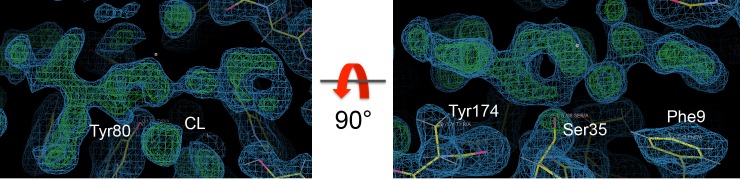
Positive density (*F_o_-F_c_* map colored green and contoured at 3.0 σ) corresponding to fentanyl and the associated chloride following molecular replacement and a single round of refinement with simulated annealing. Also shown is the 2*F_o_-F_c_* map colored blue and contoured at 1.0 σ.

**Figure 3—figure supplement 9.**
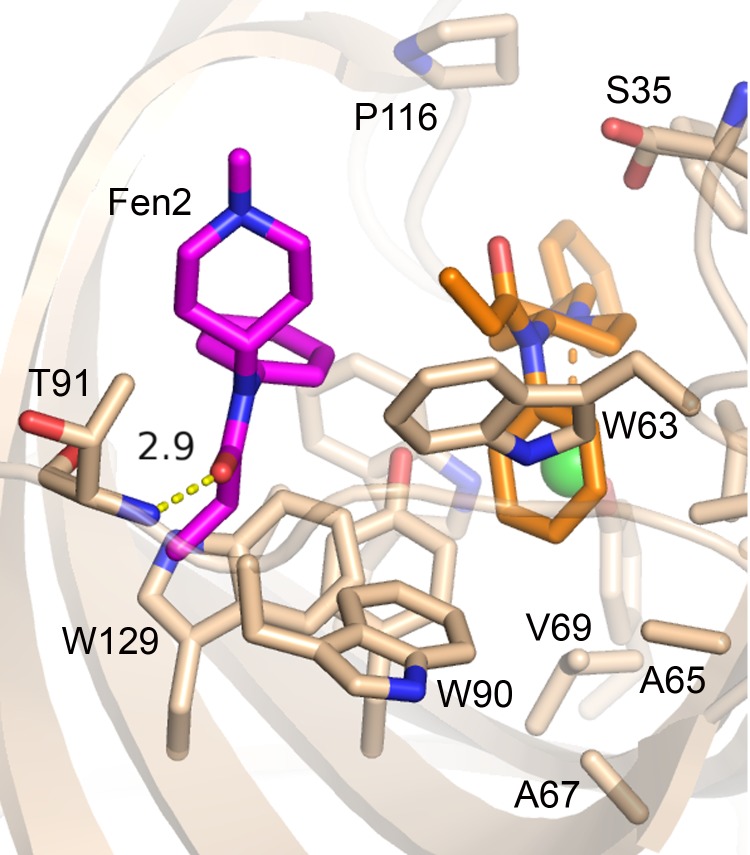
A second molecule of fentanyl was observed on the periphery of the Fen49 binding site. The phenylethyl moiety points towards the solvent and could not be modeled in 2 of the 3 copies in the asymmetric unit due to disorder. The second fentanyl makes a hydrogen bond to the backbone amide of Thr91 via its carbonyl oxygen.

To obtain a detailed map of the sequence determinants of folding and binding, we carried out site-saturation mutagenesis (SSM) on 184 of the 185 Fen49 residues, with the exception of the start methionine. At each position, each of the 20 amino acids were allowed, resulting in 3680 unique, single-mutant sequences (184 × 20 = 3680). Next-gen sequencing (millions of sequence reads) was carried out after each of 4 rounds of affinity enrichment (Figure 2—figure supplement 1). The majority of the binding site residues were preserved during selection, suggesting that Fen49 was designed with a near-optimal binding cavity (Figure 2b). Exceptions to this were three alanine residues, A67, A78 and A172, at the base of the binding pocket that were frequently substituted with larger hydrophobic residues, which provide additional packing for fentanyl. Two positions above the binding cavity enriched to amino acids that could reduce steric hindrance (Arg 112 to smaller aliphatic amino acids) or function as a hydrophobic lid over the binding site (Pro 116 to larger side chains). Charged amino acids, which might be expected to destabilize the hydrophobic cavity of Fen49, were disfavored during selection. However, a modest enrichment for glutamate at position 37 was observed in the second round of selection, suggesting an E37-tertiary amine salt bridge and the possibility of alternative poses of fentanyl within the binding site. This substitution was depleted in later rounds as a hydrophobic pocket was ultimately selected. We identified a combination of 2 substitutions, A78V plus A172I, that produced a Fen49 variant with an approximate 100-fold affinity improvement for fentanyl, to 64 nM (Figure 2c). These substitutions increase packing in the binding site, and likely require a modest positional adjustment of fentanyl to avoid a steric clash with I172 (Figure 2—figure supplement 2).

From the SSM experiments, we identified a Fen49 Y88A point mutant, termed Fen49*, that proved to be more suitable for complex structure determination (Figure 3 and Figure 3—figure supplement 2). The 1.79 Å Fen49*-apo structure again revealed a highly preorganized binding site, and an overall structure in close agreement with the Fen49 design (0.72 r.m.s.d. for Fen49* compared with the design model over 184 of 185 residues (TM_align score of 0.98)). The majority

of Fen49* side chains adopt the design conformations (25 of 30 non-alanine/non-glycine residues within ~ 8 Å of fentanyl are correct) and the structure shows minimal backbone rearrangements. The only significant deviation from the parent Fen49 is in the loop region Thr87 – Thr93, which contains the Y88A substitution (Figure 3 and Figure 3—figure supplement 2). In addition, a 3-residue polar network between Arg89, Asp106 and Tyr108 on the backside of the binding cavity is disrupted in Fen49* (Figure 3—figure supplement 3). As a consequence of the altered loop, tryptophans 63 and 90 adopt non-designed rotamers and collapse inwards towards the center of the binding cavity, with the designed Trp90-fentanyl stacking interaction replaced by a Trp63-fentanyl dipole-quadrupole (Figure 3b, Figure 3—figure supplement 2 and Figure 3—figure supplement 4).

Unlike the parent Fen49, Fen49*-apo produced crystals with an empty binding cavity that proved to be useful for soaking experiments. We solved a 1.67 Å Fen49*-fentanyl complex structure, which exhibits a high degree of similarity both with the designed model (r.m.s.d. of 0.64 over 184/185 residues, TM_align score of 0.99), and the Fen49*-apo structure (r.m.s.d. of 0.420 over all 185 residues, TM_align score of 0.99). The Thr87 – Thr93 loop adopts the same structure found in Fen49*-apo. With the exception of Trp63, which is flipped nearly 180° in the complex, fentanyl does not induce any significant changes to the active site upon binding (Figure 3—figure supplement 5). Fentanyl appears to stabilize the binding site; Fen49*-apo Trp63 and the Thr87 – Thr93 loop exhibit higher than average B-factors when compared both with the Fen49*-apo structure overall and with the corresponding residues in the Fen49* complex (Figure 3—figure supplement 6). Despite the divergent Thr87 – Thr93 loop, the parent Fen49 and Fen49* have virtually identical affinities for fentanyl, suggesting that this loop, and more specifically the differential Trp63-90 interaction with fentanyl, do not substantially lower the free energy of fentanyl binding. Instead, preorganization of the inner binding cavity residues appears to be the main determinant for binding.

Fen49 was designed to bind a solvated fentanyl. The water modeled at the fentanyl tertiary amine was introduced to bridge an indirect protein–ligand interaction with Tyr80. During structure refinement, a strong electron density peak was observed at this location (3 Å distance and 109.2° angle). Refinement with water at this position produced a strong positive signal in the *F_o_-F_c_* difference map, and it became clear that the density corresponded instead to a chloride ion (Figure 3). The chloride occupies the site of the designed water; it is coordinated by the tertiary amine, which is protonated at the crystallization pH (7.5), Tyr80 and a nearby water, a trigonal planar arrangement for chloride typically found in the PDB (Carugo, 2014) (Figure 3—figure supplement 7). To address the role of the chloride in binding, we carried out binding experiments using potassium phosphate (pH 7.4), free of chloride, as the assay buffer (protein was prepared in KPi as well). Nearly identical affinities were observed for Fen49.1, while Fen49* showed a modest 3-fold (~20 μM) reduction in affinity (data not shown), suggesting that a chloride may be preferred, but is not required for binding. We speculate that in the absence of chloride, a water molecule takes its place as in the design model. The Tyr80–chloride interaction observed in Fen49* is mimicked by a Tyr80-PEG hydrogen bond in the Fen49 parent structure (Figure 3—figure supplement 1). A second water molecule is observed bound to the fentanyl carbonyl oxygen at the designed position (2.7 Å distance, 135.2° angle).

**Figure 4.**
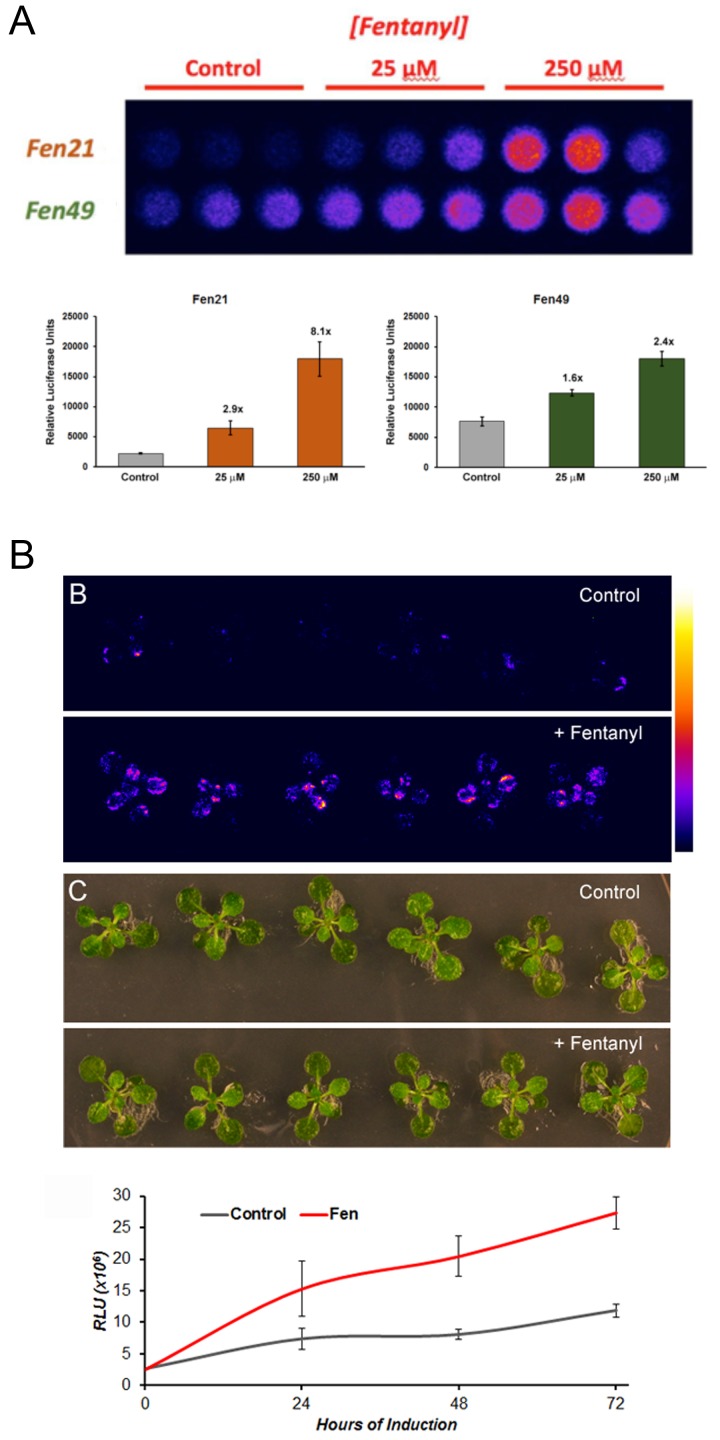
Fentanyl-dependent transcriptional activation in *Arabidopsis thaliana*. (**a**) Protoplasts expressing a conditionally stable transcription-factor (TF) firefly luciferase construct respond to treatment with fentanyl. Control cells did not receive fentanyl. Fen21 (~8 fold luciferase expression over background) was found to be more responsive to fentanyl compared with Fen49, and was used to generate intact transgenic plants shown in (**b**). Fen21 was refractory to crystallization, and therefore we were unable to obtain structural information for this design. (**b**) Fentanyl-dependent induction of luciferase activity in transgenic Arabidopsis plants expressing the Fen21 TF. Plants were incubated for up to 72 hr in liquid plant media containing no fentanyl (*Control*) or 100 µM fentanyl (*+Fentanyl*). Top panels, false-colored image depicting luciferase activity, according to the scale shown; middle panels, bright field photographs of plants; bottom panel, quantification of luciferase activity over 72 hr. Luciferase activity shown as Relative Luciferase Units (RLU).

A fentanyl detector would have applications in both medicine and public health. To this end, we incorporated our fentanyl binders into a transcription factor (TF)-based biosensor system, which couples ligand binding to transcription activation by stabilizing the protein against degradation (Banaszynski et al., 2006; Feng et al., 2015). This sensor can be placed in any system; we chose plants as they offer inexpensive on-site sensors for public health workers. We first tested our sensors in isolated plant cells (Arabidopsis protoplasts). The sensors were cloned into a plasmid containing a Gal4-responsive promoter-driving expression of a luciferase reporter, and expressed transiently in protoplasts. Fentanyl was added to the liquid media in which the protoplasts were incubated. In the presence of fentanyl, both the Fen21 and Fen49 TFs activated luciferase expression (Figure 4a). The Fen21-based sensor, which proved to be the better of the two, produced an 8-fold increase in luciferase expression over background when treated with 250 μM fentanyl. Fen49-expressing protoplasts displayed a modest background signal in the absence of ligand, suggesting that Fen49 is too stable in its apo form to function effectively as a sensor without additional engineering. To demonstrate that our computationally designed fentanyl sensor could function in a multicellular organism, we stably transformed Arabidopsis plants with the Fen21 TF directing luciferase expression. Plants were incubated in liquid plant culture media supplemented with 100 μM fentanyl (250 μM fentanyl was toxic to plants; data not shown). As early as 24 hr, the Fen21 TF transgenic plants showed an approximately 5-fold increase in luciferase activity, which increased to 10-fold at 72 hr post incubation (Figure 4b).

## Discussion

Neutralization of toxic compounds, either through binding or enzymatic breakdown, is an area of great interest for medical and environmental purposes. Our computational approach to designing environmental detectors lays the foundation for engineering practical plant-based sensors that are, in theory, able to detect and respond to any given small molecule. Unlike previous computationally designed ligand binders (Tinberg et al., 2013), the method that generated Fen49 did not involve any manual intervention, and hence could be rapidly applied to many ligands.

Fen49 does not possess any of the conserved sequence elements of MOR, a membrane bound GPCR, that are required for binding of agonists and antagonists (Surratt et al., 1994). Fentanyl is likely to make a direct salt bridge with MOR via its tertiary amine and a conserved aspartate in the third transmembrane helix of the receptor (Manglik et al., 2016; Manglik et al., 2012). In contrast, we have designed an entirely orthogonal, soluble protein that exploits indirect protein–ligand interactions and shape complementarity as the primary drivers of binding. With Fen49, we have expanded the repertoire of small molecules that are amenable to computational design to include predominantly hydrophobic, flexible ligands. Binders targeting toxic small molecules such as fentanyl should find useful applications as environmental sensors and antidotes.

## Materials and methods

### Generation of fentanyl conformers

Eleven conformers of fentanyl were generated based on an earlier investigation (Subramanian et al., 2000). To model solvation of the positive charge of the fentanyl tertiary amine and the polar carbonyl, explicit oxygen atoms were added at idealized distances and angles (3.0 Å N-O distance, 109° C-N-O angle for the tertiary amine; 3.0 Å O-O distance, 120° C-O-O angle for the carbonyl). These values were chosen based on the small molecule crystal structure of a fentanyl-citrate toluene solvate (Peeters et al., 1979).

### Scaffold selection

Scaffold proteins were comprised primarily of the 2010 MOAD set of high-resolution protein–ligand structures (Hu et al., 2005) (2454 PDBs), as well as a curated set of homologous proteins that had been shown to express well in the laboratory or that were suitable for computational design (399 PDBs). Also included was a set of 83 PDBs from the Pfam group of ketosteroid isomerases. The full list of PDB scaffolds used in this study is given in Supplementary Table 1 (Supplementary file 1).

### Generation of fentanyl parameters

To generate the fentanyl parameters for use in Rosetta (RRID:SCR_015701), an auxiliary script, which is distributed with the Rosetta software package, was used to convert the mol2/mol format to Rosetta parameters, using the command shown below. Execution of the command generates a parameter file in full atom as well as in centroid mode with a formal charge of +1 for fentanyl.Python ~/Rosetta/main/source/src/python/apps/public/molfile_to_params.py Fentanyl_cambridge.mol -n CFN -c --recharge=1

### Geometric placement of fentanyl in scaffolds using patchdock and matching

Scaffold proteins were used for initial docking of the set of fentanyl conformers. Docking was restricted to binding pockets identified by preexisting ligands in the crystal structure, or by RosettaHoles (Sheffler and Baker, 2009), which was used to define a position file of residues in the pocket. The 2.0 drug module of PatchDock (Schneidman-Duhovny et al., 2005) was used with the default settings for docking. The top 20 scoring poses for each scaffold were selected for subsequent RosettaDesign.~/Rosetta/main/source/bin/gen_apo_grids.linuxgccrelease -s PDBFILE -database ~/Rosetta/main/database @flags

Where the flags file contained the following (text following # are comments):-mute all
 -unmute apps.pilot.wendao.gen_apo_grids
 -chname off
 -constant_seed
 -ignore_unrecognized_res
 -packstat:surface_accessibility
 -packstat:cavity_burial_probe_radius 3.0 # if the cavity
 ball can be touched by probe r>3, then it is not in a pocket
 -packstat:cluster_min_volume 90 # minimum size of a pocket,
 smaller voids will not be considered
 -packstat:min_cluster_overlap 1.0 # cavity balls must
 overlap by this much to be clustered
 -packstat:min_cav_ball_radius 1.0 # radius of the smallest
 void-ball to consider
 -packstat:min_surface_accessibility 1.4 # voids-balls must
 be at least this exposed

These positions were set by using the receptorActiveSite in the PatchDock parameter file by initially converting the position file into PatchDock format:python splitfile.py PDBPOSITIONFILE.pos

Next, the parameters were generated using the supplied scripts: buildParams.plperl buildParams.pl PDBFILE FENTANYL_CONFORMATION 2.0 drug

The receptor active site was added to the parameter file:echo "receptorActiveSite POSITIONFILEPATCHDOCK" >> params.txt

Lastly, fentanyl was docked into each scaffold protein:patch_dock.Linux params.txt patchdock.out

For a small subset of the final 62 fentanyl binder designs, the RosettaMatch algorithm (Zanghellini et al., 2006) was used to introduce specific protein–fentanyl interactions to the subset of ketosteroid isomerase scaffold proteins (83 PDBs). Polar residues were used to introduce hydrogen bonds to the fentanyl carbonyl, while acidic (Asp and Glu) and aromatic residues (Phe, Trp and Tyr) were used to make charge–charge or dipole–quadrupole interactions, respectively, with the tertiary amine. A summary of the designs, their sequences and the method used to generate them is given in Supplementary Table 2 (Supplementary file 2).

### Rosetta design

For each docked or matched pose, residues within 8 Å of fentanyl were designed using the RosettaDesign (Leaver-Fay et al., 2011) algorithm to optimize the sequence around fentanyl for shape complementarity (SC) and protein–ligand interface energy. Initially, the designs were filtered based on the orientation of the ligand to allow egress of the chemical linker from the binding cavity for yeast display. For poses where the ligand had been placed according to the matching procedure, restraints were added and minimized in the context of an alanine backbone. For the initial round of designs, the catalytic residues were fixed and not allowed to change rotamer or amino acid identity. This was repeated 10 times and the lowest restraint score was kept for further design. The native scaffold residues were given a bonus of 1.5 REU, and the sequence was optimized with the matched residues fixed for 10 iterations. Again, the designs with the lowest interface energy (IFE) were kept. In order to limit the number of designs, we applied filters to remove poses that scored that did not meet the following thresholds: SC > 0.5, IFE < −10 REU, dsasa ≥ 0.8 of the ligand, a geometrical restraint score ≤ 5, packing of the rotamers in the binding pocket with an RMSD < 1 Å, ddG between the protein and the ligand of < −10 REU, and finally that the electrostatic field of the charged hydrogen and the oxygen of the carbonyl were negative. We furthermore made a greedy optimization of the residues in the interface such that they should contribute with at least an average energy to the interface energy, full-atom Dunbrack score, total score of the residue as well as its solvation score which we had computed from the CSAR 2010 high quality docking set using the enzdes score function. During the design process, we alternated between a linear version and an r6-r12 version of the Lennard-Jones potential.~/Rosetta/main/source/bin/rosetta_scripts.linuxgccrelease @flags -database ~/Rosetta/main/database -extra_res_fa FEN.FA.PARAMETERS @flags -parser:protocol match_enzdes_then_greedy.xml/refinement_empirical_w_cst.xml -in:file:s PDBFILE

For designs generated from Patchdock, no geometrical restraints were employed and the following flags were used for both runs:-run::preserve_header
 -enzdes::minimize_ligand_torsions 5.0
 -packing::use_input_sc
 -packing::extrachi_cutoff 1
 -packing::ex1
 -packing::ex2
 -linmem_ig 10
 -parser_read_cloud_pdb 1
 -ignore_unrecognized_res
 -enzdes::lig_packer_weight 1.5
 -hackelec_min_dis 1
 -hackelec_max_dis 2.5
 -no_optH false
 -enzdes
 -detect_design_interface
 -cstfile match1.cst
 -cst_opt
 -cst_min
 -flip_HNQ
 -no_his_his_pairE
 -hbond_params sp2_params
 -lj_hbond_hdis 1.75
 -lj_hbond_OH_donor_dis 2.6
 -correct
 -chemical:exclude_patches LowerDNA UpperDNA
 Cterm_amidation VirtualBB ShoveBB VirtualDNAPhosphate
 VirtualNTerm CTermConnect sc_orbitals
 pro_hydroxylated_case1 pro_hydroxylated_case2
 ser_phosphorylated thr_phosphorylated tyr_phosphorylated
 tyr_sulfated lys_dimethylated lys_monomethylated
 lys_trimethylated lys_acetylated glu_carboxylated
 cys_acetylated tyr_diiodinated N_acetylated
 C_methylamidated MethylatedProteinCterm
 -nblist_autoupdate
 -mute all


### Ligand docking with rosetta

In order to confirm that the designs possessed the minimum energy fentanyl poses, we performed RosettaDock and examined the energy and RMSD from the design model.~/Rosetta/main/source/bin/ligand_dock.linuxgccrelease @flags -database ~/Rosetta/main/database -extra_res_fa CFN.FA.PARAMS

The flags file contained the following information:-run
  -constant_seed
  -rng mt19937
 -in
  -file
   -s PDBFILE
  -native PDBFILE
  -extra_res_fa CFN.fa.params
 -out
  -nstruct 10000
  -file
 -packing
  -ex1
  -ex2
  -ex1aro
 -flip_HNQ
 -docking
  -randomize2
 -ligand
  -improve_orientation 10
 # Evaluate coulomb between protein and ligand
 -old_estat
 -ligand
  -improve_orientation 1000
  -minimize_ligand
  -harmonic_torsions 10
  -minimize_backbone
  -harmonic_Calphas 0.3
  -soft_rep


### General materials

Fen-BSA was purchased from CalBioreagents (10 mg/mLin dH_2_O,~20 fentanyl molecules per BSA, catalog # C149). Fentanyl monoclonal antibody was purchased from CalBioreagents (catalog # M571). Chicken anti-C-Myc-FITC conjugated polyclonal antibody was purchased from ICL Lab (catalog # CMYC-45F). Streptavidin-R-phycoerythrin conjugate was purchased from Invitrogen (catalog # S866). Fentanyl citrate was purchased from Sigma (catalog # F3886). *Arabidopsis thaliana*, ecotype Columbia Col-0 seeds were purchased from Lehle seeds (http://www.arabidopsis.com) and used to establish the protoplast and plant lines.

### Gene synthesis

Fen21, Fen49 and 2qz3 were purchased from Genscript with the coding sequence cloned into the NdeI and XhoI sites of vector pETCON, a modified version of pCTCON2 (Fleishman et al., 2011) that contains a c-terminal fusion to the c-myc epitope.

### Site-directed mutagenesis

Fen49 variants were generated by PCR using the megaprimer method (Ke and Madison, 1997) using the primers listed in Supplementary Table 3 (Supplementary file 3). Oligos were ordered from Integrated DNA Technologies, Inc.

### Biotinylation of Fen-BSA

Fen-BSA (10 mg/mL in dH_2_O) was first diluted to 2 mg/mL in PBS pH 7.4. Biotinylated Fen-BSA was prepared by reacting 14.3 μl of a 10 mM EZ-link-Sulfo-NHS-LC-Biotin solution (prepared in PBS pH 7.4, 10 eq) with 500 μl of Fen-BSA in an eppendorf tube shielded from light, on ice. After 4 hr the solution was dialyzed at 4°C against 500 mL of PBS in order to remove unreacted biotin reagent. The dialysis buffer was exchanged for an additional round. The extent of biotinylation was determined using a Pierce biotinylation quantitation kit. Reactions resulted in 1–3 molecules of biotin per BSA.

### Yeast surface display binding assays

All designs, derivatives and controls were transformed into *S. cerevisiae* EBY100 cells following the protocol outlined by Gietz and Schiestl, but without the single-stranded carrier DNA (Gietz and Schiestl, 2007). Transformants were plated on selective media (C *–ura –trp*) and incubated at 30°C for 48 hr. Colonies were picked and grown overnight in 1 mL of SDCAA (Chao et al., 2006) at 30°C, 225 RPM. The following day, 1e7 cells were harvested by centrifugation at 1000 x *g* for 2 min at RT in an Eppendorf microcentrifuge. The supernatant was removed, and the cells were resuspended in 1 mL of SGCAA induction media supplemented with 0.2% glucose. Protein expression was carried out at 18–22°C.

Following 36–48 hr of protein expression, 2e6 cells were collected into 96-well plates (Corning #3363). Cells were pelleted at 1000 x *g* for 2 min at 4°C and washed twice with 200 μl of ice-cold PBSF pH 7.4 (PBS supplemented with 1 g/L of BSA). Cells were resuspended in a 20 μl PBSF solution containing Fen-BSA at various concentrations. 1 μl of anti-fentanyl antibody (CalBioreagents) was added to positive control cells expressing two tandem Z domains of protein A (ZZ domain) (Mazor et al., 2007; Nilsson et al., 1987). Plates were incubated at 4°C for 4 hr on a Heidolph Tetramax 1000 plate shaker at 1350 RPM.

Unbound Fen-BSA was removed by centrifugation and washing the cells once with ice-cold PBSF. Cells were labeled with 0.5 μl of anti-C-myc-FITC conjugated antibody (1 mg/ml) and 0.5 μl streptavidin-phycoerythrin (SAPE, 3.3 μM) in a 20 μl volume of PBSF for 10 min at 4°C with shaking.

Cells were washed once with 200 μl of ice-cold PBSF to remove unbound C-myc and SAPE. Cell pellets were resuspended in 100 μl of ice-cold PBSF immediately prior to use. Protein expression and binding were measured on an Accuri C6 flow cytometer (488 nm excitation, 575 nm emission) by monitoring the FITC and PE fluorescence contributions, respectively.

### Fen49 Site-Saturation Mutagenesis (SSM) Library Generation and Selection

A single site-saturation mutagenesis (SSM) library was generated for the entire Fen49 coding sequence, with the exception of the start methionine, using a 2-step overlapping PCR method and pETCON-Fen49 as the template. The first step involved 2 separate PCR reactions to generate the 5’ and 3’ fragments flanking the site of interest. The reaction conditions were as follows: 16.125 μl ddH_2_O, 5 μl of HF Buffer (5X), 0.125 of Phusion High-Fidelity DNA Polymerase (NEB), 1.25 μl of 10 mM dNTPs, 0.5 μl of template DNA at 10 ng/μl, 1 μl each of either the 3’-MCS primer (5’-GTACGAGCTAAAAGTACAGTGGGAAC-3’) plus the forward NNK-containing primer, or the 5’-MCS primer (5’-TGACAACTATATGCGAGCAAATCCCCTCAC-3’) plus a reverse primer designed to have a partial overlap with the NNK primer. The second PCR step reconstituted the full Fen49 gene containing a single SSM site, plus 5’- and 3’-flanking sequences derived from the pETCON vector. The reactions conditions were as follows: 33.25 μl ddH_2_O, 10 μl HF Buffer (5X), 0.25 μl DNA Polymerase, 2.5 μl dNTPs, 1 μl each of the 5’- and 3’-MCS primers, 1 μl each of both PCR products described in step one. All primers were at 20 μM in dH_2_O. All amplifications were carried out by 30 cycles of PCR (98°C 15 s, 54°C 30 s, 72°C 60 s), with an initial 30 s melting step at 98°C and a final 5 min extension step at 72°C. The NNK degenerate primers used to generate the SSM library are listed in Supplementary Table 4 (Supplementary file 4).

All full-length Fen49 PCR products were pooled and purified by gel extraction (Qiagen) using ddH_2_O. Library DNA was used to electroporate *S. cerevisiae* EBY100 cells in triplicate, following a slightly modified version of the protocol detailed by Benatuil et al. (2010)., using 2 μg of NheI/XhoI/BamHI linearized pETCON and 6 μg of Fen49-SSM library DNA. Cells were electroporated using a Gene Pulser Xcell (Bio-Rad) at 2.5 Kv, 25 μF and 200 Ω. The library complexity was determined to be 5e8. Following the recovery step in YPD-sorbitol, cells were grown in 100 mL C -Trp -Ura media plus 100 μg/mL carbenicillin at 30°C for 24 hr, 225 RPM. Cells were pelleted at 1000 x *g* for 3 min, resuspended in 100 mL of fresh C -Trp -Ura and incubated for another 24 hr. The 3 100 mL library cultures were pooled, and 4e8 cells were pelleted and resuspended in 25 mL of SGCAA media supplemented with 0.2% glucose, 100 μg/mL carbenicillin and 50 μg/mL kanamycin. The library was expressed overnight at 22°C, 225 RPM. In order to identify Fen49 variants with a greater affinity for fentanyl than the parent design, a fentanyl-SAPE tetramer label was used in place of Fen-BSA (20 molecules of fentanyl per BSA) to reduce the dependency of the system on avidity. One-hundred-million cells from the naïve library were pelleted at 1000 x *g*, washed twice with 1 mL of ice-cold PBSF and labeled for 3 hr at 4°C, shielded from light, with 4 μM Fen-SAPE in a 1 mL volume of PBSF plus 20 μg/mL FITC conjugated anti-CMYC. Labeled cells were pelleted, washed once with 1 mL of ice-cold PBSF and resuspended in 4 mL PBSF. Cells with strongest signal in the PE channel (488 nm excitation, 585/30 nm optical filter) were collected with a SONY SH800 series cell sorter. Collected cells were grown in C *–trp –ura*.

### Next generation sequencing and site saturation mutagenesis analysis

Fen49 SMM library DNA was analyzed on an Illumina MiSeq, using the v3 kit (600 cycles), which produces 300-base reads. For full sequence coverage, the FEN49 library was split into 305 and 295 paired-end reads, respectively, where the overlapping sequence of the two portions was 42 base pairs. The next-generation sequencing of SSM libraries produced 5,017,520 forward and 5,093,932 reverse reads. The paired-end reads were assembled and filtered for quality (average *Phred* score ≥ 18 and a minimum position *Phred* score of ≥ 12). The paired-ends were further filtered based on a minimum of 11 overlapping reads. This resulted in 3,992,873 full length DNA reads (Supplementary file 5) that were translated to their corresponding protein sequence and mutation frequencies were determined using the Enrich software package (Fowler et al., 2011). The relative enrichment values, calculated as described below, were determined for mutations with >15 counts.

(1) $$p_{ij}^{select}=\frac{f_{ij}^{select}}{\sum_{i=1}^{N}\sum_{j=1}^{21}f_{ij}^{select}}$$

(2) $$p_{ij}^{naive}=\frac{f_{ij}^{naive}}{\sum_{i=1}^{N}\sum_{j=1}^{21}f_{ij}^{naive}}$$

(3) $$E_{ij}=log_{2}\frac{P_{ij}^{select}}{P_{ij}^{naive}}$$

The frequency of an observed mutantion at position *i* for mutation *j* in the selected ($f_{ij}^{select}$) and naive ($f_{ij}^{naive}$) libraries were determined from the full length reads. Mutation frequencies over all positions and mutation types were summed to determine the total frequency of mutants in a selected and naive library for the full protein sequence length (*N*). The possible mutations include the 19 standard amino acids (wild-type amino acid identities were not considered) and a sequence termination encoded by stop codon. The probability of a mutation in a sequence for the selected ($p_{ij}^{select}$) and naive library ($p_{ij}^{naive}$) were calculated as shown in Equation 1 and Equation 2, respectively. The enrichment ($E_{ij}$) value was determined as shown in Equation 3. SSM heat maps were generated using the MatrixPlot function in Mathematica.

### Synthesis of biotinylated and Alexa488 fentanyl derivatives

All chemical reagents and anhydrous solvents for synthesis were purchased from commercial suppliers (Sigma-Aldrich, Fluka, Acros) and were used without further purification or distillation. The composition of mixed solvents is given by the volume ratio (v/v). ^1^H and ^13^C nuclear magnetic resonance (NMR) spectra were recorded on a Bruker DPX 400 (400 MHz for ^1^H, 100 MHz for ^13^C, respectively), Bruker AVANCE III 400 Nanobay (400 MHz for ^1^H, 100 MHz for ^13^C, respectively), with chemical shifts (δ) reported in ppm relative to the solvent residual signals of CDCl_3_ (7.26 ppm for ^1^H, 77.16 ppm for ^13^C), CD_3_OD (3.31 ppm for ^1^H, 49.00 ppm for ^13^C), DMSO-*d*_6_ (2.50 ppm for ^1^H, 39.52 ppm for ^13^C). Coupling constants are reported in Hz. High-resolution mass spectra (HRMS) were measured on a Micromass Q-TOF Ultima spectrometer with electrospray ionization (ESI) or Bruker MicroTOF with ESI-TOF (time-of-flight). LC-MS was performed on a Shimadzu MS2020 connected to a Nexerra UHPLC system equipped with a Waters ACQUITY UPLC BEH C18 1.7 µm 2.1 × 50 mm column. Buffer A: 0.05% HCOOH in H_2_O Buffer B: 0.05% HCOOH in acetonitrile. Analytical gradient was from 5% to 95% B within 5.5 min with 0.5 ml/min flow. Preparative RP-HPLC was performed on a Dionex system equipped with an UVD 170U UV-Vis detector for product visualization on a Waters SunFire Prep C18 OBD 5 µm 10 × 150 mm Column (Buffer A: 0.1% TFA in H_2_O Buffer B: acetonitrile. Typical gradient was from 0% to 90% B within 30 min with 4 ml/min flow.). After lyophilization of HPLC purified compounds, the solid residue was generally dissolved in dry DMSO.

### Alexa488-PEG-NH_2_synthesis (Compound 1)

Alexa 488 carboxylic acid (Thermofisher scientific, 5 mg, 6.0 μmol) was dissolved in dry DMSO (250 μl), treated with diisopropylethylamine (5 μl, 23.1 μmol) and TSTU (2.8 mg, 9.3 μmol). The mixture was incubated for 5 min at r.t. and 1,8-diamino-3,6-dioxaoctane (5.7 mg, 38.5 μmol) was added. After 20 min, the reaction was purified by RP-HPLC and lyophilized, yielding an orange-red solid (2.3 mg, 58% yield). HRMS (ESI) calcd for C_27_H_29_N_4_O_12_S_2_ [M+] 665.1218; found 665.1239.

### Fentanyl-PEG-Alexa488 synthesis

A solution of compound 1 in DMSO (7.0 mM, 250 μl, 1.7 μmol) was mixed with fentanyl isothiocyanate (Tocris Bioscience) in solution in DMSO (40 mM, 50 μl, 2.0 μmol). Diisopropylethylamine (13 μl, 75 μmol) was added and the solution was incubated for 2 hr at r.t. The product was purified by RP-HPLC and lyophilized, yielding an orange-red solid (1.0 mg, 59% yield). HRMS (ESI) calcd for C_50_H_56_N_7_O_13_S_3_ [M^+^] 1058.3098; found 1058.3093.

**Chemical structure 1.**
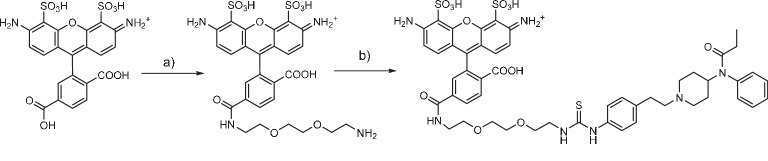
Fentanyl-PEG-Alexa488 synthesis (a) TSTU, 1,8-diamino-3,6-dioxaoctane, DIPEA, DMSO, r.t. (b) fentanyl isothiocyanate, DIPEA, DMSO, r.t.

### Fentanyl-PEG-biotin synthesis

Biotin-(PEO)4-amine (SCBT) (17.0 mg, 40.6 μmol) and fentanyl isothiocyanate (Tocris Bioscience) (5 mg, 12.7 μmol) were dissolved in DMSO (200 μl) and incubated for 1 hr at r.t. The product was purified by RP-HPLC and lyophilized, yielding a white solid (2.6 mg, 25% yield). HRMS (ESI) calcd for C_41_H_62_N_7_O_6_S_2_^+^ [M + H]^+^ 812.4197; found 812.4191.

**Chemical structure 2.**
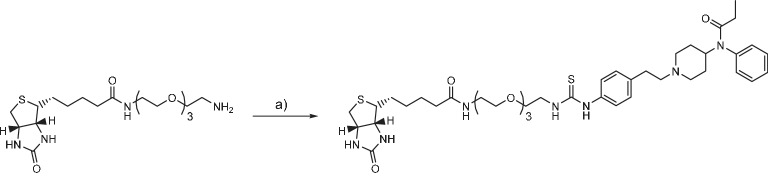
Fentanyl-PEG-biotin synthesis (a) fentanyl isothiocyanate, DIPEA, DMSO, r.t.

### Bacterial protein expression and purification

Expression and purification methods refer to Fen49 and all derivatives. Coding sequences were subcloned from their pETCON constructs into the NdeI and BamHI sites of a modified version of pET28a (Novagen), which replaces the N-terminal thrombin cleavage site with a PreScission Protease (GE Healthcare Life Sciences) site. Expression clones were transformed into BL21 (DE3) cells and grown overnight in 2 mL of Terrific Broth II (TB-II, MP Biomedicals) supplemented with 150 μg/mL carbenicillin without first plating for colony selection. Overnight cultures were used to inoculate 1 L of TB-II and subsequently grown at 37°C until an OD₆₀₀ of 0.8–1.0, at which point the shaker temperature was dropped to 18°C and protein expression carried out for 16–20 hr by the addition of IPTG to a final concentration of 0.1 mM.

Cells were harvested by centrifugation at 4°C, 7500 x *g* for 20 min and the pellets from 2 L of culture were resuspended in ~30 mL of Nickel Buffer A (500 mM NaCl, 20 mM Tris pH 8.0, 30 mM imidazole and 5% glycerol) and stored at −80°C. Purifications were performed from 6 L of cells. Cells were lysed, while in an ice-water bath, by sonication using a Sonic Dismembrator Model 505 (Fisher Scientific) at 70% amplitude (4 × 1 min cycles of 5 s pulses followed by 10 s rest, with 1 min in an ice-water bath in between cycles). Lysates were clarified by centrifugation at 4°C, 43,000 x *g* for 30 min.

The supernatant was loaded onto a 5 mL HisTrap FF column, charged with NiSO_4_, at 2.5 mL/min using an ÄKTA Pure fast-protein-liquid-chromatography (FPLC) system (GE Healthcare Life Sciences). The column was washed with Nickel Buffer A until a baseline absorbance was achieved. Fen49 was eluted from the column by performing a linear gradient to 100% Nickel Buffer B (500 mM NaCl, 20 mM Tris pH 8.0, 200 mM imidazole and 5% glycerol) over 25 mL, and 2 mL fractions were collected.

Fractions containing Fen49 were pooled, PreScission Protease was added at a ratio of 1:20 with Fen49, and the protein was dialyzed at 4°C against 2 × 1L of 50 mM NaCl, 20 mM Tris pH 8.0, 5% glycerol. Cleavage with PreScission Protease leaves a vector derived gly-pro-his sequence on the N-terminus of the Fen49 sequence. Cleaved Fen49 was passed over a 5 mL GST HiTrap column and Q HiTrap column in series at 1 ml/min. The flow through containing Fen49, but free from contaminating proteins, was collected. Fen49 purity was estimated to be > 95%. All FPLC steps were carried out at RT.

Fen49 was concentrated at 4000 x *g* using an Amicon Ultra-15 10K Centrifugal Filter (EMD Millipore) to ~200 μl. Protein buffer was exchanged to 10 mM NaCl 5 times by dilution to 15 mL and re-concentration to 200 μl.

### Fluorescence anisotropy equilibrium saturation binding assays

Fluorescence polarization experiments were performed as previously described (Rossi and Taylor, 2011). All experiments were conducted at 25°C in a SpectraMax M5e microplate reader (Molecular Devices) at excitation and emission wavelengths of 485 nm and 538 nm, respectively, using a 515 nm emission cutoff filter. Experiments were carried out in 40 μl reaction volumes using High efficiency 96-well black opaque microplates (Molecular Devices). Fentanyl-PEG-Alexa488 (Fen-A488) was used as the fluorescent ligand in all experiments. All protein and ligand dilutions were made in PBS, pH 7.4. For all experiments, the concentration of Fen-A488 was held at 500 nM, while the protein concentration was varied from 60 μM to 20 nM. Anisotropy values were collected over a period of 15 min, and the equilibrium dissociation constants (*K*_d_) were determined as previously described (Tinberg et al., 2013).

### Protein crystallization

Protein was spun at 4°C, 20,817 x *g* for 20 min to remove insoluble material prior to crystallization. Typically none was observed. Protein concentration was determined using the Bradford Protein Assay (Bio-rad) and BSA to generate the standard curve. Crystallization trials were conducted using a variety of 96-condition spare matrix suites from Qiagen and Hampton Research. All crystallization trials were conducted with the sitting drop vapor diffusion method at 20°C, using 3-well MRC crystallization plates (Swissci).

Fen49 crystallization trials were conducted using a Mosquito Crystal nanoliter robot (TTP Labtech). Fen49 at 30 mg/mL was mixed in 1:2, 1:1 and 2:1 protein to crystallization solution ratios in 400 nl drops. Crystals displaying a shard-like cluster morphology were obtained after ~1 week from a solution containing 0.1M citric acid pH 3.5% and 25% (w/v) PEG-3350. Microseeding was employed in order to obtain crystals suitable for diffraction experiments. A drop containing Fen49 crystals was added to 50 μL of crystallization solution in a microfuge tube containing a Seed Bead (Hampton). This solution was vortexed for 30 s. Fresh drops were set up using 1 μL of Fen49 at 20 mg/mL plus 1 μL crystallization solution, to which 0.2 μL of a 1:100 dilution of the seed stock was added. Large, single crystals were observed overnight, which grew to a maximum size of over 300 μm in length within ~3 days. Crystals were briefly dipped in a solution of 0.085M citric acid pH 3.5, 21.25% (w/v) PEG-3350% and 15% glycerol and flash frozen in liquid nitrogen.

Crystals of Fen49*-apo were obtained manually by mixing 0.5 μL of protein at 10 mg/mL with 0.5 μL and 0.8 M sodium phosphate, 0.8 M potassium phosphate and 0.1 M HEPES pH 7.5. Rod-like crystals appeared after ~3 days and grew to a maximum of 200 μm in length. Crystals were briefly soaked in a solution of 0.6M sodium phosphate, 0.6 M potassium phosphate, 0.075 M HEPES pH 7.5% and 25% glycerol, then flash frozen in liquid nitrogen.

The Fen49*-fentanyl complex was obtained by soaking Fen49*-apo crystals overnight in mother liquor plus 20 mM fentanyl citrate (solution made in dH_2_O to ~50 mM). The soaked crystals were sealed in a well containing mother liquor to allow excess water from the added fen-citrate to diffuse. Fen49*-complex crystals were cryo-protected the same as Fen49*-apo. Crystals were flash frozen the same as for Fen49*-apo.

### Data collection and processing

All datasets were collected at the Advanced Light Source (Berkeley, CA) beam line 8.2.2. using an ADSC Q315R CCD area detector. The Fen49 and Fen49*-Apo datasets were processed in HKL2000 (Otwinowski and Minor, 1997). The Fen49*-Complex dataset was processed in XDS (Kabsch, 2010).

Fen49 – Diffraction data were collected over 220° with 1° oscillations, 1 s exposures, at 100K and at a wavelength of 0.75141 Å and a crystal-to-detector distance of 156 mm. Images were processed to 1.00 Å in space group P2_1_.

Fen49*-apo – Diffraction data were collected over 220° with 1° oscillations, 1 s exposures, at 100K, a wavelength of 0.976246 Å and a crystal-to-detector distance of 210 mm. Images were processed to 1.79 Å in space group P2_1_2_1_2_1_.

Fen49*-complex – Diffraction data were collected over 135° with 0.5° oscillations, 1 s exposures, at 100K, a wavelength of 0.999878 Å and a crystal-to-detector distance of 190 mm. Images were processed to 1.67 Å in space group P2_1_2_1_2_1_.

### Structure determination and refinement

All structures were solved by molecular replacement (MR) using PHASER (McCoy et al., 2007) in the PHENIX software suite (Adams et al., 2010). Iterative rounds of manual building and refinement were conducted in Coot (Emsley et al., 2010) and Phenix.refine (Afonine et al., 2012), respectively. Hydrogens were added for all refinement jobs. The geometric quality of the final models was verified using the MolProbity server (Chen et al., 20092010). Resolution cutoffs were determined by monitoring the refinement statistics in the context of the reflection data completeness and the CC ½ and I/σI values (Karplus and Diederichs, 2012).

Fen49 – The Fen49 design model, with residues 63, 85–95 and 116–122 omitted, was used as search model for MR. Two copies of Fen49 were placed in the asymmetric unit (AU). An initial model was generated using the PHENIX Autobuild module. All defaults were used, with the following exceptions: ‘Build-in-place’ was set to ‘False’, simulated annealing was used for refinement, and prime-and-switch maps were used during model building to remove search model bias. All atoms except hydrogen were refined with anisotropic atomic displacement parameters.

Fen49*-apo – PDB 2QZ3, the parent scaffold from which Fen49 was designed, was used as the MR search model. Three copies were placed in the AU. Manual rebuilding was conducted directly from the MR solution. Poor electron density was observed for the third Fen49* copy in the AU, suggesting a large degree of disorder for this domain.

Fen49*-complex – Fen49*-apo was used as the MR search model. Manual rebuilding was conducted directly from the MR solution. Restraints for fentanyl were generated in Phenix.elbow (Moriarty et al., 2009) from the SMILES string using the eLBOW AM1 geometry optimization option.

Data collection and refinement statistics are given in Supplementary Table 6 (Supplementary file 6).

### *Arabidopsis thaliana* transcription factor reporter plasmid construction

The Fen49 and Fen21 transcription factors were engineered by N-terminal fusion of the yeast MATα gene degron and the Gal4 DNA binding domain and C-terminal fusion of the VP16 transcriptional activator to either Fen49 or Fen21. The resulting gene sequence was codon-optimized for optimal expression in *Arabidopsis thaliana* plants and cloned downstream of the CaMV35S promoter to drive constitutive expression in plants, and upstream of the octopine synthase (*ocs*) transcriptional terminator sequence. To quantify the transcriptional activation function of the Fen49 and Fen21 transcription factors, the luciferase gene from *Photinus pyralis* (firefly) was placed downstream of a synthetic plant promoter consisting of five tandem copies of a Gal4 upstream activating sequence (UAS) fused to the minimal (−46) CaMV35S promoter sequence. Transcription of luciferase is terminated by the E9 terminator sequence. These sequences were cloned into a pSEVA 141 plasmid and used for transient expression assays in *Arabidopsis* protoplasts.

### TF-Biosensor assays in protoplasts and intact plants

We next inserted the genetic circuit for Fen21 transcription and luciferase reporting into the pCAMBIA 2300 plant transformation vector and stably transformed them into *Arabidopsis thaliana* ecotype Columbia plants using a standard *Agrobacterium tumefaciens* floral dip protocol. Primary transgenic plants were screened in vivo for fentanyl-dependent luciferase production using a Stanford Photonics XR/MEGA-10Z ICCD Camera and Piper Control Software System, and responsive plants were allowed to set seed for further testing. Second generation transgenic plants (T_1_, heterozygous) were tested for fentanyl-dependent induction of luciferase expression using the same system described above.

## Additional files

Supplementary file 1. List of PDBs used as scaffold proteins.

Supplementary file 2. Overview of the 62 Fentanyl binder designs.

Supplementary file 3. Fen49 mutagenic primers.

Supplementary file 4. Fen49 site saturation mutagenesis (SSM) primers.

Supplementary file 5. Next-generation sequencing statistics for Fen49 library sorts.

Supplementary file 6. Crystallographic data collection and refinement statistics.

Transparent reporting form
