# Supplementary material for: Computational design of environmental sensors for the potent opioid fentanyl
Source: eLife. 2017 Sep 19;6:e28909. doi: 10.7554/eLife.28909 (PMC5655540; doi:10.7554/eLife.28909)
Supplement: Supplementary file 1. [file elife-28909-supp1.docx]

**Supplementary Table 1 | List of PDBs used as scaffold proteins**

**MOAD PDB set**

| 1a28 | 1a2b | 1a2t | 1a2u | 1a3k | 1a3t | 1a3u | 1a3v | 1a4m | 1a4r |
| --- | --- | --- | --- | --- | --- | --- | --- | --- | --- |
| 1a53 | 1a55 | 1a7x | 1aax | 1abe | 1abf | 1abn | 1ac8 | 1add | 1ads |
| 1aeb | 1aed | 1aef | 1aeg | 1aeh | 1aej | 1aek | 1aem | 1aeq | 1aer |
| 1aex | 1ai9 | 1aj6 | 1ake | 1akq | 1akr | 1akt | 1aku | 1akv | 1akw |
| 1aky | 1am1 | 1anc | 1and | 1ane | 1ank | 1aoe | 1apb | 1as0 | 1as3 |
| 1aua | 1avn | 1az1 | 1azl | 1b0u | 1b2r | 1b42 | 1b56 | 1bap | 1bg2 |
| 1bh2 | 1bir | 1bjk | 1bk0 | 1bkm | 1bky | 1blz | 1bof | 1bqe | 1br5 |
| 1br6 | 1btn | 1bu4 | 1bu5 | 1bx0 | 1bx1 | 1bx4 | 1bxg | 1byg | 1byq |
| 1bzc | 1bzj | 1bzs | 1c1x | 1c21 | 1c22 | 1c23 | 1c24 | 1c27 | 1c2t |
| 1c3e | 1c3j | 1c7e | 1c7f | 1c80 | 1c81 | 1c83 | 1c84 | 1c86 | 1c87 |
| 1c88 | 1c9h | 1c9w | 1cbq | 1cbs | 1cci | 1cd2 | 1cde | 1cen | 1ch0 |
| 1cip | 1cjw | 1cm8 | 1cr1 | 1cr2 | 1cr4 | 1ctq | 1cx4 | 1czh | 1czk |
| 1czl | 1czn | 1czo | 1czr | 1czu | 1d03 | 1d04 | 1d0k | 1d0l | 1d0m |
| 1d1q | 1d2s | 1d4o | 1d5c | 1d7x | 1daj | 1daw | 1day | 1db1 | 1dbp |
| 1dcp | 1dd6 | 1dds | 1dgm | 1dhi | 1dhj | 1dmw | 1dpf | 1dpo | 1dqe |
| 1dra | 1drb | 1drh | 1drj | 1drk | 1drt | 1dry | 1ds1 | 1dtl | 1dvr |
| 1dyh | 1dyi | 1dyj | 1dyr | 1e26 | 1e3g | 1e4v | 1e4y | 1e62 | 1e63 |
| 1e64 | 1ecv | 1efd | 1ej0 | 1ej1 | 1ej3 | 1ejn | 1ek0 | 1el3 | 1eom |
| 1eq2 | 1eqa | 1eqm | 1esz | 1ex7 | 1ex8 | 1exa | 1exx | 1ezf | 1f4p |
| 1f5f | 1f5k | 1f5l | 1f6b | 1f76 | 1f9h | 1f9u | 1f9v | 1f9w | 1fao |
| 1fby | 1fbz | 1fcx | 1fcy | 1fcz | 1fd0 | 1fdk | 1fdr | 1fgy | 1fh7 |
| 1fh8 | 1fh9 | 1fhd | 1fhv | 1fkw | 1fkx | 1fl2 | 1fla | 1fld | 1fln |
| 1flz | 1fm4 | 1frb | 1frn | 1frq | 1ftl | 1ftn | 1fue | 1fvt | 1fvx |
| 1fw9 | 1fys | 1fzq | 1fzu | 1g02 | 1g0e | 1g0h | 1g0i | 1g16 | 1g17 |
| 1g1b | 1g1d | 1g27 | 1g2a | 1g3m | 1g3q | 1g42 | 1g45 | 1g46 | 1g48 |
| 1g4j | 1g4o | 1g4p | 1g4s | 1g4t | 1g52 | 1g53 | 1g54 | 1g55 | 1g5f |
| 1g5n | 1g67 | 1g69 | 1g6c | 1g74 | 1g7f | 1g7g | 1g81 | 1g8z | 1g9r |
| 1ga8 | 1gaw | 1gdd | 1gfy | 1ghe | 1gia | 1giu | 1gjr | 1gkl | 1glg |
| 1gny | 1go2 | 1gr1 | 1gs4 | 1gsp | 1gu3 | 1gui | 1gvg | 1gwl | 1gwm |
| 1gwn | 1gwv | 1gww | 1gx0 | 1gx4 | 1gxo | 1gxz | 1gye | 1gym | 1gzf |
| 1h0a | 1h10 | 1h1d | 1h1h | 1h42 | 1h4o | 1h4w | 1h6h | 1h70 | 1h7l |
| 1h7q | 1h85 | 1hb1 | 1hb2 | 1hb3 | 1hb4 | 1hd2 | 1hdo | 1he2 | 1he3 |
| 1he4 | 1he5 | 1hfp | 1hfq | 1hfr | 1hi3 | 1hi4 | 1hi5 | 1hms | 1hmt |
| 1hn4 | 1hnn | 1hq2 | 1hqt | 1hsl | 1htw | 1huq | 1hxd | 1hy3 | 1hye |
| 1hyf | 1hz1 | 1i05 | 1i06 | 1i0v | 1i1n | 1i1o | 1i2e | 1i2f | 1i2g |
| 1i37 | 1i38 | 1i3f | 1i3i | 1i3u | 1i53 | 1i58 | 1i59 | 1i5a | 1i5b |
| 1i5c | 1i76 | 1i7e | 1i7g | 1i7i | 1i7p | 1i82 | 1i8a | 1i8z | 1i90 |
| 1i91 | 1i9l | 1i9m | 1i9n | 1i9o | 1i9p | 1i9q | 1i9z | 1ia1 | 1ia2 |
| 1ia3 | 1ib0 | 1id0 | 1ie8 | 1ie9 | 1iei | 1if4 | 1if5 | 1if6 | 1if7 |
| 1if8 | 1if9 | 1ig1 | 1ii6 | 1ijr | 1in4 | 1in5 | 1in6 | 1in7 | 1in8 |
| 1ion | 1ipb | 1ipc | 1is3 | 1is4 | 1it6 | 1iub | 1iuc | 1ix1 | 1ixi |
| 1iy7 | 1j01 | 1j1g | 1j1m | 1j4h | 1j4i | 1j4r | 1j53 | 1j54 | 1j7k |
| 1j84 | 1j8q | 1j8r | 1j8u | 1j91 | 1j96 | 1j99 | 1j9e | 1j9g | 1jah |
| 1jai | 1jaq | 1jb9 | 1jd0 | 1jd3 | 1jf0 | 1jf2 | 1jg1 | 1jg2 | 1jg4 |
| 1ji0 | 1jj7 | 1jj9 | 1jje | 1jjt | 1jjv | 1jk7 | 1jkk | 1jkl | 1jkx |
| 1jol | 1jom | 1jp4 | 1jpa | 1jpj | 1jpn | 1jqd | 1jsz | 1jt1 | 1jt2 |
| 1jte | 1juv | 1jx1 | 1jx6 | 1jxm | 1jyl | 1k2v | 1k4m | 1k4v | 1k63 |
| 1k6e | 1k6x | 1k9i | 1k9j | 1kak | 1kao | 1kav | 1kbc | 1kdk | 1kdm |
| 1kdo | 1kdp | 1kdr | 1kdt | 1kjl | 1kjr | 1klk | 1kmq | 1kms | 1kmv |
| 1knm | 1ko5 | 1ko8 | 1kpg | 1kqn | 1kqo | 1kqr | 1kqu | 1kqw | 1kqy |
| 1kqz | 1kr0 | 1kr1 | 1kr2 | 1kr5 | 1krh | 1ksk | 1ksl | 1ktg | 1kuv |
| 1kux | 1kuy | 1kw0 | 1kxm | 1ky2 | 1ky3 | 1kzl | 1kzn | 1l0c | 1l1e |
| 1l4u | 1l4y | 1l6m | 1l7k | 1l7p | 1l8b | 1l8g | 1l9k | 1lb9 | 1lbb |
| 1lbf | 1lbl | 1lc3 | 1lf5 | 1lfo | 1lgw | 1lgx | 1lhn | 1lho | 1lhu |
| 1lhv | 1lhw | 1li2 | 1li3 | 1li6 | 1lid | 1lif | 1lke | 1ln1 | 1lnm |
| 1lov | 1low | 1loy | 1lp4 | 1lpc | 1lpd | 1lpu | 1lqf | 1lqy | 1lr4 |
| 1lrm | 1lru | 1ls6 | 1lt6 | 1ltz | 1lvg | 1ly3 | 1ly4 | 1m2g | 1m2h |
| 1m2j | 1m2k | 1m2p | 1m2q | 1m2r | 1m2x | 1m48 | 1m49 | 1m5c | 1m5d |
| 1m6y | 1m78 | 1m79 | 1m7a | 1m7b | 1m9h | 1mai | 1mbb | 1mc9 | 1men |
| 1mgp | 1mh1 | 1mkv | 1mlw | 1mm6 | 1mm7 | 1mmb | 1mmk | 1mmt | 1mmu |
| 1mmx | 1mmy | 1mmz | 1mn0 | 1moo | 1mqd | 1mqg | 1mqh | 1mqi | 1mqj |
| 1mqo | 1mr3 | 1mrq | 1msk | 1mt6 | 1mud | 1mvs | 1mvt | 1mwm | 1my3 |
| 1n06 | 1n07 | 1n08 | 1n0s | 1n0t | 1n2b | 1n2e | 1n2g | 1n2h | 1n2i |
| 1n2j | 1n2x | 1n46 | 1n6a | 1n6c | 1n6h | 1n6i | 1n6k | 1n6l | 1n6n |
| 1n6o | 1n6p | 1n6r | 1n8u | 1n8v | 1nae | 1nav | 1nb0 | 1nb9 | 1nd4 |
| 1ne4 | 1ne6 | 1ng1 | 1njf | 1nl9 | 1nli | 1nmk | 1nny | 1no6 | 1nq0 |
| 1nq2 | 1ns0 | 1ns4 | 1ns7 | 1ns8 | 1nsm | 1nsr | 1nss | 1nst | 1nsu |
| 1nsv | 1nsx | 1nsz | 1nuc | 1nv8 | 1nv9 | 1nw5 | 1nw6 | 1nw7 | 1nwl |
| 1nxj | 1nxy | 1ny0 | 1nz7 | 1o03 | 1o0r | 1o3y | 1o42 | 1o44 | 1o46 |
| 1o47 | 1o48 | 1o49 | 1o4b | 1o4f | 1o4g | 1o4h | 1o4j | 1o4l | 1o4m |
| 1o4n | 1o4o | 1o4p | 1o4q | 1o4r | 1o6y | 1o7o | 1o7q | 1o87 | 1o8s |
| 1o9b | 1o9v | 1o9w | 1oba | 1obn | 1obo | 1obv | 1oc1 | 1oc3 | 1ocu |
| 1od3 | 1odm | 1odn | 1of4 | 1og1 | 1ogi | 1ogj | 1oh3 | 1oh4 | 1ohj |
| 1ohk | 1oio | 1oiw | 1oix | 1oj4 | 1ojz | 1ony | 1onz | 1os2 | 1os7 |
| 1osf | 1osh | 1ot6 | 1ot9 | 1otj | 1ov5 | 1ov7 | 1ovh | 1ovj | 1ovk |
| 1ow4 | 1owy | 1owz | 1ozp | 1p0h | 1p1o | 1p1q | 1p1w | 1p2s | 1p2t |
| 1p2u | 1p2v | 1p39 | 1p3j | 1p42 | 1p4f | 1p4m | 1p6d | 1p77 | 1p99 |
| 1pa9 | 1pb7 | 1pb8 | 1pb9 | 1pbk | 1pbq | 1pd8 | 1pd9 | 1ph0 | 1phk |
| 1pjk | 1pmh | 1pno | 1pnq | 1pot | 1poy | 1pty | 1pu8 | 1puj | 1pvs |
| 1pwl | 1pwm | 1pxh | 1pyn | 1pz0 | 1pz1 | 1pz4 | 1pzo | 1pzp | 1pzx |
| 1q0n | 1q0s | 1q13 | 1q1y | 1q1z | 1q20 | 1q22 | 1q3a | 1q5m | 1q6j |
| 1q6m | 1q6n | 1q6p | 1q6s | 1q6t | 1q9s | 1qan | 1qd1 | 1qf9 | 1qfy |
| 1qfz | 1qg0 | 1qga | 1qgq | 1qgs | 1qgy | 1qgz | 1qh0 | 1qiq | 1qje |
| 1qjf | 1ql6 | 1qra | 1qsr | 1qut | 1qv0 | 1qv1 | 1qvj | 1qx4 | 1qxa |
| 1qxk | 1qxw | 1qxy | 1qxz | 1qy1 | 1qy2 | 1qy5 | 1qy8 | 1qye | 1r18 |
| 1r2q | 1r5l | 1r6n | 1r6w | 1ra1 | 1ra2 | 1ra3 | 1ra8 | 1ra9 | 1rao |
| 1rb0 | 1rb2 | 1rb3 | 1rbm | 1rbq | 1rby | 1rbz | 1rc1 | 1rc4 | 1rd4 |
| 1re8 | 1rej | 1rek | 1rf7 | 1rg7 | 1rgc | 1rgk | 1rgl | 1rh3 | 1rhl |
| 1ri1 | 1ri3 | 1ri4 | 1rjd | 1rje | 1rkp | 1rlj | 1rm8 | 1rmz | 1ros |
| 1rpj | 1rqj | 1ru1 | 1ru2 | 1rx1 | 1rx2 | 1rx3 | 1rx4 | 1rx5 | 1rx6 |
| 1rx7 | 1rx9 | 1ry0 | 1ry8 | 1ryc | 1s0z | 1s14 | 1s17 | 1s19 | 1s1d |
| 1s1j | 1s1p | 1s1r | 1s1s | 1s2c | 1s36 | 1s3g | 1s3u | 1s3v | 1s3w |
| 1s3y | 1s4m | 1s50 | 1s68 | 1s7n | 1s9j | 1s9t | 1sc8 | 1sd3 | 1skj |
| 1sl4 | 1sl5 | 1sl9 | 1sln | 1sm4 | 1so0 | 1sqn | 1sr7 | 1svi | 1sw1 |
| 1sww | 1syh | 1syi | 1t27 | 1t2w | 1t3t | 1t40 | 1t41 | 1t48 | 1t49 |
| 1t4g | 1t5c | 1t6x | 1t6z | 1t7d | 1t7t | 1t91 | 1t9f | 1t9s | 1ta8 |
| 1tb7 | 1tbb | 1tbf | 1tdr | 1tfj | 1tg2 | 1tip | 1tj5 | 1tjy | 1tke |
| 1tkg | 1tky | 1tmm | 1tou | 1tow | 1tpy | 1tqm | 1tqp | 1tr5 | 1tr7 |
| 1tt1 | 1tt2 | 1tt8 | 1ttm | 1tu4 | 1tuv | 1tvy | 1tw1 | 1tw5 | 1txf |
| 1txi | 1txz | 1ty8 | 1tzd | 1u0j | 1u0y | 1u0z | 1u1b | 1u25 | 1u26 |
| 1u27 | 1u29 | 1u2o | 1u2s | 1u31 | 1u32 | 1u3f | 1u3g | 1u70 | 1u71 |
| 1u72 | 1u7w | 1u7z | 1u8a | 1u8u | 1u8y | 1u8z | 1u90 | 1u9n | 1u9q |
| 1udt | 1uhh | 1uhj | 1uhk | 1ui0 | 1uio | 1uip | 1ujp | 1uk6 | 1uk7 |
| 1uk8 | 1uka | 1ukb | 1uke | 1umi | 1umk | 1unq | 1uoz | 1up0 | 1up2 |
| 1up3 | 1upr | 1upv | 1upw | 1urx | 1us0 | 1us4 | 1us5 | 1usk | 1utz |
| 1uwf | 1uwp | 1ux7 | 1uxx | 1uxy | 1uy0 | 1uy2 | 1uy3 | 1uy4 | 1uy6 |
| 1uy7 | 1uy8 | 1uy9 | 1uyc | 1uyd | 1uye | 1uyf | 1uyg | 1uyh | 1uyi |
| 1uyk | 1uym | 1uyy | 1uyz | 1uz0 | 1uzw | 1v00 | 1v0c | 1v0o | 1v0p |
| 1v2g | 1v2j | 1v2k | 1v2l | 1v2m | 1v2n | 1v2o | 1v2p | 1v2q | 1v2r |
| 1v2s | 1v2t | 1v2u | 1v2v | 1v2w | 1v39 | 1v7r | 1va5 | 1vbj | 1vc8 |
| 1vc9 | 1ve3 | 1vg1 | 1vg8 | 1vhl | 1vhn | 1vht | 1vid | 1vj3 | 1vj9 |
| 1vja | 1vkj | 1vm1 | 1vp5 | 1vp6 | 1vyf | 1vyg | 1vzt | 1vzu | 1vzx |
| 1w03 | 1w04 | 1w05 | 1w06 | 1w0h | 1w0z | 1w11 | 1w13 | 1w1a | 1w1d |
| 1w1g | 1w2c | 1w2d | 1w34 | 1w35 | 1w3v | 1w3x | 1w58 | 1w5x | 1w6f |
| 1w8l | 1w8m | 1w8t | 1w8u | 1w9q | 1w9t | 1w9w | 1wax | 1wb4 | 1wb5 |
| 1wb6 | 1wbe | 1wdi | 1wdy | 1we2 | 1wei | 1wf3 | 1wg8 | 1wk9 | 1wld |
| 1wlj | 1wm1 | 1wma | 1wq3 | 1wq4 | 1ws1 | 1wub | 1wvj | 1wxj | 1wy7 |
| 1wzu | 1x0p | 1x1r | 1x1s | 1x71 | 1x89 | 1x8i | 1x8u | 1x8x | 1x96 |
| 1x97 | 1x98 | 1xap | 1xbo | 1xc1 | 1xcj | 1xcl | 1xcm | 1xdd | 1xdg |
| 1xdn | 1xdy | 1xep | 1xf0 | 1xff | 1xfg | 1xj0 | 1xjd | 1xk5 | 1xk9 |
| 1xlr | 1xmj | 1xnn | 1xnz | 1xom | 1xon | 1xoq | 1xor | 1xov | 1xoz |
| 1xpz | 1xq0 | 1xq3 | 1xq6 | 1xqp | 1xqw | 1xqx | 1xr1 | 1xro | 1xrr |
| 1xs5 | 1xt8 | 1xtp | 1xtq | 1xu4 | 1xuo | 1xw4 | 1xws | 1xyp | 1xzc |
| 1y1p | 1y1z | 1y20 | 1y2b | 1y2c | 1y2e | 1y2f | 1y2g | 1y2k | 1y59 |
| 1y5a | 1y5b | 1y5u | 1y63 | 1y65 | 1y7i | 1y93 | 1y9q | 1y9r | 1ybm |
| 1ybu | 1yc1 | 1yc2 | 1yc3 | 1yc4 | 1yet | 1yhs | 1yi3 | 1yi4 | 1yj3 |
| 1yjq | 1ynd | 1yns | 1yob | 1yon | 1yp6 | 1yre | 1yt0 | 1yu9 | 1yum |
| 1yun | 1yvd | 1yvm | 1yxu | 1yxv | 1yxx | 1yz3 | 1yzg | 1yzk | 1yzl |
| 1yzn | 1yzq | 1yzt | 1yzu | 1z06 | 1z07 | 1z08 | 1z0d | 1z0f | 1z0i |
| 1z16 | 1z17 | 1z18 | 1z22 | 1z2a | 1z2n | 1z2o | 1z2p | 1z3c | 1z3n |
| 1z4n | 1z4o | 1z4r | 1z57 | 1z6y | 1z83 | 1z89 | 1z8a | 1z95 | 1zao |
| 1zar | 1zb6 | 1zcw | 1zd9 | 1zdw | 1ze8 | 1zgd | 1zh0 | 1zh6 | 1zhs |
| 1zj6 | 1zk5 | 1zkn | 1zly | 1znd | 1zng | 1znx | 1zny | 1znz | 1zoe |
| 1zog | 1zoh | 1zp5 | 1zp9 | 1zq5 | 1zq9 | 1zrh | 1zs0 | 1zsb | 1ztf |
| 1zth | 1ztq | 1zuc | 1zui | 1zvq | 1zvx | 1zw6 | 1zx5 | 1zxc | 220l |
| 223l | 225l | 227l | 2a14 | 2a1l | 2a1x | 2a5j | 2a5s | 2a8h | 2a9j |
| 2aa2 | 2aa5 | 2aa7 | 2aac | 2aax | 2abe | 2aco | 2acq | 2acr | 2acs |
| 2acu | 2ada | 2ae7 | 2aeb | 2aec | 2aes | 2af9 | 2ag2 | 2ag4 | 2ag6 |
| 2ag9 | 2agc | 2agd | 2agt | 2ah9 | 2ahc | 2ai7 | 2ai8 | 2aia | 2aie |
| 2aix | 2ajh | 2aky | 2al5 | 2al7 | 2am9 | 2ama | 2amb | 2anq | 2anz |
| 2aot | 2aou | 2aov | 2aqd | 2arc | 2art | 2aru | 2as1 | 2as2 | 2as3 |
| 2as4 | 2as6 | 2ath | 2atj | 2awh | 2ax6 | 2ax7 | 2ax8 | 2ax9 | 2axa |
| 2azr | 2azy | 2azz | 2b00 | 2b01 | 2b03 | 2b04 | 2b07 | 2b1q | 2b1r |
| 2b50 | 2b96 | 2baw | 2bb7 | 2bcd | 2bdg | 2bdx | 2bej | 2bel | 2bfq |
| 2bfr | 2bgd | 2bge | 2bgi | 2bgj | 2bgs | 2bgu | 2bik | 2bkt | 2bkv |
| 2bkx | 2bl9 | 2bla | 2blc | 2bln | 2bmd | 2bme | 2bmv | 2bmw | 2bod |
| 2bof | 2bog | 2br6 | 2brc | 2bre | 2bs7 | 2bs8 | 2bsa | 2bsb | 2bsc |
| 2bsw | 2bsy | 2bu4 | 2bu9 | 2bue | 2bv7 | 2bvd | 2bwa | 2bwc | 2byc |
| 2byh | 2byi | 2bzg | 2bzz | 2c01 | 2c02 | 2c05 | 2c27 | 2c2s | 2c2t |
| 2c53 | 2c6d | 2c6e | 2c80 | 2c8a | 2c8f | 2c8m | 2c91 | 2c96 | 2c98 |
| 2c99 | 2c9c | 2cb3 | 2cbo | 2cbs | 2cbt | 2cbz | 2ccs | 2cct | 2cd2 |
| 2cdd | 2cdn | 2cdz | 2ce2 | 2cgn | 2chg | 2chu | 2cig | 2cip | 2cis |
| 2cjp | 2cl0 | 2cl5 | 2cl6 | 2cl7 | 2clc | 2cld | 2clq | 2cm7 | 2cm8 |
| 2cma | 2cmb | 2cmc | 2cmk | 2cne | 2cnf | 2cng | 2cnh | 2cni | 2cns |
| 2cnt | 2csn | 2cul | 2d06 | 2d0k | 2d1n | 2d1o | 2d2f | 2d2v | 2d3y |
| 2d5a | 2d5c | 2d6n | 2d6o | 2dfn | 2dg3 | 2dg4 | 2dg9 | 2djh | 2dm5 |
| 2doo | 2dpm | 2dpx | 2drc | 2dri | 2drz | 2ds0 | 2duq | 2dur | 2dux |
| 2duz | 2dv0 | 2dvz | 2e2r | 2e2x | 2e3n | 2e3o | 2e3p | 2e3q | 2e3r |
| 2e5a | 2e5y | 2e6u | 2e6v | 2e9n | 2e9o | 2e9s | 2ea1 | 2eg7 | 2eg8 |
| 2eqa | 2eu2 | 2eu3 | 2eu8 | 2eug | 2euk | 2eum | 2eun | 2euo | 2eup |
| 2euq | 2eur | 2eus | 2eut | 2euu | 2evc | 2evd | 2evl | 2evm | 2evs |
| 2evw | 2ew1 | 2ew5 | 2ew6 | 2ez7 | 2f1j | 2f2g | 2f2k | 2f32 | 2f38 |
| 2f47 | 2f4b | 2f4j | 2f5x | 2f6t | 2f6v | 2f6w | 2f6y | 2f6z | 2f70 |
| 2f71 | 2f78 | 2f8p | 2f90 | 2f9l | 2f9m | 2faq | 2far | 2fax | 2fc0 |
| 2fct | 2fcu | 2fcv | 2fdj | 2fdx | 2fe4 | 2ff7 | 2ffa | 2ffb | 2ffq |
| 2fgb | 2fjm | 2fjn | 2fk8 | 2flh | 2flv | 2fmx | 2fn8 | 2fnn | 2foq |
| 2fos | 2fou | 2fov | 2foy | 2fpk | 2fpl | 2fpm | 2fqw | 2fqx | 2fqy |
| 2fr3 | 2fu8 | 2fu9 | 2fue | 2fvx | 2fvy | 2fwz | 2fyb | 2fz8 | 2fz9 |
| 2fzb | 2fzd | 2fzh | 2fzi | 2fzj | 2g0h | 2g19 | 2g1k | 2g1m | 2g1r |
| 2g1s | 2g1y | 2g21 | 2g22 | 2g24 | 2g2h | 2g2y | 2g37 | 2g6p | 2g70 |
| 2g71 | 2g72 | 2g78 | 2g79 | 2g7b | 2g7c | 2g8n | 2gal | 2gbp | 2gcn |
| 2gco | 2gcp | 2gdj | 2gfe | 2gfj | 2gfk | 2gg0 | 2gg2 | 2gg3 | 2gg5 |
| 2gg7 | 2gg8 | 2gg9 | 2ggb | 2ggc | 2ghl | 2ghm | 2gil | 2gj8 | 2gj9 |
| 2gja | 2gkl | 2gmk | 2gp5 | 2gqt | 2gqu | 2gsp | 2gte | 2gtf | 2gtm |
| 2gtn | 2gtx | 2gu4 | 2gu5 | 2gu6 | 2gv6 | 2gv7 | 2gvv | 2gwh | 2gwl |
| 2h02 | 2h03 | 2h04 | 2h1f | 2h1h | 2h29 | 2h2a | 2h42 | 2h44 | 2h4g |
| 2h4k | 2h4n | 2h4x | 2h4z | 2h52 | 2h55 | 2h77 | 2h79 | 2ham | 2har |
| 2has | 2haw | 2hb1 | 2hb7 | 2hb8 | 2hdj | 2hej | 2hf8 | 2hf9 | 2hfo |
| 2hfu | 2hhj | 2hk5 | 2hlv | 2hnx | 2hps | 2ht6 | 2hu6 | 2huo | 2hv5 |
| 2hvc | 2hvn | 2hvo | 2hw2 | 2hwq | 2hwr | 2hxm | 2hyu | 2hyv | 2hzq |
| 2i16 | 2i17 | 2i1q | 2i2s | 2i3i | 2i3v | 2i3w | 2i42 | 2i4g | 2i4h |
| 2i4j | 2i4p | 2i4z | 2i5c | 2i5f | 2i5x | 2i6a | 2i6b | 2i6p | 2i74 |
| 2i7o | 2ibn | 2ica | 2ick | 2idv | 2ihq | 2ikg | 2ikh | 2iki | 2ikj |
| 2il4 | 2img | 2ine | 2inz | 2iok | 2ior | 2ioy | 2ipf | 2ipg | 2ipj |
| 2ipw | 2iq0 | 2iqd | 2irx | 2iry | 2is7 | 2isf | 2it5 | 2it6 | 2iuw |
| 2ivi | 2ivj | 2ivn | 2ivp | 2iwx | 2ixu | 2ixv | 2iyl | 2iyq | 2iyr |
| 2iys | 2iyu | 2iyv | 2iyw | 2iyx | 2iyy | 2iyz | 2j0v | 2j1a | 2j1e |
| 2j1s | 2j1t | 2j1u | 2j1v | 2j44 | 2j4a | 2j65 | 2j72 | 2j73 | 2j7m |
| 2jai | 2jaj | 2jav | 2jb4 | 2jc4 | 2jcj | 2jck | 2jcq | 2jcr | 2jdc |
| 2jdd | 2jfn | 2jfp | 2jh7 | 2jhd | 2jig | 2lbd | 2mbr | 2ng1 | 2nmn |
| 2nmo | 2nmx | 2nn1 | 2nn7 | 2nn8 | 2nng | 2nno | 2nnq | 2nns | 2nnv |
| 2nq6 | 2nq7 | 2nst | 2nt6 | 2nt7 | 2nt9 | 2nta | 2ntb | 2ntp | 2ntq |
| 2nuc | 2nun | 2nvc | 2nvd | 2nxe | 2nyr | 2o1c | 2o3p | 2o3z | 2o63 |
| 2o64 | 2o7n | 2oax | 2ob2 | 2obf | 2obj | 2obm | 2oci | 2ofp | 2oh4 |
| 2ohv | 2oi4 | 2oiq | 2ojt | 2okl | 2oo8 | 2orw | 2oty | 2otz | 2ou0 |
| 2oun | 2ouq | 2our | 2ouu | 2ovd | 2ovx | 2ovz | 2ow0 | 2ow1 | 2ox9 |
| 2oxd | 2oxp | 2oxx | 2oxy | 2oz5 | 2oz7 | 2p0c | 2p0d | 2p0e | 2p0h |
| 2p1e | 2p2v | 2p39 | 2p3l | 2p41 | 2p4y | 2p6w | 2p73 | 2p7a | 2p7g |
| 2p7z | 2p8n | 2p98 | 2p99 | 2p9a | 2pd5 | 2pd7 | 2pd8 | 2pd9 | 2pdb |
| 2pdc | 2pdf | 2pdg | 2pdh | 2pdi | 2pdj | 2pdk | 2pdl | 2pdm | 2pdn |
| 2pdp | 2pdq | 2pdr | 2pdt | 2pdu | 2pdw | 2pdx | 2pdy | 2pez | 2pfg |
| 2pfy | 2pfz | 2pg2 | 2pin | 2pio | 2pip | 2piq | 2pir | 2pit | 2piu |
| 2piv | 2pix | 2pjo | 2pkr | 2pl3 | 2pmk | 2pnu | 2pql | 2prb | 2pvr |
| 2pvu | 2pw3 | 2pwl | 2px2 | 2px4 | 2px5 | 2px8 | 2pxa | 2pxh | 2py3 |
| 2pys | 2pyu | 2pyy | 2pz5 | 2pzp | 2q1h | 2q1v | 2q2a | 2q2c | 2q37 |
| 2q46 | 2q4b | 2q4x | 2q4y | 2q6m | 2q88 | 2q89 | 2q8s | 2q92 | 2q93 |
| 2q94 | 2q95 | 2q96 | 2q9b | 2q9c | 2qb5 | 2qbp | 2qbq | 2qbr | 2qbs |
| 2qc6 | 2qds | 2qdx | 2qeb | 2qeh | 2qen | 2qeo | 2qet | 2qfo | 2qg0 |
| 2qg2 | 2qg6 | 2qhs | 2qhu | 2qhv | 2qim | 2qir | 2qj7 | 2qk8 | 2qm9 |
| 2qo4 | 2qo5 | 2qo6 | 2qoc | 2qoh | 2qq0 | 2qry | 2qsy | 2qsz | 2qt0 |
| 2quz | 2qv7 | 2qw8 | 2qx7 | 2qxw | 2qyl | 2qyn | 2qz3 | 2qzt | 2qzz |
| 2r09 | 2r0d | 2r2g | 2r2w | 2r3d | 2r4v | 2r58 | 2r5a | 2r6j | 2r6r |
| 2r75 | 2rar | 2rav | 2ray | 2raz | 2rb0 | 2rb1 | 2rb2 | 2rb5 | 2rbk |
| 2rbn | 2rbo | 2rbp | 2rbq | 2rbr | 2rbs | 2rbt | 2rbu | 2rbv | 2rbw |
| 2rbx | 2rby | 2rbz | 2rc0 | 2rc1 | 2rc2 | 2rc5 | 2rc9 | 2rcb | 2rct |
| 2rdg | 2rdk | 2rdn | 2rdq | 2rdr | 2rds | 2reg | 2rga | 2rgb | 2rgc |
| 2rgd | 2rge | 2rgg | 2rgx | 2rhu | 2rhx | 2rhy | 2rin | 2rnf | 2ukd |
| 2uus | 2uwn | 2uyq | 2v05 | 2v0u | 2v1a | 2v2e | 2v2q | 2v2v | 2v2z |
| 2v34 | 2v3u | 2v54 | 2v5u | 2v5v | 2v72 | 2v73 | 2v8e | 2v8p | 2vau |
| 2vbb | 2vbu | 2vbv | 2vcf | 2vcn | 2vcs | 2vdf | 2vdg | 2veg | 2vek |
| 2vel | 2veu | 2vev | 2vew | 2vex | 2vey | 2vf3 | 2vfk | 2vfl | 2vgd |
| 2vin | 2vio | 2vip | 2viq | 2viv | 2viw | 2vmg | 2vn1 | 2vng | 2vnh |
| 2vni | 2vnj | 2vnk | 2vno | 2vnp | 2vnt | 2vp3 | 2vpn | 2vpo | 2vqy |
| 2vs3 | 2vs4 | 2vs5 | 2vw5 | 2vwa | 2vwc | 2vyt | 2w3l | 2yx1 | 2yz3 |
| 2z5f | 2z60 | 2z8l | 2z9y | 2z9z | 2zcp | 2zcq | 2zcr | 2zcs | 2zcv |
| 2zd8 | 2ze5 | 2ze6 | 2ze7 | 2zex | 2zey | 2zgy | 2zgz | 2zhk | 2zhl |
| 2zhm | 2zhn | 2zkn | 2zlz | 2zmm | 2zn7 | 2zpt | 2zqo | 2zr9 | 2zvp |
| 2zvq | 3aky | 3b2r | 3b3c | 3b50 | 3b5j | 3b5r | 3b65 | 3b66 | 3b67 |
| 3b68 | 3b6j | 3b6q | 3b6t | 3b6w | 3b74 | 3b7i | 3b7n | 3b7q | 3b7z |
| 3b8y | 3b8z | 3b9q | 3bcj | 3bd9 | 3bdq | 3bf8 | 3bft | 3bfv | 3bfx |
| 3bgd | 3bgi | 3bgq | 3bgz | 3bhi | 3bhj | 3bhm | 3bia | 3bib | 3bir |
| 3bki | 3bm9 | 3bmy | 3bqc | 3bqf | 3bqm | 3bqn | 3brn | 3bu1 | 3bu4 |
| 3bur | 3buv | 3bv7 | 3bwf | 3bwm | 3bwy | 3bxd | 3bxe | 3bxg | 3bxh |
| 3by9 | 3c13 | 3c1t | 3c3u | 3c3x | 3c4f | 3c8f | 3caq | 3cas | 3cav |
| 3cbs | 3cd2 | 3ciw | 3cix | 3clp | 3cm2 | 3cmf | 3cnl | 3cnn | 3cno |
| 3cot | 3crz | 3cs4 | 3cs6 | 3cse | 3csz | 3ct0 | 3ct1 | 3ct5 | 3ctq |
| 3cwe | 3cyi | 3cyu | 3czv | 3d14 | 3d27 | 3d6d | 3d6u | 3d6v | 3d80 |
| 3d84 | 3daj | 3def | 3dha | 3dhb | 3dhc | 3dln | 3dop | 3dp4 | 3dqw |
| 3dqx | 3drc | 3dy8 | 3dyn | 3dyq | 3dys | 3e0b | 3e4o | 3E+70 | 3ecn |
| 3eeb | 3ejw | 3eko | 3ekr | 3el8 | 3eld | 3eln | 3elu | 3elw | 3ely |
| 3emb | 3emd | 3erk | 3f15 | 3f16 | 3f17 | 3f18 | 3f19 | 3f1a | 3f61 |
| 3f6x | 3gal | 3gsp | 3kar | 3lbd | 3lkf | 3mag | 3mct | 3nll | 3nuc |
| 3pah | 3rab | 3ukd | 4bu4 | 4cd2 | 4dcg | 4dfr | 4erk | 4gal | 4gsp |
| 4lbd | 4nll | 4nul | 4pah | 4rsk | 4ukd | 5abp | 5bu4 | 5eug | 5gsp |
| 5nuc | 5nul | 5pah | 5ukd | 6abp | 6dfr | 6gsp | 6nul | 6pah | 7abp |
| 7dfr | 8abp | 966c | 9abp |  |  |  |  |  |  |

**Homologs set**

| 1a18 | 1a40 | 1a53 | 1abe | 1aj0 | 1ajk | 1anf | 1aw1 | 1b54 | 1btm |
| --- | --- | --- | --- | --- | --- | --- | --- | --- | --- |
| 1btm | 1d9e | 1dbt | 1dl3 | 1dl3 | 1dqw | 1dvj | 1dxe | 1e1a | 1e3v |
| 1ebg | 1eg9 | 1eix | 1eua | 1eye | 1f5j | 1f5z | 1fhv | 1ftx | 1fwt |
| 1g69 | 1g7u | 1gca | 1gpw | 1gqn | 1gth | 1gvf | 1gy7 | 1gyh | 1h0b |
| 1h1a | 1h1y | 1h5y | 1h61 | 1hg3 | 1hkx | 1ho1 | 1hsl | 1i45 | 1i4n |
| 1i4n | 1i60 | 1igs | 1iub | 1ixh | 1izc | 1j2w | 1jcj | 1jcl | 1jkg |
| 1jn5 | 1juk | 1jul | 1jvx | 1k32 | 1k3u | 1ka9 | 1kqy | 1l6w | 1lbf |
| 1lbf | 1lbl | 1lst | 1m1b | 1m3u | 1m4w | 1m6j | 1mac | 1mo0 | 1mpd |
| 1mve | 1mzh | 1n4a | 1n7k | 1nsj | 1nu3 | 1o0y | 1o1z | 1o60 | 1o66 |
| 1o68 | 1of5 | 1oho | 1ok6 | 1oqf | 1oy0 | 1p0k | 1p6o | 1pjx | 1pvx |
| 1q40 | 1q7f | 1qap | 1qds | 1qma | 1qo2 | 1qo2 | 1qpn | 1qwg | 1rd5 |
| 1ri6 | 1rpx | 1rv8 | 1rwi | 1s18 | 1s5a | 1sfj | 1sfs | 1sgj | 1sjw |
| 1sux | 1thf | 1thf | 1tml | 1tp6 | 1tqj | 1tqx | 1tre | 1tsn | 1tuh |
| 1u83 | 1ujp | 1v5x | 1v93 | 1vc4 | 1vcv | 1vd6 | 1vhc | 1viz | 1vkf |
| 1vlw | 1vpx | 1vqt | 1vs1 | 1w0m | 1w37 | 1wbh | 1wdn | 1wq5 | 1wql |
| 1wx0 | 1x1o | 1x7i | 1xbz | 1xc4 | 1xi3 | 1xm3 | 1xrl | 1y0e | 1y3n |
| 1yad | 1ydn | 1yfq | 1yx1 | 1yxy | 1yya | 1z1s | 1zcc | 1zco | 1zlp |
| 1zo2 | 2a15 | 2a4a | 2agk | 2aqw | 2ayh | 2b3f | 2b4w | 2bdq | 2bhm |
| 2bmo | 2bng | 2bs5 | 2cc3 | 2chc | 2cw6 | 2cz5 | 2dp3 | 2dry | 2dso |
| 2dua | 2dyh | 2dza | 2e7f | 2ehh | 2ekc | 2f6u | 2f98 | 2fjk | 2fli |
| 2fnc | 2fwv | 2gex | 2gey | 2gh9 | 2ghs | 2gjl | 2gop | 2h6r | 2h9l |
| 2hes | 2htm | 2i5i | 2i9e | 2ia4 | 2imj | 2jbm | 2jen | 2jgq | 2nuw |
| 2nv2 | 2o62 | 2ocz | 2ojh | 2onr | 2oog | 2owp | 2ox1 | 2oxn | 2p10 |
| 2p4o | 2p9w | 2pcq | 2pz0 | 2q8z | 2qc5 | 2qe8 | 2qf7 | 2qiw | 2qiy |
| 2qjg | 2qo4 | 2r4i | 2r79 | 2r91 | 2rcd | 2rfg | 2rfr | 2rgq | 2ux0 |
| 2v30 | 2v5j | 2v81 | 2vc6 | 2vep | 2vfh | 2vpj | 2vws | 2w2c | 2w6r |
| 2woz | 2yr1 | 2ysw | 2yw3 | 2yxg | 2yyu | 2yzr | 2z2o | 2z6i | 2zbt |
| 2ze3 | 2zvr | 3b4o | 3b4u | 3b7c | 3b8i | 3b8l | 3bb9 | 3bdr | 3bg5 |
| 3blz | 3bo9 | 3c2v | 3c56 | 3c6c | 3ceu | 3cfz | 3cg1 | 3chv | 3cij |
| 3cjp | 3cnx | 3cpg | 3ct7 | 3cu2 | 3cu3 | 3cu9 | 3d9r | 3daq | 3das |
| 3dm8 | 3dmc | 3dr2 | 3dsm | 3duk | 3dxo | 3e02 | 3e49 | 3e5z | 3e99 |
| 3e9a | 3ebt | 3eby | 3ec9 | 3ecf | 3ef8 | 3ehc | 3ejv | 3ekz | 3elz |
| 3emm | 3en8 | 3er7 | 3ewb | 3exr | 3ez4 | 3f14 | 3f40 | 3f4n | 3f4w |
| 3f7s | 3f7x | 3f8a | 3f8h | 3f8x | 3f9s | 3fa5 | 3fa6 | 3fen | 3ff0 |
| 3ff2 | 3fgc | 3fgy | 3fh1 | 3fka | 3flj | 3flu | 3fm0 | 3fmz | 3fok |
| 3frx | 3fs2 | 3fsd | 3fyo | 3g0k | 3g16 | 3g8z | 3gay | 3ge2 | 3gk0 |
| 3gnn | 3grd | 3gvg | 3gwr | 3gzb | 3gzr | 3h2a | 3h3h | 3h51 | 3hfq |
| 3hk4 | 3hv9 | 3hx8 | 3hxj | 3hzp | 3i0y | 3i10 | 3ieb | 3igs | 3ih1 |
| 3ii7 | 3ii8 | 3inp | 3iwp | 3js3 | 3k0z | 3k13 | 3ke7 | 3kkg | 3krs |
| 3ks6 | 3ksp | 3kst | 3kts | 3kws | 3kxq | 3l0g | 3l2i | 4std |  |

**Ketosteroid Isomerase PDB set**

| 1jb2 | 1jb4 | 1jb5 | 1jkg | 1k41 | 1m98 | 1mwr | 1mws | 1mwt | 1mwu |
| --- | --- | --- | --- | --- | --- | --- | --- | --- | --- |
| 1ndo | 1nu3 | 1nww | 1o7g | 1o7h | 1o7m | 1o7n | 1o7p | 1o7w | 1ocv |
| 1of5 | 1ogx | 1ogz | 1oh0 | 1oho | 1ohp | 1ohs | 1opy | 1oun | 1q40 |
| 1q42 | 1qjg | 1qma | 1s5a | 1sjw | 1std | 1u5o | 1uli | 1ulj | 1uuv |
| 1uuw | 1vqq | 1vzz | 1w00 | 1w01 | 1w02 | 1w6y | 1wql | 1z1s | 1zx2 |
| 2k54 | 2owp | 2pzv | 2qiy | 2rcd | 2rfr | 2std | 2ux0 | 2w2c | 2z76 |
| 2z77 | 2z7a | 3jum | 3jun | 3juo | 3juq | 3k0z | 3k7c | 3ke7 | 3kkg |
| 3ksp | 3lyg | 3lza | 3m8c | 3mg1 | 3mg2 | 3mg3 | 3mso | 3std | 4std |
| 5std | 6std | 7std |  |  |  |  |  |  |  |
