# Supplementary material for: Computational design of environmental sensors for the potent opioid fentanyl
Source: eLife. 2017 Sep 19;6:e28909. doi: 10.7554/eLife.28909 (PMC5655540; doi:10.7554/eLife.28909)
Supplement: Supplementary file 2. [file elife-28909-supp2.docx]

**Supplementary Table 2 | Overview of the 62 Fentanyl Binder Designs**

| Design | Scaffold PDB | Mutations | Coding Sequence | Design Approach |
| --- | --- | --- | --- | --- |
| FEN1 | 2fwz | E32D N36I I76V M83W  D87T L88A  I89T N90G  N91K L92G  G93A I95A  G120W T137F  T169V | ATGGAAGTTGAAACCTTCGCATTCCAAGCAGAAATCGCCCAATTGATGTCCTTAATCATAAACACCTTTTACTCTAACAAAGAAATATTCTTGAGAGATTTGATCTCTATATCTTCAGATGCTTTGGACAAGATCAGATACGAATCATTGACTGATCCATCTAAGTTGGACTCTGGTAAAGAATTGCATATCAATTTGATCCCTAACAAGCAAGATAGAACTTTAACAGTTGTAGACACTGGTATTGGTTGGACAAAAGCCACCGCTACTGGTAAAGGTGCAACCGCTGCAAAATCTGGTACTAAGGCTTTTATGGAAGCATTGCAAGCAGGTGCCGATATATCCATGATCGGTCAATTTTGGGTTGGTTTCTATAGTGCCTACTTAGTAGCTGAAAAGGTCACTGTTATTTTTAAACACAACGATGACGAACAATACGCATGGGAATCCAGTGCCGGTGGTTCATTCACCGTTAGAACAGATACCGGTGAACCAATGGGTAGAGGTGTAAAAGTCATTTTGCATTTGAAGGAAGACCAAACTGAATATTTGGAAGAAAGAAGAATTAAAGAAATCGTTAAAAAGCACTCCCAATTCATTGGTTACCCTATTACATTATTCGTTGAAGGTTCT | PatchDock |
| FEN2 | 1pz4 | V11A I15N  R18L I22A  R27P E30T  M49T V54G  L105T | ATGGGTATTAGAATGTCCTTAAAATCAGACGAAGCCTTTGCCAAGAACGCCAAGTTATTAGAATCCGCCGACCCTGCCAACCCTCAAGTAACCCATGTTTACAAGTTTAGAATCACTCAAGGTGGTAAAGTTGTAAAGAATTGGGTAACTGATTTGAAAAACGGTAAATTGGTTGAATCCGATGACGCTGCAGAAGCTACTTTGACAATGGAAGATGACATTATGTTCGCAATAGGTACAGGTGCATTACCAGCCAAAGAAGCCATGGCTCAAGATAAGATGGAAGTCGACGGTCAAGTTGAATTGATCTTTTTAACCGAACCTTTCATTGCTTCTTTGAAGGGTTCA | PatchDock |
| FEN3 | 1tfj | L32A F37W  F98A F102T  L103A N122A  F156L L160W  C171T K174L  V175L L177I | ATGGAACACTTATTGAGACCATTGCCAGCCGACAAACAAATCGAAACAGGTCCATTTTTAGAAGCCGTATCCCACTTACCACCATTCTTCGATTGTGCTGGTTCCCCAGTATGGACTCCTATAAAAGCAGACATCAGTGGTAACATCACAAAGATTAAAGCTGTCTACGATACCAATCCAACTAAGTTTAGAACATTGCAAAACATCTTAGAAGTCGAAAAGGAAATGTACGGTGCAGAATGGCCTAAGGTTGGTGCTACCTTGGCATTAATGTGGTTGAAAAGAGGTTTAAGAGCAATCCAAGTTACTGCCCAATCTATTTGCGATGGTGAAAGAGACGAAAATCATCCAAACTTGATTAGAGTTGCTGCAACCAAGGCCTATGAAATGGCTTTGAAGAAATACCACGGTTGGATTGTACAAAAGATTTTTCAAGCCGCTTTGTATGCAGCCCCTTACAAGTCTGACTTGTTAAAAGCTTGGTCAAAGGGTCAAAATGTTACAGAAGAAGAAACCTTGGAATTGTTGAGAATTTTCTTAGTTAACTACACTGCCACAATCGATGTAATCTATGAAATGTACACTAGAATGAACGCAGAATTGAACTACAAGGTTGGTTCA | PatchDock |
| FEN4 | 1y9r | N34A A37F  Q40F W70A  L74S S75A  R81A Y92W  F93Y M109T  L112W L194I  C198V L216A | ATGTCCCCTGTAATGGTCTTGGAAAACATCGAACCTGAAATCGTCTACGCTGGTTATGACTCCTCAAAGCCTGATACCGCAGAAAACTTGTTGTCAACTTTAGCCAGATTGTTCGGTAAATTCATGATCCAAGTTGTAAAGTGGGCTAAGGTCTTGCCAGGTTTTAAAAATTTGCCTTTAGAAGACCAAATCACTTTGATCCAATACTCTGCTATGTGTTTGTCAGCCTTCGCTTTGTCTTGGGCATCATACAAGCATACAAACTCTCAATTCTTATGGTACGCACCAGATTTGGTTTTCAACGAAGAAAAGATGCATCAATCTGCCACCTATGAATGGTGCCAGGGTATGCACCAAATATCATTGCAATTCGTTAGATTGCAATTGACTTTCGAAGAATACACAATCATGAAGGTATTGTTGTTGTTATCCACAATCCCAAAAGACGGTTTAAAGAGTCAAGCTGCATTCGAAGAAATGAGAACCAACTACATAAAAGAATTGAGAAAGATGGTAACTAAAGCTCCTAATAACTCCGGTCAAAGTTGGCAAAGATTCTACCAATTGACAAAGTTGTTGGATTCCATGCATGACTTAGTCAGTGATATCTTGGAATTCGTTTTCTACACCTTTAGAGAATCCCACGCTTTGAAAGTAGAATTCCCTGCAATGGCCGTCGAAATAATCTCTGATCAATTGCCAAAGGTTGAATCTGGTAACGCAAAACCTTTGTACTTTCACAGAGGTTCT | PatchDock |
| FEN5 | 2i4j | F67H C70A  S74A E80D  H108F I111L  Y112I M114L  L115A S117A  L118T S127G  E128G F148I  K152S V231L  H234W | ATGGAATCCGCAGACTTGAGAGCATTAGCCAAACACTTATACGACAGTTACATTAAATCCTTCCCATTGACAAAAGCAAAAGCCAGAGCAATATTGACAGGTAAAACTACAGATAAATCCCCATTCGTCATCTATGACATGAACAGTTTGATGATGGGTGAAGATAAAATTAAGTTTAAACATATCACCCCTTTACAAAAGGAAGTTGCTATCAGAATTCACCAAGGTGCACAATTCAGAGCTGTTGAAGCAGTACAAGATATAACCGAATACGCAAAGTCTATCCCAGGTTTCGTAAATTTGGATTTGAACGACCAAGTAACTTTGTTAAAGTACGGTGTCTTCGAAATATTGATCACATTAGCTGCAGCCACCATGAATAAGGATGGTGTTTTGATTGGTGGTGGTCAAGGTTTTATGACTAGAGAATTCTTGAAGTCTTTGAGAAAGCCATTTGGTGACATTATGGAACCTTCATTTGAATTCGCTGTTAAGTTTAACGCCTTGGAATTAGATGACTCCGATTTGGCCATTTTCATAGCTGTTATTATCTTGAGTGGTGACAGACCAGGTTTGTTGAACGTAAAGCCTATCGAAGATATACAAGACAACTTGTTGCAAGCCTTGGAATTGCAATTGAAGTTGAACCATCCAGAATCTTCACAATTGTTCGCTAAGTTGTTGCAAAAGATGACTGATTTGAGACAAATCTTGACAGAATGGGTCCAATTGTTGCAAGTTATTAAAAAGACCGAAACTGATATGTCTTTGCACCCTTTGTTGCAAGAAATCTATAAGGACTTAGGTTCA | PatchDock |
| FEN6 | 2fwv | G55Y Y62G  Q66S I68V  S84D E100A  L113A H115Y  E121A F123Y  R154I Y156V  Y166F E168V  L181A | ATGGCAGCAGTAGAAAGAGCAAAAGCCACAGCAGCCAGAAACATCCCAGCCTTCGACGACTTACCAGTACCAGCCGATACAGCCAACTTAAGAGAAGGTGCCGATTTGAATAACGCCTTGTTAGCTTTGTTACCATTAGTCGGTGTTTGGAGAGGTGAGGGTGAATATAGAGGTCCTGATGGTGACGGTAGATTTGGTTCTCAAGTTGTAGTCTCACATGATGGTGGTGACTATTTGAATTGGGAATCTAGAGATTGGAGATTAACTGCAACTGGTGACTACCAAGAACCAGGTTTGAGAGCTGCAGGTTTTTGGAGATTCGTTTCCGATCCAGATGACCCTTCCGAAAGTCAAATTGAATTGTTAGCCGCTTATTCTGCAGGTTACGTAGCCTTATATTACGGTAGACCTAGAACCCAATCTTCATGGGAATTGGTTACAGATGCATTAGCCAGATCTAGATCAGGTGTATTGGTCGGTGGTGCTAAAATTTTAGTTGGTATAGTAGAAGGTGGTGACTTGGCTTTTGTTGTAGAAAGAGTCGATGCAGACGGTGGTTTGGTTCCACACGCTTCCGCAAGATTAAGTAGATTCGTTGGTGGTTCA | PatchDock |
| FEN7 | 2hwr | P21E I65L  G68L C69W  R72D S73M  A76L Q78K  E79A H107L  L114S S126G  M148Y | ATGGAATCAGCAGACTTGAGAGCATTGGCAAAACACTTGTACGACAGTTACATTAAATCTTTCCCATTGACTAAGGCTAAGGCCAGAGCAATATTGACAGGTAAAACTACAGATAAATCCCCATTCGTCATCTATGACATGAACAGTTTGATGATGGGTGAAGATAAAATTAAGTTTAAACATATCACCCCTTTACAAGAACAATCCAAGGAAGTTGCTATCAGAATTCACCAAGGTGCACAATTCAGAGCTGTTGAAGCAGTACAAGATATAACCGAATACGCAAAGTCTATCCCAGGTTTCGTAAATTTGGATTTGAACGACCAAGTAACTTTGTTAAAGTACGGTGTCTTCGAAATATTGATCACATTAGCTGCAGCCACCATGAATAAGGATGGTGTTTTGATTGGTGGTGGTCAAGGTTTTATGACTAGAGAATTCTTGAAGTCTTTGAGAAAGCCATTTGGTGACATTATGGAACCTTCATTTGAATTCGCTGTTAAGTTTAACGCCTTGGAATTAGATGACTCCGATTTGGCCATTTTCATAGCTGTTATTATCTTGAGTGGTGACAGACCAGGTTTGTTGAACGTAAAGCCTATCGAAGATATACAAGACAACTTGTTGCAAGCCTTGGAATTGCAATTGAAGTTGAACCATCCAGAATCTTCACAATTGTTCGCTAAGTTGTTGCAAAAGATGACTGATTTGAGACAAATCTTGACAGAATGGGTCCAATTGTTGCAAGTTATTAAAAAGACCGAAACTGATATGTCTTTGCACCCTTTGTTGCAAGAAATCTATAAGGACTTAGGTTCA | PatchDock |
| FEN8 | 2pir | V16T A19S  N37A G40A  Q43M L44Y  W73H M74F  M77A V78I  A80R M81A  F96W M112S  Q115L L205A  T209M | ATGTGCCAACCAATCTTTTTGAACGTATTAGAAGCCATCGAACCAGGTACAGTTTGCTCAGGTCACGACAACAACCAACCAGACTCATTCGCTGCATTGTTATCTTCATTAGCAGAATTGGCCGAAAGAATGTATGTTCATGTTGTAAAATGGGCCAAGGCTTTACCAGGTTTTAGAAATTTGCATGTTGATGACCAAATGGCAGTAATCCAATATTCTCACTTTGGTTTGGCCATTTTCAGAGCTGGTTGGAGATCTTTTACTAACGTCAACTCCAGAATGTTGTACTGGGCCCCTGATTTGGTTTTCAACGAATACAGAATGCATAAGTCCAGAAGTTACTCTTTGTGTGTAAGAATGAGACACTTGTCACAAGAATTTGGTTGGTTGCAAATAACTCCACAAGAATTCTTGTGCATGAAGGCTTTGTTATTGTTCTCCATAATCCCTGTTGATGGTTTGAAAAATCAAAAGTTTTTCGACGAATTGAGAATGAACTACATCAAGGAATTGGATAGAATCATTGCATGTAAAAGAAAGAATCCAACTTCATGCTCCAGAAGATTCTACCAATTGACAAAGTTGTTGGACTCTGTTCAACCTATAGCTAGAGAAGCACATCAATTCATGTTCGATTTGTTGATTAAGAGTCACATGGTCTCTGTTGACTTTCCAGAAATGATGGCTGAAATCATCAGTGTACAAGTCCCAAAGATCTTGTCTGGTAAAGTTAAGCCTATATACTTCCATACAGGTTCA | PatchDock |
| FEN9 | 2qg0 | N35M K42W  I75V M82A  D86W N90W  L91A S97T | ATGGTCGAAACCTTTGCCTTCCAAGCCGAAATCGCCCAATTAATGTCCTTAATCATCAACACCTTCTACTCTAACAAAGAAATCTTTTTAAGAGAATTGATATCTATGTCTTCAGATGCTTTAGACTGGATCAGATACGAATCATTGACTGATCCATCTAAGTTGGACTCTGGTAAAGAATTGCATATAAATTTGATCCCTAACAAACAAGATAGAACTTTGACAGTTGTAGACACCGGTATTGGTGCCACTAAGGCTTGGTTAATCAATTGGGCAGGTACAATTGCCAAAACCGGTACTAAGGCTTTTATGGAAGCATTGCAAGCTGGTGCAGATATCTCCATGATTGGTCAATTTGGTGTAGGTTTCTATAGTGCCTACTTAGTCGCTGAAAAAGTCACTGTTATTACAAAGCACAACGATGACGAACAATACGCATGGGAATCCAGTGCCGGTGGTTCTTTCACTGTTAGAACAGATACCGGTGAACCAATGGGTAGAGGTACAAAAGTAATATTGCATTTGAAGGAAGATCAAACCGAATATTTGGAAGAAAGAAGAATTAAAGAAATCGTTAAAAAGCACTCCCAATTCATTGGTTACCCTATAACATTATTCGTTGAAGGTTCT | PatchDock |
| FEN10 | 3cs4 | Y25F Y29G  D31S F32L  L61A L64E  V65A Y67G  S68A I99T  M103L S109G  C119W Y126L  V131L H136Y  H228K | ATGTCATTGAGACCTAAGTTATCAGAAGAACAACAAAGAATCATCGCCATATTATTGGACGCCCACCACAAGACATTTGACCCTACTGGTTCTTCATTATGTCAATTCAGACCACCTGTTAGAGTAAATGATGGTGGTGGTTCTGTAACTTTGGAATTGTCTCAATTGTCAATGTTACCTCATGCTGCAGACGAAGCATCAGGTGCCATACAAAAAGTTATCGGTTTTGCAAAGATGATTCCAGGTTTCAGAGATTTGACATCCGAAGACCAAATCGTTTTGTTAAAATCCAGTGCCACCGAAGTAATTTTGTTGAGATCTAACGAAGGTTTTACCATGGATGACATGTCATGGACTTGGGGTAACCAAGATTACAAGTTGAGAGTTTCCGACTTGACAAAGGCTGGTTACAGTTTGGAATTGATAGAACCTTTGATTAAATTCCAAGTCGGTTTGAAAAAGTTGAACTTACATGAAGAAGAACACGTTTTGTTAATGGCCATTTGTATAGTATCACCTGATAGACCAGGTGTCCAAGACGCCGCTTTGATCGAAGCTATCCAAGATAGATTGTCCAACACTTTGCAAACATACATCAGATGCAGACATCCACCTCCAGGTAGTCACTTGTTGTACGCTAAGATGATCCAAAAGTTGGCAGATTTGAGATCCTTGAACGAAGAAAAATCTAAGCAATACAGATGTTTGTCTTTCCAACCTGAATGCTCAATGAAATTGACCCCATTGGTCTTAGAAGTTTTCGGTGGTTCT | PatchDock |
| FEN11 | 3fa6 | T61W V76A  D121A M123V | ATGCCAAACTTTAGTGGTAACTGGAAAATCATCAGATCCGAAAACTTTGAAGAATTATTGAAGGTATTAGGTGTAAATGTCATGTTGAGAAAAATCGCCGTTGCTGCAGCCTCCAAACCAGCTGTAGAAATTAAGCAAGAAGGTGACACTTTCTACATCAAAGTAAGTACTACAGTCAGAACATGGGAAATTAATTTCAAAGTAGGTGAAGAATTCGAAGAACAAACTGCAGACGGTAGACCATGTAAATCTTTGGTCAAGTGGGAATCAGAAAATAAGATGGTTTGCGAACAAAAGTTGTTAAAGGGTGAAGGTCCTAAGACATCTTGGACCTTGGAATTAACCAACGATGGTGAATTAATAGCTACCGTTACTGCAGATGACGTTGTATGTACAAAGGTCTTCGTTAGAGAAGGTTCA | PatchDock |
| FEN12 | 3fmz | L35P F36Y  A57V V61Q  A71L M73F  F77M M88V  Y90G F96A  G100Y H104F  Q117A Y133S  F135L | ATGGAAAGAGATTGCAGAGTCTCATCATTCAGAGTAAAGGAAAACTTCGACAAGGCTAGATTCAGTGGTACATGGTATGCTATGGCTAAAAAGGACCCAGAAGGTCCTTATTTGCAAGATAACATCGTTGCAGAATTTTCTGTAGATGAAACCGGTCAAATGTCTGCCACTGTAAAGGGTAGACAAAGATTGTTGAACAACTGGGACGTCTGTTTGGATTTTGTTGGTACTATGACAGACACCGAAGATCCAGCCAAATTCAAGGTAAAAGGTTGGGGTGTCGCTTCAGCATTGCAAAAGTACAACGATGACTTCTGGATTGTTGATACTGACTATGATACATACGCCGTAGCTTACTCATGTAGATTGTTAAATTTGGACGGTACATGCGCAGATTCTTCATCCTTGGTCTTCTCCAGAGATCCTAACGGTTTACCACCTGAAGCTCAAAAGATTGTTAGACAAAGACAAGAAGAATTGTGTTTGGCAAGACAATACAGATTGATCGTTCATAACGGTTACTGCGGTTCT | PatchDock |
| FEN13 | 3fmz | L35P F36Y  V61Y A71L  M73F G75V  Y90G F96A  G100H H104F  Q117A Y133S | ATGGAAAGAGATTGTAGAGTCAGTTCATTCAGAGTAAAGGAAAACTTCGATAAGGCAAGATTCAGTGGTACATGGTACGCTATGGCTAAGAAGGACCCAGAAGGTCCTTATTTGCAAGATAATATCGTCGCCGAATTTTCTGTTGATGAAACCGGTCAAATGTCTGCTACTGCAAAGGGTAGATACAGATTGTTGAACAACTGGGACGTTTGTTTGGATTTTGTTGTAACTTTCACAGACACCGAAGATCCAGCAAAGTTTAAAATGAAAGGTTGGGGTGTTGCCTCAGCTTTACAAAAGCATAACGATGACTTCTGGATTGTAGATACTGACTATGATACATACGCAGTCGCCTATTCATGTAGATTGTTAAATTTGGACGGTACATGCGCTGATTCTTCATCCTTTGTTTTCTCCAGAGATCCTAACGGTTTACCACCTGAAGCTCAAAAGATTGTAAGACAAAGACAAGAAGAATTGTGTTTGGCAAGACAATACAGATTGATAGTTCACAATGGTTACTGCGGTTCT | PatchDock |
| FEN14 | 3fmz | L35P A57V  V61Y A71L  M73T G75V  Y90G F96A  G100H H104F  Q117A Y133T | ATGGAAAGAGATTGTAGAGTATCCTCATTCAGAGTAAAAGAAAACTTCGATAAGGCAAGATTCAGTGGTACATGGTATGCTATGGCTAAGAAGGACCCAGAAGGTCCATTTTTGCAAGATAATATCGTCGCAGAATTCTCCGTTGATGAAACAGGTCAAATGAGTGCCACCGTTAAGGGTAGATACAGATTGTTGAACAACTGGGACGTATGTTTGGATACAGTTGTAACTTTTACAGACACCGAAGATCCAGCCAAATTCAAGATGAAAGGTTGGGGTGTAGCTTCCGCATTACAAAAGCATAACGATGACTTTTGGATTGTAGATACTGACTATGATACATACGCCGTCGCTTACTCTTGTAGATTGTTAAATTTGGACGGTACCTGCGCAGATTCTACTTCATTTGTCTTCTCAAGAGATCCTAACGGTTTACCACCTGAAGCTCAAAAGATTGTTAGACAAAGACAAGAAGAATTGTGTTTGGCAAGACAATACAGATTGATAGTTCACAATGGTTACTGCGGTTCA | PatchDock |
| FEN15 | 1i7g | N21T M22G  C67W T70F  S71W E73A  T74L M111L  L112A S114A  V115F M116I  M121Y V123S  A124N Y125G  I130T L135V  I145G M146G  K149A H231A | ATGGAAACCGCCGATTTGAAATCATTGGCTAAAAGAATCTACGAAGCATACTTAAAGAACTTCACTGGTAACAAGGTCAAGGCAAGAGTCATATTATCCGGTAAAGCCAGTAATAACCCACCTTTTGTTATCCACGATATGGAAACATTGTGTATGGCTGAAAAGACTTTAGTAGCCAAGTTGGTCGCTAATGGTATCCAAAACAAAGAAGCTGAAGTTAGAATCTTCCATTGTTGGCAATGCTTCTGGGTCGCATTAGTTACCGAATTGACTGAATTTGCTAAGGCAATTCCAGGTTTCGCAAATTTGGATTTGAACGACCAAGTTACCTTGTTGAAGTACGGTGTATACGAAGCTATATTCGCCTTAGCTTCTGCATTCATAAATAAGGATGGTTATTTGTCAAACGGTGGTAACGGTTTTACTACAAGAGAATTCGTTAAGTCTTTGAGAAAGCCATTCTGCGATGGTGGTGAACCTGCATTTGACTTCGCCATGAAGTTTAATGCATTGGAATTGGATGACTCTGATATCTCATTGTTCGTAGCTGCAATTATATGTTGCGGTGACAGACCAGGTTTGTTGAACGTTGGTCACATCGAAAAGATGCAAGAAGGTATCGTTCATGTATTGAGATTACACTTGCAATCAAACCATCCAGATGACATTTTCTTGTTCCCTAAGTTGTTGCAAAAGATGGCTGATTTGAGACAATTGGTCACTGAAGCCGCTCAATTGGTTCAAATCATTAAAAAGACAGAATCCGATGCAGCCTTGCATCCTTTGTTGCAAGAAATCTATAGAGACATGTACGGTAGT | PatchDock |
| FEN16 | 2hb7 | Y25F Y29G  F32A V65T  S68G I99G  I102L M103L  E108D S109T  C119M V131W  H136Y L144N  E227L H228W  Y232W | ATGTCATTGAGACCTAAGTTATCCGAAGAACAACAAAGAATCATCGCCATTTTATTGGACGCACATCACAAGACATTTGACCCTACTGGTTCCGATGCTTGTCAATTCAGACCACCTGTTAGAGTAAATGACGGTGGTGGTAGTGTAACTTTAGAATTGTCCCAATTGAGTATGTTGCCTCATTTGGCAGATTTGACATCTTATGGTATCCAAAAAGTTATCGGTTTTGCCAAGATGATCCCAGGTTTCAGAGATTTGACCTCAGAAGACCAAATAGTTTTGTTGAAGTCTTCAGCCGGTGAAGTATTGTTGTTGAGATCCAACGATACTTTTACAATGGATGACATGAGTTGGACAATGGGTAACCAAGATTACAAGTACAGAGTTTCTGACTGGACCAAGGCAGGTTACTCATTAGAATTGATAGAACCTAACATCAAGTTCCAAGTCGGTTTGAAGAAATTGAACTTGCATGAAGAAGAACACGTTTTGTTGATGGCTATTTGTATCGTATCCCCTGATAGACCAGGTGTCCAAGACGCTGCATTGATCGAAGCTATCCAAGATAGATTGTCTAACACCTTGCAAACTTACATAAGATGCAGACATCCACCTCCAGGTTCACACTTATTGTACGCCAAGATGATCCAAAAGTTGGCTGATTTGAGATCTTTGAACGAATTGTGGTCAAAACAATGGAGATGTTTATCCTTTCAACCTGAATGCAGTATGAAGTTGACACCATTAGTCTTGGAAGTTTTCGGTGGTTCA | PatchDock |
| FEN17 | 1sjw | L50W V54M  F58G L64V  L82F G84S  L90T M93I  F100A G102F  Q104S F124Q  Q125E T127W  L128A L131A  W135K | ATGTCAAGACAAACCGAAATAGTAAGAAGAATGGTATCCGCTTTTAACACTGGTAGAACTGACGACGTAGACGAATACATCCACCCTGACTATTTGAATCCAGCTACCTTAGAACATGGTATACACACTGGTCCTAAAGCTTTTGCACAATGGGTTGGTTGGATGAGAGCAACAGGTTCCGAAGAAGCCAGAGTTGAAGAAGTAAGAATCGAAGAAAGAGGTCCATGGGTAAAGGCTTATTTGGTCTTTTACAGTAGACATGTCGGTAGAACTGTTGGTATTCCACCTACAGACAGAAGAGCATCTTTCGAATCAGTTCATTTGATGAGAATCGTAGATGGTAAAATCAGAGATCACAGAGACTGGCCTGATCAAGAAGGTTGGGCCAGACAAGCTGGTGACCCAAAGCCTGATGACGAAGGTTGGAGAGGTTCT | PatchDock |
| FEN18 | 3bb9 | H17M Q21T  E40H D59A  L63T L66C  I68H A90V  T101M E103Q | ATGGCCTTCATCGGTGTAGACTCCGCCGCTGGTAATGTAGTAAAACAATTCATGGCCGCCTTGACAATGGGTAACGAAGCAATAGTAAGACAATCATTGGCTGCAAATGTTCAAATCTATCATGGTGGTAAAGTAGAAAGATCTTTGACAGAATATGCAAACCATCACATGTTAGCCGCTATGGCCTACACCAAAGGTTGTACTCATACACCAAAGGAACACCAAATTACCATAACTGGTGACATCGCTATTTCTACCTCAATTTCCCATGTACAGGGTGAATACAAGGGTAAATCAATCGATTCCATGATGATGCAAACTTTGGTCTTGATTAAACAAGCAGACGGTAGATGGAAGATCACACATGTTCACTGGAGTGGTTCT | PatchDock |
| FEN19 | 3bb9 | H17W Q21T  E40Y Y51F  D59A L63A  I68F S88A | ATGGCTTTCATCGGTGTAGACAGTGCAGCCGGTAACGTCGTAAAACAATTCTGGGCAGCCTTAACAATGGGTAACGAAGCAATAGTCAGACAATCCTTAGCTGCAAATGTTCAAATCTATTACGGTGGTAAAGTAGAAAGATCATTGACCGAATTTGCCAACCATCACATGTTAGCCGCTATGGCTTATGCTAAAGGTTTGACTTTCACACCAAAGGAACATCAAATTACCATAACTGGTGACATCGCAATTTCTACTTCAATTGCTCACGCACAGGGTGAATACAAGGGTAAATCTATCGATTCAATGACAATGGAAACCTTGGTCTTGATTAAACAAGCTGACGGTAGATGGAAGATCACACATGTTCACTGGTCCGGTAGT | PatchDock |
| FEN20 | 2chc | F47A H65I  V69W H73S  S93G V95H  L106H G107A  L129F N131M  N143A L156A | ATGGGTGCAATGGGTCCTGTAGACGAACAATGGATAGAAATCTTGAGAATACAAGCCTTATGTGCCAGATACTGCTTGACAATTAACACCCAAGATGGTGAAGGTTGGGCCGGTTGTTTTACAGAAGATGGTGCTTATGAATCTGACGGTTGGGTAATCAGAGGTAGACCAGCTTTAAGAGAATTCGCAGATGCCATTGCTAGAGTTTGGAGAGGTAGATCATTGACTACAGATTTGTTATACGAAGTTGATGGTGACGTAGCTACCGGTAGATCCGCAGGTGTTCATACCTTGGCAACTGCTGCAGGTTATAAAATATTAGCCAGTGGTGAATACCAAGATAGATTGATTAAGCAAGACGGTCAATGGAGAATAGCATACAGAAGATTCAGAATGGATAGATTAGTCTCTGACCCTTCAGTTGCTGTATCTGTCGCAGATGCCGACGTTGCCGCTGTTGATGGTCACGCTTTAGCAGCCGCAAGAAGATTAGGTACACAAATGTCAGATGGTAGT | PatchDock |
| FEN21 | 2f98 | N33S G35Y  N51A V55L  T58W F59A  L65V M91G  D121M Q126T  T128I Y129K | ATGTCCGAACAAATCGCCGCCGTTAGAAGAATGGTAGAAGCCTATAATACTGGTAAAACCGACGACGTTGCCGACTACATCCACCCTGAATATATGTCTCCATACACTTTGGAATTCACTTCATTAAGAGGTCCTGAATTGTTCGCTATCGCAGTTGCCTGGTTGAAGAAATGGGCTTCCGAAGAAGCAAGAGTTGAAGAAGTAGGTATTGAAGAAAGAGCCGATTGGGTTAGAGCTAGATTGGTCTTATATGGTAGACACGTCGGTGAAGGTGTTGGTATGGCACCAACAGGTAGATTATTTTCTGGTGAACAAATCCACTTGTTGCATTTCGTAGATGGTAAAATCCATCACCATAGAATGTGGCCTGACTACACCGGTATAAAGAGACAATTGGGTGAACCATGGCCTGAAACTGAACAT | PatchDock |
| FEN22 | 3fgy | F14L F15W  W36K I67M  Y69W E119A | ATGTCCACACAAGAAAATGTTCAAATCGTAAAAGACTTATGGGCAGCAATGGGTAGAGGTGACAAGAAGGGTTTATTAGCAGTATCCGCAGAAGATATCGAAAAGATTATCCCTGGTGAATGGCCTTTGGCCGGTACCCATAGAGGTCACGCTGCATTAGCCGCTTTGTTACAAAAGGCTTCCGAAATGGTAGAAATGAGTTGGCCAGAACCACCTGAATTTGTCGCACAAGGTGAAAGAGTTTTGGTTGTAGGTTTCGCCACTGGTCGTGTTAAGTCTACTAACAGAACATTCGAAGATGACTGGGTCTTCGCTATTACAGTTAGAAAATCTAAGGTAACCTCAATCAGAGCATATATTGACACATTGGCATTAGCCAGAGCTACCAATTTTAACGCAACT | PatchDock |
| FEN23 | 3fh1 | N43L T44A  W63L I66L  F74T I88L  W90L N104A  M106V A116I  G118L | ATGATCACTTTACATCCAGACGACAGATCCGAACAAACAGCCGAAATAATGAGAAGATTCAACGACGTATTCCAATTACACGACCCTGCTGCTTTGCCAGAATTGATCGCAGAAGAATGTGTAATAGAATTGTCTGTCCCAGCACCTGATGGTGCCAGACATGCTGGTAGACAAGCTTGCGTTCAATTGTTATCAGCTTTGGCAACACAACCTGGTACTAGAACAGATTTGGAAGAAACCTTTGTTGCAGGTGACAGAGCCACTTTGAGATTAAGATATTGGATGGCCGATGGTAATTCCGTCAGAGGTGTTGCTTTAGTTAGAGTACAAGACGGTAGAATTGTTGAAATAATGGAATACGTAAAAGGT | PatchDock |
| FEN24 | 3grd | N20A A21L  H23A E36A  F57W G61K  N65I D66G  Y67A Y89L  H106A Q120L | ATGCCAAAAGCAAACTTAGAAATCATCAGATCCACTTACGAAGGTTCCGCCTCCTCCGCATTGAAAGCATTAGCAGAAGCATTATCAGAAAAAGTAGAATGGACTGAAGCAGAAGGTTTTCCATACGGTGGTACATACATCGGTGTTGAAGCCATAATGGAAAACGTATGGTCTAGAAAGAAATCTGAATGGATAGGTGCTAAAGCATCCGTCAACATGTATCATGAAGTCAGTGGTAAAGATGTTATAATCGCTGAAGGCATGTTGTCTGGTGTTTACAAAGACACCGGTAAATCATTTGAAGCCGAATTCGTTGCTGTATGGCAATTAGAAAACGGTAAAATAGTTAAATTCAAGTTGTATGTAGATTCCCACTTAGTCAGAGAAGCAATGAAGTCA | PatchDock |
| FEN25 | 3b8l | Y21A V25W  F44L L46G  I49V L51D  Q54M F63A  F64G M71L  H74A H76I  V97A G99V  G101A V109A  Y115W Y131V  M133A N145T  A146W | ATGCAATGCCCAATAGAAGATAGATTAGCCATCCAAGACTTAATGATAGCCGCAGCACACGCCTGGGACACCGTTTCAGACATCGACGCCGTTTTGGATGTTTTTACTGAAGATGCTGTATTAGACGGTTCCGGTGTCGGTGACACACCAATGGTTGGTCATGCTGGTATTAGAGAAGCAGGTACTAATGTTTTTGCAAACTTGTCTCACGCTGCAATATATTTGACAAATTTCGCAGTAACCGGTTACGAAGGTGACACAGCCTCAATGAGAGCTTATGCTATTGTCATGGCTGTTGGTAAAGATGGTAGAGCCGCTACTGTAAACGGTAGATGGTTTTTCGAAGTCAGAAGAACAGAAAAAGGTTGGAAGGCCACTAGAGTTACAGCTGATTTCTTGATGCCTTTATCTGGTACCTTGGACACTTGGAAG | PatchDock |
| FEN26 | 3grd | Y13F A17L  N20A A21L  E36M F57L  Y67W Y89M  F104A H106V  W108I F118I  Q120I | ATGCCAAAAGCCAACTTAGAAATCATTAGATCCACATTCGAAGGTTCATTGTCATCCGCATTAAAGCACTTAGCAGAAGCATTGTCAGAAAAAGTTGAATGGACTATGGCCGAAGGTTTTCCATACGGTGGTACATACATAGGTGTAGAAGCTATCATGGAAAATGTCTTGTCTAGATTAGGTTCAGAATGGAATGATTGGAAAGCATCCGTTAACATGTACCATGAAGTTAGTGGTAAAGATGTAATAATAGCCGAGGGTATGATGTCTGGTGTATACAAAGACACCGGTAAATCATTCGAAGCTGAAGCAGTTGTAGTCATTCAATTGGAAAACGGTAAAATCGTTAAAATCAAGATCTATGTCGACTCCCACTTAGTTAGAGAAGCTATGAAGTCA | PatchDock |
| FEN27 | 3gzr | Y14F N38G  V39T V40F  M42L W44L  H54L I61A  Q67V Q90M  T95L D97K  D106V N126A | ATGGGTGAAGGTACTGACGCAATACAAGCCTTAATACAAGCATTCTTTACTGCCTGGAACACAAACGCTCCTGAAAGATTCGCTGAAATTTTCTGGCCAGATGGTTCTTGGGTTGGTACTTTTGGTTTGCATTTGAGAGGTAGAGACCAAATAGTTTTTGCATTGACTGCCTTCTTAAAGACAGCTTTTAAAGATTGTAAGGTCGAATTGGTTACCATTGAAGCTAGAACTATAGCACCTGGTTCAGCCTTGGCTGTTGTAACATTAATTATGGACGCCTATGTCTTGCCAAAGGGTAGACAAATGCCTAGAGCCCACGTTAGATTAACATTGTTAGCTGTAGAAAGAGAAGGTGTCTGGAGATTCATCCATGGTCACGCTACCATTGTTAATCCAGATGCTGCAAATAACGACCCTGTATTAAGAATGAAA | PatchDock |
| FEN28 | 3h3h | W14M Y30F  M36S V57W  Y60L W61A  A64W F73L  A92G K93G  E99A A114Y  Y116T | ATGCCTATTACACAAGCCTTTGCTCAACAATTCAGTAGAGAAATGATCGACGCCTGGAACGCCCACGACTTAGACGCCATCTTATCCCACTTTGCCGATGGTTTCGAATCTTCATCCCCAATGATTGTACAAATAGCAGGTCCTAGTGGTAGATTGCGTGGTAAAGAACAATGGGGTGCTTTGGCAAGAGAATGGTTAAGAATGATCCCAGATTTGCATTTGGAATGGATTGCAACTTTAGCCGGTGTCGACTCTGTTGCTATACACTATAGAGGTGGTGGTGGTAGATTGGCCTTAGCTGTTTTTCATTTCGGTCCTGACAGAAGAGTTGTAAAGGCTTTGTACCACACAGCAGGT | PatchDock |
| FEN29 | 3hk4 | N20M S34V  E36L S45V  L51A K54L  S55M F83Q  M98I E100I  Y104F E114A  Y118V | ATGACTATCGCAGAAATCGCAAAAGACTTTACCGAATTATTGAAGCAAGGTGACATGGCTGGTGCCGCCGAAAAATACAACGCAGATGATATCGCCGTTTATTTGGCTATGGAAGGTCCAATGGCTGTTGTACATGGTAAAGAAGCTGCAAGACAATTAATGCAATGGTGGCAAGAAAATCATGAAGTCCACGGTGGTTCTGTTGAAGGTCCATACGTAAACGGTGACCAATTTGCATTGAGACAAAAATTCGACGTTACTCCTAAGGCCACAGGTGAAAGAGTCACAATTGATATAGTTGGTTTGTTTACTGTTAAAAATGGTAAAATAACTGAAGCAAGATTCTATGTT | PatchDock |
| FEN30 | 3i0y | Y15F Y16W  F19A H37V  N40P Q41G  F51A F54L  M58W Y62M  E64F L66A  Y84W V86T  E95T L97H  N125I Y127M  I133A | ATGTCCGAATCCAATAGACAAAGAGCCACTGGTTTAGTTCAAGCCTTCTGGGAAGCAGCCAATAGAGGTGACTGGGACGCAATGTTAGCATTTTTGGCTGAAGATGTCGCAGTTGACTTACCAGGTGGTCCTAGAGAAATCGGTAGAGCTGCAGCCGCTTCTTTGTTGCAAAGATGGAACGATTCAATGAGATTCCAAGCCAGAGATATTGTTGTAACAGCTAACGACGAAGGTACCAGAGTTGGTGCTGAATGGGTAACTCATGGTGTCTATCACACTACAGATACAGGTCATCCAGACGCAAATGGTCAAACCTACGTTTTGCCTGGTGGTGCCTTTTTCGATGTTAGAGACGGTCAAATCACCAGAGTAACTATCTATATGAACTTACAAGAATGGGCAGCCCAAGTTTCCAGA | PatchDock |
| FEN31 | 3i0y | Y15F Y16W  F19A H37V  N40P Q41G  F51A F54L  M58E Y62M  E64F L66A  Y84W V86T  E95T L97H  N125I Y127M  I133A | ATGAGTGAAAGTAATAGACAAAGAGCAACTGGTTTAGTACAAGCCTTCTGGGAAGCAGCAAATAGAGGTGACTGGGATGCAATGTTAGCATTTTTGGCTGAAGATGTCGCAGTTGACTTACCAGGTGGTCCTAGAGAAATCGGTAGAGCTGCAGCCGCTTCTTTGTTGCAAAGAGAAAACGATTCAATGAGATTCCAAGCCAGAGATATTGTTGTAACAGCTAACGACGAAGGTACCAGAGTTGGTGCTGAATGGGTAACTCATGGTGTCTATCACACTACAGATACAGGTCATCCAGACGCAAATGGTCAAACCTACGTTTTGCCTGGTGGTGCCTTTTTCGATGTTAGAGACGGTCAAATCACCAGAGTAACTATCTATATGAACTTACAAGAATGGGCAGCCCAAGTTTCCAGA | PatchDock |
| FEN32 | 1g69 | Q48G R50W  N83V D84G  G100I Q101L  E102S D103G  A121G H123S  E127A G140T  L141I K150V  T153A R154P  I177T S197G  I199T | ATGACCAGAATCTCCAGAGAAATGATGAAGGAATTATTGTCCGTCTATTTTATTATGGGTAGTAACAACACCAAAGCCGATCCAGTCACAGTTGTACAAAAAGCCTTAAAGGGTGGTGCTACTTTGTATGGTTTTTGGGAAAAAGGTGGTGACGCATTAACAGGTGAAGCCAGAATTAAATTTGCCGAAAAGGCTCAAGCTGCATGTAGAGAAGCTGGTGTTCCATTCATAGTCGTTGGTGACGTAGAATTGGCTTTGAATTTGAAGGCAGACGGTATACATATTATATTATCCGGTGCCAACGCTAAGGAAGTTAGAGCCGCTATTGGTGACATGATATTGGGTGTCGGTGCTTCTACCATGTCAGCAGTTAAACAAGCCGAAGAAGATGGTGCAGACTATGTTACAATAGGTCCAATCTACCCTACTGAAACAGTTAAGGACGCACCAGCCGTACAAGGTGTCTCTTTGATTGAAGCTGTTAGAAGACAAGGTATCTCAATTCCAATAGTAGGTACAGGTGGTATCACCATTGATAATGCAGCCCCTGTAATCCAAGCTGGTGCAGACGGTGTCGGTATGACTTCCGCTATTAGTCAAGCAGAAGATCCTGAATCAGCTGCAAGAAAATTCAGAGAAGAAATCCAAACCTACAAGACTGGTAGA | PatchDock |
| FEN33 | 1g69 | Q48G R50W  K52Q N83V  D84G G100I  Q101L E102S  D103G A121G  H123S E127A  G140T L141I  K150V T153A  R154P I177T  S197G I199T | ATGACAAGAATCTCCAGAGAAATGATGAAAGAATTATTATCCGTCTATTTTATTATGGGTTCCAACAACACCAAAGCCGATCCTGTCACTGTTGTACAAAAAGCCTTAAAGGGTGGTGCTACTTTGTATGGTTTTTGGGAACAAGGTGGTGACGCATTAACAGGTGAAGCCAGAATTAAATTTGCCGAAAAGGCTCAAGCTGCATGTAGAGAAGCTGGTGTTCCATTCATAGTCGTTGGTGACGTAGAATTGGCTTTGAATTTGAAGGCAGACGGTATACATATTATATTATCCGGTGCCAACGCTAAGGAAGTTAGAGCCGCTATTGGTGACATGATATTGGGTGTCGGTGCTTCTACCATGTCAGCAGTTAAACAAGCCGAAGAAGATGGTGCAGACTATGTTACAATAGGTCCAATCTACCCTACTGAAACAGTTAAGGACGCACCAGCCGTACAAGGTGTCTCTTTGATTGAAGCTGTTAGAAGACAAGGTATCTCAATTCCAATAGTAGGTACAGGTGGTATCACCATTGATAATGCAGCCCCTGTAATCCAAGCTGGTGCAGACGGTGTCGGTATGACTTCCGCTATTAGTCAAGCAGAAGATCCTGAATCAGCTGCAAGAAAATTCAGAGAAGAAATCCAAACCTACAAGACTGGTAGA | PatchDock |
| FEN34 | 2gey | L21A M50I  G53A L60A  L62A V80T  F88G M89G  E104I H116M  D118V M122L | ATGTCAATGGCCGAAAGAAAAGCCTTATGTTTAGAAATGGTCGCAGCCTGGAATAGATGGGATGCCTCTGGTATTATCAAGCACTGGAGTCCAGATATAGTTCATTATTCCGAAGACAATGAAGTCTCTTCAGCCGATATGGTTAAGTTGATAGAAGGTGCATTGAAGGCCTTTCCTGACGCTCAAGCAGAAGTTAAGAGTATAATGGCTGAAGAAGATAGAGTAGCATTGAGAATAACTACAACCGCAACTCATCAAGGTGAAGGTGGTGGTGTTCAACCAACAGGTCAAAGAGTTTCTTGGCACTTGGTAATCGAATTAAGATTCGTTGACGGTAAAGTTGTAGAAATGTGGGTCGTTATTAACTTGAGACCTTTGTTAGTAAGATTGGGTAAATTACCAGATGTCCCTAAGGTAGTCTTAGAAGCCTCAGCT | PatchDock |
| FEN35 | 3en8 | S18V E26A  C36V E43L  L54A H57K  P59L K61P  Y81F A90W  T92A S94A  Q108M | ATGAGAGAAGAAAAGATTAGAGAAGCATTGAACGCACATTGGCAAGCAGTAGCCGCTGGTGACTTCGACGCCGCACACGACATATACGATGATGACGCAATCGTAGATTATCCACAATCTGGTTTGAGAATCTTGGGTAGAATGAATTTGCAAGCTGCAAGATCAAAACATTTGGGTCCACCTGCCGGTTTTGAAGTCAGAAGAATACAAGGTGAGGGTAATTTGTGGATCACTGAATTCTCTATCTCATACAACGGTAGACCTTGGTACGCCGTTGCTATTATGGAATTCAGAAACGGTAAAGTTGTACACGAAACAATGTACTTTTCCGACCCATTCGAAGCTCCTGGTTGGAGAAGTCAATGGGTTCAACAAATTGGT | PatchDock |
| FEN36 | 3i0y | Y15F Y16W  H37V N40P  Q41G F54L  M58W Y62T  E64M L66A  Y84W V86S  D94G L97G  N125V Y127T | ATGTCCGAAAGTAATAGACAAAGAGCCACAGGTTTAGTTCAAGCATTCTGGGAAGCATTCAATAGAGGTGACTGGGACGCCATGTTAGCCTTTTTGGCCGAAGATGTCGCTGTTGACTTACCAGGTGGTCCTAGAGAAATCGGTAGAGCTGCATTCGCCTCTTTGTTACAAAGATGGAATGATTCAACAAGAATGCAAGCAAGAGATATTGTTGTAACCGCCAACGACGAAGGTACTAGAGTAGGTGCAGAATGGGTTTCCCATGGTGTATATCACACTACAGGTGAAGGTGGTCCAGATGCTAATGGTCAAACCTACGTCTTGCCTGGTGGTGCATTTTTCGATGTTAGAGACGGTCAAATTACCAGAGTAACTGTCTATACAAACTTACAAGAATGGATAGCTCAAGTTTCTAGA | PatchDock |
| FEN37 | 3i0y | Y15F Y16W  H37V N40P  Q41G F54L  M58W Y62T  E64M L66A  Y84W V86S  D94G L97G  N125V Y127D  L129N | ATGTCCGAATCCAATAGACAAAGAGCCACTGGTTTAGTTCAAGCCTTCTGGGAAGCCTTCAATAGAGGTGACTGGGACGCAATGTTAGCCTTTTTGGCCGAAGATGTCGCTGTTGACTTACCAGGTGGTCCTAGAGAAATCGGTAGAGCTGCATTCGCCTCTTTGTTACAAAGATGGAATGATTCAACTAGAATGCAAGCAAGAGATATTGTTGTAACAGCCAACGACGAAGGTACCAGAGTAGGTGCAGAATGGGTTTCCCATGGTGTATATCACACTACAGGTGAAGGTGGTCCAGATGCTAATGGTCAAACATACGTCTTGCCTGGTGGTGCATTTTTCGATGTTAGAGACGGTCAAATTACCAGAGTAACTGTCTATGACAATAACCAAGAATGGATAGCTCAAGTTTCTAGA | PatchDock |
| FEN38 | 3hk4 | A24L Y27F  N28A S34V  E36W K54V  S55W M98V  E100M G102V  Y104F E114S  F116V | ATGACTATCGCTGAAATCGCTAAGGACTTTACAGAATTATTGAAACAAGGTGACAACGCCGGTGCATTGGAAAAGTTCGCTGCTGATGACATTGCTGTATATTGGGCAATGGAAGGTCCAATGGCCGTCTCTCATGGTAAAGAAGCTTTGAGACAAGTTTGGCAATGGTGGCAAGAAAATCATGAAGTACACGGTGGTTCAGTCGAAGGTCCATACGTTAACGGTGACCAATTTGCATTGAGATTCAAATTCGACGTTACTCCTAAGGCTACAGGTGAAAGAGTCACAGTTGATATGGTTGTATTATTCACCGTTAAAAATGGTAAAATCACTGAATCCAGAGTTTATTAC | PatchDock |
| FEN39 | 3grd | Y14F N38G  V39T V40F  M42L W44L  H54L I61A  Q67M Q90T  T95L D97K  D106V N126S | ATGGGTGAAGGTACAGACGCTATACAAGCATTGATACAAGCATTCTTTACTGCCTGGAACACTAACGCCCCTGAAAGATTCGCTGAAATATTCTGGCCAGATGGTTCCTGGGTTGGTACCTTTGGTTTGCATTTGAGAGGTAGAGACCAAATAGTTTTTGCATTGACCGCCTTCTTAAAGACTGCTTTTAAAGATTGTAAGATGGAATTGGTTACAATTGAAGCTAGAACCATAGCACCTGGTTCTGCCTTGGCTGTTGTAACTTTGATAACAGACGCTTATGTTTTGCCAAAGGGTAGACAAATGCCTAGAGCCCACGTTAGATTAACTTTGTTAGCTGTCGAAAGAGAAGGTGTTTGGAGATTCATCCATGGTCACTCAACAATTGTAAATCCAGATGCTGCAAATAACGACCCTGTCTTAAGAATGAAA | PatchDock |
| FEN40 | 2rej | V11W W13S  D15V Y89F  I122S E123L  N126D D127A  L173I | ATGGAATCTTGCGGTACCGTTCGTTTCTCTGACTGGGGTTCTACCGTTATCACCGCGACCACCGCGACCGCGACCACCATCCTGGAAGCGCTGGGTTACGAAACCGACGTTAAAGTTCTGTCTGTTCCGGTTACCTACACCTCTCTGAAAAACAAAGACATCGACGTTTTCCTGGGTAACTGGATGCCGACCATGGAAGCGGACATCGCGCCGTACCGTGAAGACAAATCTGTTGAAACCGTTCGTGAAAACCTGGCGGGTGCGAAATTCACCCTGGCGACCAACGCGAAAGGTGCGGAACTGGGTATCAAAGACTTCAAAGACATCGCGGCGCACAAAGACGAACTGGACGGTAAAATCTACGGCTCTCTGCCGGGTGACGCGGGTAACCGTCTGATCATCGACATGGTTGAAAAAGGTACCTTCGACCTGAAAGGTTTCGAAGTTGTTGAATCTTCTGAACAGGGTATGCTGGCGCAGGTTGCGCGTGCGGAAAAATCTGGTGACCCGATCGTTTTCATCGGTTGGGAACCGCACCCGATGAATGCGAACTTCAAACTGACCTACCTGTCTGGTGGTGACGACGTTTTCGGTCCGAACTACGGCGGCGCGACCGTTCACACCAACGTTCGTGCGGGTTACACCACCGAATGCCCGAACGTTGACAAACTGCTGCAGAACCTGTCTTTCTCTCTGCAGATGGAAAACGAAATCATGGGTAAAATCCTGAACGACGGTGAAGACCCAGAAAAAGCGGCGGCTGCGTGGCTGAAAGACAACCCGCAGTCTATCGAACCGTGGCTGTCTGGTGTTGCGACCAAAGACGGTGGTGACGGTCTGGCGGCGGTTAAGGCGGCGCTGGGCCTG | PatchDock |
| FEN41 | 3juo | Y21M R32A  I55V H64A  S68W W77Y  F103L H117I  F119V Q129T  E131Q Q138R | ATGACCTCTGAAAACCGTGCGCAGGTTGCGGCTCGTCAGCACAACCGTAAAATCGTTGAACAGATGATGCACACCCGTGGTGAAGCGCGTCTGAAAGCGCACCTGCTGTTCACCGAAGACGGTGTTGGTGGTCTGTGGACCACCGACTCTGGTCAGCCGATCGCGGTTCGTGGTCGTGAAAAACTGGGTGAAGCGGCGGTTTGGTGGCTGCAGTGCTTCCCGGACTGGGTTTACACCGACATTCAGATCTTCGAAACCCAGGACCCGAACTGGTTCTGGGTTGAATGCCGTGGTGAAGGTGCGATCGTTCTGCCGGGTTACCCGCGTGGTCAGTACCGTAACCACTTCCTGATTTCTGTGCGTTTCGAAAACGGTCTGATCAAAGAAACCCGTCAGTTCATGAACCCGTGCGAACGTTTCCGTTCTCTGGGTATCGAAGTTCCGGAAGTTCGTCGCGATGGCCTGCCGTCC | Matcher |
| FEN42 | 7std | W18H Y22L  Y42L F45Y  V67M L68Y  H77V A119L  N123V H125A  L139A P141G  I143G F150M  F154Y R158L | ATGGAAATCACCTTCTCTGACTACCTGGGTCTGATGACCTGCGTTTACGAACACGCGGACTCTCTGGACTCTAAAGACTGGGACCGTCTGCGTAAAGTTATCGCGCCGACCCTGCGTATCGACCTGCGTTCTTACCTGGACAAACTGTGGGAAGCGATGCCGGCGGAAGAATTCGTTGGTATGGTTTCTTCTAAACAGATGTACGGTGACCCGACCCTGCGTACCCAGGTTTTCATCGGTGGTACCCGTTGGGAAAAAGTTTCTGAAGACGAAGTTATCGGTTACCACCAGCTGCGTGTTCCGCACCAGCGTTACAAAGACACCACTATGAAAGAAGTTACCATGAAAGGTCACCTGCACTCTGCGGTTCTGGCGTGGTACAAAAAAATCGACGGTGTTTGGAAATTCGCGGGTGCGAAAGGTGACGGTCGTTGGGGTGAATTCGACATGGACCGTATCTACGAAGACGGTCTGGAAACCTTCGGCGATAAG | Matcher |
| FEN43 | 2z7a | V17I I35Y  D37G T44A  I65L N68L  L70V V72G  L90V L92F  S94A Y110F  M121V | ATGACCACCCAGTCTCCGGCGCTGATCGCGTCTCAGTCTTCTTGGCGTTGCATCCAGGCTCACGACCGTGAAGGTTGGCTGGCGCTGATGGCGGACGACGTTGTTTACGAAGGTCCGATCGGTAAATCTGTTGCGAACCCGGACGGTTCTGGTATCAAAGGTAAAGAAGCGGTTGGTGCGTTCTTCGACACCCACCTGGCGGCGCTGCGTGTTACCGGTACCTGCGAAGAAACCTTCCCGTCTTCTTCTCCGGACGAAATCGCGCACATCGTTGTTTTCCACGCGGAATTCGACGGTGGTTTCACCTCTGAAGTTCGTGGTGTTTTCACCTTCCGTGTTAACAAAGCGGGTCTGATCACCAACGTTCGTGGTTACTGGAACCTGGATATGATGACCTTCGGC | Matcher |
| FEN44 | 1oh0 | I15A V18L  Y55V G58S  L59H V64T  A66V F84S  M88T W90T  C95S L97E  V99T D101I  A116V W118H  L123V | ATGCTGCCGACCGCGCAGGAAGTTCAGGGTCTGATGGCGCGTTACGCGGAACTGCTGGACGTTGGTGACATCGAAGCGATCGTTCAGATGTACGCGGACGACGCGACCGTTGAAGACCCGTTCGGTCAGCCGCCGATCCACGGTCGTGAACAGATCGCGGCGTTCGTTCGTCAGTCTCACGGTGGTGGTAAAACCCGTGTTTGCCTGACCGGTCCGGTTCGTGCGTCTCACAACGGTTGCGGTGCGATGCCGTCTCGTGTTGAAACCGTTACCAACGGTCAGCCGTCTGCGGAAGACACCATCATCGTTATGCGTTTCGACGAACACGGTCGTATCCAGACCATGCAGGTTTACCACTCTGAAGTTAACGTTTCTGTT | Matcher |
| FEN45 | 1w01 | I15A V18L  F54Y F55L  G58V L59F  V64Y A66V  F84S T88M C95I L97E V99A L101I A116V L123W  T125V | ATGCTGCCGACCGCGCAGGAAGTTCAGGGTCTGATGGCGCGTTACGCGGAACTGCTGGACGTTGGTGACATCGAAGCGATCGTTCAGATGTACGCGGACGACGCGACCGTTGAAGACCCGTTTGGTCAGCCACCAATCCACGGTCGTGAACAGATCGCGGCGTACCTGCGTCAGGTTTTCGGTGGTGGTAAATACCGTGTTTGCCTGACCGGTCCGGTTCGTGCGTCTCACAACGGTTGCGGTGCGATGCCGTCTCGTGTTGAAATGGTTTGGAACGGTCAGCCGATTGCGGAAGACGCGATCATCGTTATGCGTTTCGACGAACACGGTCGTATCCAGACCATGCAGGTTTACTGGTCTGAGGTGAACTGGTCCGTG | Matcher |
| FEN46 | 1y4c | N9H G10P  D11F K12M  E41I W59T  D62A R63M  E108V N147P  E150W G257V  M327A | ATGGAAGGTAAACTGGTTATCTGGATCCACCCGTTCATGGGTTACAACGGTCTGGCGGAAGTTGGTAAAAAATTCGAAAAAGACACCGGTATCAAAGTTACCGTTGAACACCCGGACAAACTGATCGAAAAATTCCCGCAGGTTGCGGCGACCGGTGACGGTCCGGACATCATCTTCACCGCGCACGCGATGTTCGGTGGTTACGCGCAATCTGGTCTGCTGGCGGAAATCACCCCGGACAAAGCGTTCCAGGACAAACTGTACCCGTTCACCTGGGACGCGGTTCGTTACAACGGTAAACTGATCGCGTACCCGATCGCGGTTGTTGCGCTGTCTCTGATCTACAACAAAGACCTGCTGCCGAACCCGCCGAAAACCTGGGAAGAAATCCCGGCGCTGGACAAAGAACTGAAAGCGAAAGGTAAATCTGCGCTGATGTTCCCGCTGCAGTGGCCGTACTTCACCTGGCCGCTGATCGCGGCGGACGGTGGTTACGCGTTCAAATACGAAAACGGTAAATACGACATCAAAGACGTTGGTGTTGACAACGCGGGTGCGAAAGCGGGTCTGACCTTCCTGGTTGACCTGATCAAAAATAAACACATGAACGCGGACACCGACTACTCTATCGCGGAAGCGGCTTTCAACAAAGGTGAAACCGCGATGACCATCAACGGCCCGTGGGCGTGGTCTAACATCGACACCTCTAAAGTTAACTACGGTGTTACCGTTCTGCCGACCTTCAAAGGTCAGCCGTCTAAACCGTTCGTTGTTGTTCTGTCTGCGGGTATCAACGCGGCGTCTCCGAACAAAGAACTGGCGAAAGAATTCCTGGAAAACTACCTGCTGACCGACGAAGGTCTGGAAGCGGTTAACAAAGACAAACCGCTGGGTGCGGTTGCGCTGAAATCTTACGAAGAAGAACTGGCGAAAGACCCGCGTATCGCGGCGACTATGGAGAACGCGCAGAAAGGTGAAATCGCGCCAAACATCCCGCAGATGTCTGCGTTTTGGTACGCGGTTCGTACCGCGGTTATCAACGCGGCGTCTGGTCGTCAGACCGTTGACGAAGCGCTGAAAGACGCGCAAACCAACTCCTCCTCT | Matcher |
| FEN47 | 1y4c | N9P G10P  D11F E41Y  W59M R63F  E108I N147P  E150Y P151A | GAGGGTAAGCTGGTTATCTGGATCCCGCCGTTCAAGGGTTACAACGGTCTGGCAGAGGTTGGTAAAAAGTTCGAAAAAGACACCGGTATCAAAGTCACTGTTGAACACCCGGATAAACTGTACGAGAAATTCCCGCAGGTTGCGGCGACTGGTGACGGTCCGGATATCATCTTCATGGCTCACGACTTCTTCGGTGGCTAATGCGCGCAGTCTGGTCTGCTGGCGGAAATCACCCCGGACAAAGCATTCCAAGACAAGCTGTACCCGTTCACGTGGGACGCGGTTCGTTACAATGGTAAACTCATCGCGTACCCTATCGCTGTTATCGCGCTGTCTCTGATCTACAACAAAGATCTCCTCCCGAACCCGCCGAAAACCTGGGAAGAAATCCCTGCTCTGGACAAGGAACTGAAAGCGAAAGGCAAATCTGCGCTGATGTTCCCGCTGCAATATGCGTACTTCACCTGGCCGCTGATCGCTGCTGATGGTGGTTACGCGTTCAAATATGAAAACGGTAAATACGACATCAAAGACGTTGGTGTTGACAACGCGGGTGCGAAAGCGGGCCTCACTTTCCTGGTTGATCTCATCAAAAACAAACACATGAACGCCGACACCGACTACTCTATCGCGGAAGCGGCGTTTAACAAAGGCGAAACTGCCATGACTATCAATGGTCCGTGGGCGTGGTCTAACATCGACACCTCTAAAGTGAACTACGGTGTTACTGTTCTGCCTACCTTCAAAGGTCAACCGTCTAAACCGTTCGTAGGTGTCCTCTCCGCGGGTATTAACGCAGCGTCTCCGAACAAGGAGCTCGCTAAAGAATTCCTGGAAAACTACCTCCTGACCGACGAGGGTCTGGAAGCCGTAAATAAGGACAAACCGCTGGGTGCGGTTGCCCTGAAATCTTATGAAGAAGAACTGGCGAAAGATCCGCGTATCGCCGCGACTATGGAAAATGCGCAGAAGGGTGAAATTATGCCAAACATTCCGCAAATGTCTGCGTTCTGGTATGCAGTTCGTACCGCTGTCATCAATGCAGCGAGCGGTCGCCAGACGGTCGATGAAGCGCTCAAGGACGCGCAAACTAACTCTAGCTCT | Matcher |
| FEN48 | 3ff0 | Y28F R39M  D55S I62V  H71W S75L  C78T F79H  W82L W84F  F110I Y118L  N120T F122T  N136V E138I  M140L V142W  Q145I | ATGCTGGACAACGCGATCCCGCAGGGTTTCGAAGACGCGGTTGAACTGCGTCGTAAAAACCGTGAAACCGTTGTTAAATTCATGAACACCAAAGGTCAGGACCGTCTGCGTATGCACGAACTGTTCGTTGAAGACGGTTGCGGTGGTCTGTGGACCACCTCTACCGGTTCTCCGATCGTTGTTCGTGGTAAGGACAAACTGGCGGAATGGGCGGTTTGGCTGCTCAAAACCCACCCGGACCTGGAATTCTACAACATCAAAGTTTTCGAAACCGACGACCCGAACCACTTCTGGGTTGAATGCGACGGTCACGGTAAAATCCTGATCCCGGGTTACCCGGAAGGTTACCTGGAAACCCACACCCTGCACTCTTTCGAACTGGACGACGGTAAAATCAAGCGTGTTCGTATCTTCCTGAACTGGTTCCAGATCCTGCGTGCGCTGTCTATCCCGGTTCCGCAGATCAAACGCGAAGGTATCCCA | PatchDock |
| FEN49 | 2qz3 | Q7L W9F  N35S N63W  Y65A T67A  Y69V E78A  P90W | ATGTCTACCGACTACTGGCTGAACTTCACCGACGGTGGTGGTATCGTTAACGCGGTTAACGGTTCTGGTGGTAACTACTCTGTTAACTGGTCCAACACCGGTTCTTTCGTTGTTGGTAAAGGTTGGACCACCGGTTCTCCGTTCCGTACCATCAACTACAACGCGGGTGTTTGGGCGCCGAACGGTTGGGGTGCGCTGGCGCTGGTTGGTTGGACCCGTTCTCCGCTGATCGCGTACTACGTTGTTGACTCTTGGGGTACCTACCGTTGGACCGGTACCTACAAAGGTACCGTTAAATCTGATGGTGGTACCTACGACATCTACACCACCACCCGTTACAACGCGCCGTCTATCGACGGTGACCGTACCACCTTCACCCAGTACTGGTCTGTTCGTCAGTCTAAACGTCCGACCGGTTCTAACGCTACCATCACCTTCTCTAACCACGTTAACGCGTGGAAATCTCACGGTATGAACCTGGGTTCTAACTGGGCGTACCAGGTTATGGCGACCGCGGGTTACCAGTCTTCTGGTTCTTCCAATGTGACCGTTTGG | PatchDock |
| FEN50 | 2qz3 | Q7L W9F  N35S N63W  Y65A T67A  Y69V E78A  P90W T110S  R112S Q127L  Y165F Y173H | ATGTCTACCGACTACTGGCTGAACTTCACCGACGGTGGTGGTATCGTTAACGCGGTTAACGGTTCTGGTGGTAACTACTCTGTTAACTGGTCTAACACCGGTTCTTTCGTTGTTGGTAAAGGTTGGACCACCGGTTCTCCGTTCCGTACCATCAACTACAACGCGGGTGTGTGGGCGCCGAACGGTTGGGGTGCTCTGGCGCTCGTTGGTTGGACCCGTTCTCCGCTGATCGCGTACTACGTTGTTGACTCTTGGGGTACCTACCGTTGGACCGGTACCTACAAAGGTACCGTTAAATCTGACGGTGGTACCTACGACATCTACACCTCTACCTCTTACAACGCGCCGTCTATCGACGGTGACCGTACCACCTTCACCCTGTACTGGTCTGTTCGTCAGTCTAAACGTCCGACCGGTTCTAACGCGACCATCACCTTCTCTAACCACGTTAACGCGTGGAAATCTCACGGTATGAACCTGGGTTCTAACTGGGCGTTCCAGGTTATGGCGACCGCGGGTCACCAGTCTTCTGGTTCTTCTAATGTGACCGTGTGG | PatchDock |
| FEN51 | 1opy | I16A D39A  P40W F55A  Y56I G59W  V62Y F82A  M86G L95V  V97T D99V  A114V W116A  E118S L121V | ATGAACCTGCCGACCGCGCAGGAAGTTCAGGGTCTGATGGCGCGTTACGCGGAACTGGTTGACGTTGGTGACATCGAAGCGATCGTTCAGATGTACGCGGACGACGCGACCGTTGAAGCGTGGTTCGGTCAGCCGCCGATCCACGGTCGTGAACAGATCGCGGCGGCGATCCGTCAGTGGCTGGGCGGTGGTAAATACCGTGCGTGCCTGACCGGTCCGGTTCGTGCGTCTCACAACGGTTGCGGTGCGATGCCGGCGCGTGTTGAAGGTGTTTGGAACGGTCAGCCGTGCGCGGTTGACACCATCGTTGTTATGCGTTTCGACGAACACGGTCGTATCCAGACCATGCAGGTTTACGCGTCTTCCGTTAACGTTTCTGTG | PatchDock |
| FEN52 | 2qzt | I9L R16S  V17T R26I  V28L F32I  V47N L49T  M73A L104M  V107L | ATGTCTGTTGAAACCCTGATCGAACGTATCAAAGCGTCTACCGGTGCGGTTGACCCGAACGGTCCGATCAAACTGCTGGGTGTTATCCAGCTGAACATCAAAACCGCGTCTGGTGTTGAACAGTGGATCAACGACACCAAACAGCTCAAAGTTGACCAGGGCGTTTTCGCGTCTCCGGACGTTACCGTTACCGTTGGTCTGGAAGACGCGCTGGCGATCTCTGGTAAAACCCTGACCGTTGGTGACGCGCTGAAACAGGGTAAGATCGAACTGTCTGGTGACGCGGACCTCGCGGCTAAAATGGCTGAGCTGATT | PatchDock |
| FEN53 | 1qjg | Y14F L18V  N38V Y55A  S58E V65A  Y88I T93V  P97S D99A  A114G | ATGAACACCCCGGAACACATGACCGCGGTTGTTCAGCGTTTCGTTGCGGCGGTTAACGCGGGTGACCTGGACGGTATCGTTGCGCTGTTCGCGGACGACGCGACCGTTGAAGTTCCGGTTGGTTCTGAACCGCGCTCTGGTACCGCGGCGATCCGTGAATTCGCGGCGAACGAACTGAAACTGCCGCTGGCGGCGGAACTGACCCAGGAAGTTCGTGCGGTTGCGAACGAAGCGGCTTTCGCGTTCATCGTTTCTTTCGAAATCCAGGGTCGTAAAGTTGTTGTTGCGTCTATCGCGCACTTCCGTTTCAACGGTGCGGGTAAAGTTGTTTCTATGCGTGGTCTGTTCGGCGAAAAAAATATTCATGCTGGCGCT | PatchDock |
| FEN54 | 1ogz | Y14H V15I  D38H A39T  F54L Y55F  S58E L61Y  V65A F82A  V84T F86W  Y88M P97G  D99G A114W  I121V A123S | ATGAACACCCCGGAACACATGACCGCGGTTGTTCAGCGTCACATCGCGGCGCTGAACGCGGGTGACCTGGACGGTATCGTTGCGCTGTTCGCGGACGACGCGACCGTTGAACACACCGTTGGTTCTGAACCGCGTTCTGGTACCGCGGCGATCCGTGAACTGTTCGCGAACGAACTGAAATACCCGCTGGCTGCGGAACTGACCCAGGAAGTTCGTGCGGTTGCGAACGAAGCGGCGTTCGCGGCGATCACCTCTTGGGAAATGCAGGGTCGTAAAACCGTTGTTGCGGGTATCGGTCACTTCCGTTTCAACGGTGCGGGTAAAGTTGTTTCTATGCGTTGGCTGTTCGGTGAAAAAAACGTTCACTCCGGCGCG | PatchDock |
| FEN55 | 1xc1 | Q8A H9P  E57M Q58L  S99W R101A  A141E I192S  N193M Y195F  Y196A T226V  S228G R262Y  E264G | ATGGACATCACCGTTTACAACGGTGCGCCGAAAGAAGCGGCGCAGGCGGTTGCGGACGCGTTCACCCGTGCGACCGGTATCAAAGTTAAACTGAACTCTGCGAAAGGTGACCAGCTGGCGGGTCAGATCAAAGAAGAAGGTTCTCGTTCTCCGGCGGACGTTTTCTACTCTATGCTGATCCCGGCGCTGGCGACCCTGTCTGCGGCGAACCTGCTGGAACCGCTGCCGGCGTCTACCATCAACGAAACCCGTGGTAAAGGTGTTCCGGTTGCGGCGAAAAAAGACTGGGTTGCGCTGTGGGGTGCGTCTCGTGTTGTTGTTTACGACACCCGTAAACTGTCTGAAAAAGACCTGGAAAAATCTGTTCTGAACTACGCGACCCCGAAATGGAAAAACCGTATCGGTTACGTTCCGACCTCTGGTGAATTCCTGGAACAGATCGTTGCGATCGTTAAACTGAAAGGTGAAGCGGCGGCGCTGAAATGGCTGAAAGGTCTGAAAGAATACGGTAAACCGTACGCGAAAAACTCTGTTGCGCTGCAGGCGGTTGAAAACGGTGAAATCGACGCGGCGCTGTCTATGAACTTCGCGTGGCACGCGTTTGCGCGTGAAAAAGGTGTTCAGAACGTTCACACCCGTCTGAACTTCGTTCGTCACCGTGACCCGGGTGCGCTGGTTGTTTACGGTGGTGCGGCGGTTCTGAAATCTTCTCAGAACAAAGACGAAGCGAAAAAATTCGTTGCGTTCCTGGCGGGTAAAGAAGGTCAGCGTGCGCTGACCGCGGTTTACGCTGGTTACCCGCTGAACCCGCACGTTGTTTCTACCTTCAACCTGGAACCGATCGCGAAACTGGAAGCGCCGCAGGTTTCTGCGACCACCGTTTCTGAAAAAGAACACGCGACCCGTCTGCTGGAACAAGCGGGCATGAAA | Matcher |
| FEN56 | 1e3v | V19L D39A  P40T Y56L  G59W A67V  F85Y L98A  V100H D102A  A117T W119G  E121S L124W | ATGAACCTGCCGACCGCGCAGGAAGTTCAGGGTCTGATGGCGCGTTACATCGAACTGCTGGACGTTGGTGACATCGAAGCGATCGTTCAGATGTACGCGGACGACGCGACCGTTGAAGCGACCTTCGGTCAGCCGCCGATCCACGGTCGTGAACAGATCGCGGCGTTCCTGCGTCAGTGGCTGGGTGGTGGTAAAGTTCGTGTTTGCCTGACCGGTCCGGTTCGTGCGTCTCACAACGGTTGCGGTGCGATGCCGTACCGTGTTGAAATGGTTTGGAACGGTCAGCCGTGCGCGGCGGACCACATCGCGGTTATGCGTTTCGACGAACACGGTCGTATCCAGACCATGCAGACCTACGGTTCTTCTGTTAACTGGTCTGTTCGTGAA | PatchDock |
| FEN57 | 1w00 | M12A Y15A  I16V V19S  I27L M30L  Y31F D39A  Y56L G59V  L60Y M81F  M87T L96V  V98S L100G  M113A A115E  W117H | ATGAACCTGCCGACCGCGCAGGAAGTTCAGGGTCTGGCGGCGCGTGCGGTTGAACTGTCTGACGTTGGTGACATCGAAGCGCTGGTTCAGCTGTTCGCGGACGACGCGACCGTTGAAGCGCCGTTCGGTCAGCCGCCGATCCACGGTCGTGAACAGATCGCGGCGTTCCTGCGTCAGGTTTACGGTGGTGGTAAAGTGCGTGCGTGCCTGACCGGTCCGGTTCGTGCGTCTCACAACGGTTGCGGTGCGTTCCCGTTCCGTGTTGAAACCGTTTGGAACGGTCAGCCGTGCGCGGTTGACTCTATCGGTGTTATGCGTTTCGATGAACACGGTCGTATCCAGACCGCGCAGGAATACCACTCTGAGGTGAATCTCTCTGTT | Matcher |
| FEN58 | 1sjw | M10E V11R  F14W N32E  A34W T35L  F47H L50A  V54S L90W  V91N H106A  M108V H118A  R119K D120T  F124K | ATGTCTCGTCAGACCGAAATCGTTCGTCGTGAACGTTCTGCGTGGAACACCGGTCGTACCGACGATGTTGACGAATACATCCACCCGGACTACCTGGAACCGTGGCTGCTGGAACACGGTATTCACACCGGTCCGAAAGCGCACGCGCAGGCGGTTGGTTGGTCCCGCGCGACCTTCTCTGAAGAAGCGCGTCTGGAAGAAGTTCGTATCGAAGAACGTGGTCCGTGGGTTAAGGCGTATCTCGTTCTGTACGGTCGTCACGTTGGTCGTTGGAACGGTATGCCGCCGACCGACCGTCGTTTCTCTGGTGAACAGGTTGCGCTGGTTCGTATCGTTGACGGTAAAATCCGTGACGCGAAAACCTGGCCGGACAAACAGGGTACCCTGCGTCAGCTGGGTGACCCGTGGCCGGACGACGAAGGTTGGCGC | Matcher |
| FEN59 | 1oh0 | Y14A I15V  V18A I26L  D38A Y55L  G58Q L59Y  M88T W90V  L97V V99A  D101G M114A  A116E W118T | ATGCTGCCGACCGCGCAGGAAGTTCAGGGTCTGATGGCGCGTGCGGTTGAACTGGCGGACGTTGGCGACATCGAAGCGCTGGTTCAGATGTACGCGGACGACGCGACCGTTGAAGCGCCGTTCGGTCAGCCGCCGATCCACGGTCGTGAACAGATCGCGGCGTTCCTGCGTCAGCAGTACGGTGGTGGTAAAGTTCGTGCGTGCCTGACCGGTCCGGTTCGTGCGTCTCACAACGGTTGCGGTGCGATGCCGTTCCGTGTTGAAACCGTTGTTAACGGTCAGCCGTGCGCGGTTGACGCGATCGGTGTTATGCGTTTCGACGAACACGGTCGTATCCAGACCGCGCAGGAATACACCTCTGAAGTGAACCTGTCTGTG | Matcher |
| FEN60 | 4std | W18V Y22A  S44A F45H  L46R V67Y  L98A A119T  N123A H125M  P141E I143V  F150A F154Y  R158L | ATGGAAATCACCTTCTCTGACTACCTGGGTCTGATGACCTGCGTTTACGAAGTTGCGGACTCTGCGGACTCTAAAGACTGGGACCGTCTGCGTAAAGTTATCGCGCCGACCCTGCGTATCGACTACCGTGCGCACCGTGACAAACTGTGGGAAGCGATGCCGGCGGAAGAATTCGTTGGTATGGTTTCTTCTAAACAGTACCTGGGTGACCCGACCCTGCGTACCCAGCACTTCATCGGTGGTACCCGTTGGGAAAAAGTTTCTGAAGACGAAGTTATCGGTTACCACCAGGCGCGTGTTCCGCACCAGCGCTACAAAGATACCACCATGAAGGAGGTTACCATGAAGGGTCACACCCACTCTGCGGCGCTGATGTGGTACAAAAAAATCGACGGTGTTTGGAAATTCGCGGGTCTGAAAGAAGACGTTCGTTGGGGTGAATTCGACGCGGACCGTATCTACGAAGACGGTCTGGAAACTTTCGGCGATAAG | Matcher |
| FEN61 | 3std | W18V S21A  Y22V Y42L  F45I V62A  L68Y H77V  L98V A119T  S121A N123A  L139Y P141E  I143V F150A  D151S F154Y  G157A | ATGGAAATCACCTTCTCTGACTACCTGGGTCTGATGACCTGCGTTTACGAAGTTGCGGACGCGGTTGACTCTAAAGACTGGGACCGTCTGCGTAAAGTTATCGCGCCGACCCTGCGTATCGACCTGCGTTCTATCCTGGACAAACTGTGGGAAGCGATGCCGGCGGAAGAATTCGTTGGTATGGCGTCTTCTAAACAGGTTTACGGTGACCCGACCCTGCGTACCCAGGTTTTCATCGGTGGTACCCGTTGGGAAAAAGTTTCTGAAGACGAAGTTATCGGTTACCACCAGGTTCGTGTTCCGCACCAGCGTTACAAAGACACCACCATGAAAGAAGTTACCATGAAAGGTCACACCCACGCGGCGGCGCTGCATTGGTACAAAAAAATCGACGGTGTTTGGAAATTCGCGGGTTACAAAGAAGACGTTCGTTGGGGTGAATTCGACGCGTCTCGTATCTACGAAGACGCGCGCGAAACTTTCGGC | Matcher |
| FEN62 | 3kkg | W22L F44A  H45G N60Y  L63W F64A  V72A D112S  M126A Y128H  L132A V135I | ATGGGTCAGGACCGTTCTCCGATCGAAACCCAGAACGTTGAAACCGTTCTGCGTCTGTTCGACGAAGGTCTGGGTGCGCAGGACGGTTGGCGTGACGTTTGGCGTGAAACCATGACCCCGGGTTTCCGTTCTATCGCGGGTTCTAACCAGGCGGTTGAAGGTATCGAACAGGCGATCGCGTTCTACGCGGTTTGGGCGGAAGGTTTCCCGCGTCTGGAAGCGGTTGTTGAAAACGTTACCGTTGAAGGTGACAACGTTGTTGTTCAGGCGCGTCTGACCGGTGCGCAGGACGGTCCGTTCCTGGGTGTTCCGCCGTCTGGTCAGATGGTTGACGTTCCGTCTGTTACCCTGTTCACCCTGGCGGATGGTCAGGTTATCGAAGCGCGTCACTTCACCGACGCGCTGGCGATCATGACCGCTATTTCCGCGCCGCCA | Matcher |
