# Supplementary material for: Computational design of environmental sensors for the potent opioid fentanyl
Source: eLife. 2017 Sep 19;6:e28909. doi: 10.7554/eLife.28909 (PMC5655540; doi:10.7554/eLife.28909)
Supplement: Supplementary file 3. [file elife-28909-supp3.docx]

**Supplementary Table 3 | Fen49 Mutagenic Primers**

| pETCON_5’MCS | ccatacgacgttccagactacgctctgcaggctagtggtggaggaggctc |
| --- | --- |
| pETCON_3’MCS | cactgttgttatcagatctctattacaagtcctcttcagaaataagcttttgttcgg |
| Fen49_Q7L_reverse | ccgtcggtgaagttcTgccagtagtcggtag |
| Fen49_F9W_reverse | ccaccgtcggtCCagttcagccagtagtcggtag |
| Fen49_S35N_reverse | caacctttaccaacaacgaaaTTaccggtgttggaccagttaac |
| Fen49_W63N_reverse | gccagcgcaccATTaccgttcggcgcccaaacac |
| Fen49_A65Y_reverse | aaccaaccagcgccagATAaccccaaccgttcggc |
| Fen49_A67T_reverse | caaccaaccagcgTcagcgcaccccaa |
| Fen49_V69Y_reverse | aacgggtccaaccaTAcagcgccagcgcac |
| Fen49_A78E_reverse | gtcaacaacgtagtacTcgatcagcggagaacg |
| Fen49_P90W_reverse | ctttgtaggtaccggtcGGacggtaggtaccccaag |
| Fen49_A78V | gtcaacaacgtagtacAcgatcagcggagaacg |
| Fen49_A172I | cgtaccaggttatggcgaccATAggttaccagtcttctggttc |
