# Supplementary material for: Computational design of environmental sensors for the potent opioid fentanyl
Source: eLife. 2017 Sep 19;6:e28909. doi: 10.7554/eLife.28909 (PMC5655540; doi:10.7554/eLife.28909)
Supplement: Supplementary file 4. [file elife-28909-supp4.docx]

**Supplementary Table 4 | Fen49 Site Saturation Mutagenesis (SSM) Primers**

| Fen49_1 | AGGGTCGGCTAGCCATATGNNKACCGACTACTGGCTGAACT |
| --- | --- |
| Fen49_2 | GGGTCGGCTAGCCATATGTCTNNKGACTACTGGCTGAACTTCACC |
| Fen49_3 | TCGGCTAGCCATATGTCTACCNNKTACTGGCTGAACTTCACCG |
| Fen49_4 | GGCTAGCCATATGTCTACCGACNNKTGGCTGAACTTCACCGAC |
| Fen49_5 | GCTAGCCATATGTCTACCGACTACNNKCTGAACTTCACCGACGGT |
| Fen49_6 | GCCATATGTCTACCGACTACTGGNNKAACTTCACCGACGGTGG |
| Fen49_7 | TGTCTACCGACTACTGGCTGNNKTTCACCGACGGTGGTGG |
| Fen49_8 | TCTACCGACTACTGGCTGAACNNKACCGACGGTGGTGGT |
| Fen49_9 | ACCGACTACTGGCTGAACTTCNNKGACGGTGGTGGTATCGTT |
| Fen49_10 | GACTACTGGCTGAACTTCACCNNKGGTGGTGGTATCGTTAACGC |
| Fen49_11 | CTGGCTGAACTTCACCGACNNKGGTGGTATCGTTAACGCGG |
| Fen49_12 | GCTGAACTTCACCGACGGTNNKGGTATCGTTAACGCGGTTAAC |
| Fen49_13 | AACTTCACCGACGGTGGTNNKATCGTTAACGCGGTTAACGG |
| Fen49_14 | TTCACCGACGGTGGTGGTNNKGTTAACGCGGTTAACGGTT |
| Fen49_15 | ACCGACGGTGGTGGTATCNNKAACGCGGTTAACGGTTCT |
| Fen49_16 | CGACGGTGGTGGTATCGTTNNKGCGGTTAACGGTTCTGGT |
| Fen49_17 | GACGGTGGTGGTATCGTTAACNNKGTTAACGGTTCTGGTGGTAAC |
| Fen49_18 | GTGGTGGTATCGTTAACGCGNNKAACGGTTCTGGTGGTAACTAC |
| Fen49_19 | GTGGTATCGTTAACGCGGTTNNKGGTTCTGGTGGTAACTACTCTG |
| Fen49_20 | TGGTATCGTTAACGCGGTTAACNNKTCTGGTGGTAACTACTCTGTTAAC |
| Fen49_21 | TCGTTAACGCGGTTAACGGTNNKGGTGGTAACTACTCTGTTAACTGG |
| Fen49_22 | TAACGCGGTTAACGGTTCTNNKGGTAACTACTCTGTTAACTGGTCC |
| Fen49_23 | CGCGGTTAACGGTTCTGGTNNKAACTACTCTGTTAACTGGTCCAA |
| Fen49_24 | CGGTTAACGGTTCTGGTGGTNNKTACTCTGTTAACTGGTCCAACAC |
| Fen49_25 | GGTTAACGGTTCTGGTGGTAACNNKTCTGTTAACTGGTCCAACACC |
| Fen49_26 | AACGGTTCTGGTGGTAACTACNNKGTTAACTGGTCCAACACCGG |
| Fen49_27 | ACGGTTCTGGTGGTAACTACTCTNNKAACTGGTCCAACACCGGT |
| Fen49_28 | GGTTCTGGTGGTAACTACTCTGTTNNKTGGTCCAACACCGGTTCT |
| Fen49_29 | TTCTGGTGGTAACTACTCTGTTAACNNKTCCAACACCGGTTCTTTCG |
| Fen49_30 | TGGTGGTAACTACTCTGTTAACTGGNNKAACACCGGTTCTTTCGTTGT |
| Fen49_31 | TGGTAACTACTCTGTTAACTGGTCCNNKACCGGTTCTTTCGTTGTTGG |
| Fen49_32 | AACTACTCTGTTAACTGGTCCAACNNKGGTTCTTTCGTTGTTGGTAAAGG |
| Fen49_33 | CTCTGTTAACTGGTCCAACACCNNKTCTTTCGTTGTTGGTAAAGGTTG |
| Fen49_34 | AACTGGTCCAACACCGGTNNKTTCGTTGTTGGTAAAGGTTGG |
| Fen49_35 | CTGGTCCAACACCGGTTCTNNKGTTGTTGGTAAAGGTTGGACC |
| Fen49_36 | TGGTCCAACACCGGTTCTTTCNNKGTTGGTAAAGGTTGGACCACC |
| Fen49_37 | TCCAACACCGGTTCTTTCGTTNNKGGTAAAGGTTGGACCACCG |
| Fen49_38 | AACACCGGTTCTTTCGTTGTTNNKAAAGGTTGGACCACCGGT |
| Fen49_39 | ACCGGTTCTTTCGTTGTTGGTNNKGGTTGGACCACCGGTTC |
| Fen49_40 | CCGGTTCTTTCGTTGTTGGTAAANNKTGGACCACCGGTTCTCC |
| Fen49_41 | GGTTCTTTCGTTGTTGGTAAAGGTNNKACCACCGGTTCTCCGT |
| Fen49_42 | TTTCGTTGTTGGTAAAGGTTGGNNKACCGGTTCTCCGTTCCG |
| Fen49_43 | CGTTGTTGGTAAAGGTTGGACCNNKGGTTCTCCGTTCCGTACC |
| Fen49_44 | TTGGTAAAGGTTGGACCACCNNKTCTCCGTTCCGTACCATCA |
| Fen49_45 | AAAGGTTGGACCACCGGTNNKCCGTTCCGTACCATCAACT |
| Fen49_46 | AGGTTGGACCACCGGTTCTNNKTTCCGTACCATCAACTACAACG |
| Fen49_47 | TGGACCACCGGTTCTCCGNNKCGTACCATCAACTACAACGC |
| Fen49_48 | ACCACCGGTTCTCCGTTCNNKACCATCAACTACAACGCGG |
| Fen49_49 | ACCGGTTCTCCGTTCCGTNNKATCAACTACAACGCGGGTG |
| Fen49_50 | CGGTTCTCCGTTCCGTACCNNKAACTACAACGCGGGTGTT |
| Fen49_51 | GGTTCTCCGTTCCGTACCATCNNKTACAACGCGGGTGTTTGG |
| Fen49_52 | CTCCGTTCCGTACCATCAACNNKAACGCGGGTGTTTGGG |
| Fen49_53 | TCCGTTCCGTACCATCAACTACNNKGCGGGTGTTTGGGCG |
| Fen49_54 | CGTTCCGTACCATCAACTACAACNNKGGTGTTTGGGCGCCG |
| Fen49_55 | CGTACCATCAACTACAACGCGNNKGTTTGGGCGCCGAACG |
| Fen49_56 | CCATCAACTACAACGCGGGTNNKTGGGCGCCGAACGG |
| Fen49_57 | CAACTACAACGCGGGTGTTNNKGCGCCGAACGGTTGG |
| Fen49_58 | TACAACGCGGGTGTTTGGNNKCCGAACGGTTGGGGTG |
| Fen49_59 | AACGCGGGTGTTTGGGCGNNKAACGGTTGGGGTGCG |
| Fen49_60 | GCGGGTGTTTGGGCGCCGNNKGGTTGGGGTGCGCTG |
| Fen49_61 | GGTGTTTGGGCGCCGAACNNKTGGGGTGCGCTGGC |
| Fen49_62 | GTTTGGGCGCCGAACGGTNNKGGTGCGCTGGCGC |
| Fen49_63 | TGGGCGCCGAACGGTTGGNNKGCGCTGGCGCTGG |
| Fen49_64 | GCGCCGAACGGTTGGGGTNNKCTGGCGCTGGTTGGTT |
| Fen49_65 | CCGAACGGTTGGGGTGCGNNKGCGCTGGTTGGTTGGA |
| Fen49_66 | AACGGTTGGGGTGCGCTGNNKCTGGTTGGTTGGACCCG |
| Fen49_67 | GGTTGGGGTGCGCTGGCGNNKGTTGGTTGGACCCGTTCT |
| Fen49_68 | TGGGGTGCGCTGGCGCTGNNKGGTTGGACCCGTTCTCC |
| Fen49_69 | GGTGCGCTGGCGCTGGTTNNKTGGACCCGTTCTCCGC |
| Fen49_70 | GCGCTGGCGCTGGTTGGTNNKACCCGTTCTCCGCTGA |
| Fen49_71 | CTGGCGCTGGTTGGTTGGNNKCGTTCTCCGCTGATCGC |
| Fen49_72 | GCGCTGGTTGGTTGGACCNNKTCTCCGCTGATCGCGT |
| Fen49_73 | CTGGTTGGTTGGACCCGTNNKCCGCTGATCGCGTACTAC |
| Fen49_74 | GGTTGGTTGGACCCGTTCTNNKCTGATCGCGTACTACGTTGT |
| Fen49_75 | GGTTGGACCCGTTCTCCGNNKATCGCGTACTACGTTGTTGA |
| Fen49_76 | TGGACCCGTTCTCCGCTGNNKGCGTACTACGTTGTTGACTC |
| Fen49_77 | ACCCGTTCTCCGCTGATCNNKTACTACGTTGTTGACTCTTGGG |
| Fen49_78 | CGTTCTCCGCTGATCGCGNNKTACGTTGTTGACTCTTGGGG |
| Fen49_79 | TCTCCGCTGATCGCGTACNNKGTTGTTGACTCTTGGGGTACC |
| Fen49_80 | TCCGCTGATCGCGTACTACNNKGTTGACTCTTGGGGTACCTAC |
| Fen49_81 | CGCTGATCGCGTACTACGTTNNKGACTCTTGGGGTACCTACCG |
| Fen49_82 | CTGATCGCGTACTACGTTGTTNNKTCTTGGGGTACCTACCGTT |
| Fen49_83 | TCGCGTACTACGTTGTTGACNNKTGGGGTACCTACCGTTGG |
| Fen49_84 | CGCGTACTACGTTGTTGACTCTNNKGGTACCTACCGTTGGACCG |
| Fen49_85 | CGTACTACGTTGTTGACTCTTGGNNKACCTACCGTTGGACCGG |
| Fen49_86 | ACGTTGTTGACTCTTGGGGTNNKTACCGTTGGACCGGTACC |
| Fen49_87 | GTTGTTGACTCTTGGGGTACCNNKCGTTGGACCGGTACCTAC |
| Fen49_88 | TGTTGACTCTTGGGGTACCTACNNKTGGACCGGTACCTACAAAGG |
| Fen49_89 | ACTCTTGGGGTACCTACCGTNNKACCGGTACCTACAAAGGTACC |
| Fen49_90 | TTGGGGTACCTACCGTTGGNNKGGTACCTACAAAGGTACCGTT |
| Fen49_91 | GGGGTACCTACCGTTGGACCNNKACCTACAAAGGTACCGTTAAATCT |
| Fen49_92 | ACCTACCGTTGGACCGGTNNKTACAAAGGTACCGTTAAATCTGATG |
| Fen49_93 | TACCGTTGGACCGGTACCNNKAAAGGTACCGTTAAATCTGATGGT |
| Fen49_94 | CCGTTGGACCGGTACCTACNNKGGTACCGTTAAATCTGATGGTG |
| Fen49_95 | CGTTGGACCGGTACCTACAAANNKACCGTTAAATCTGATGGTGGT |
| Fen49_96 | TGGACCGGTACCTACAAAGGTNNKGTTAAATCTGATGGTGGTACCTACG |
| Fen49_97 | ACCGGTACCTACAAAGGTACCNNKAAATCTGATGGTGGTACCTACG |
| Fen49_98 | CGGTACCTACAAAGGTACCGTTNNKTCTGATGGTGGTACCTACGA |
| Fen49_99 | CGGTACCTACAAAGGTACCGTTAAANNKGATGGTGGTACCTACGACATC |
| Fen49_100 | TACCTACAAAGGTACCGTTAAATCTNNKGGTGGTACCTACGACATCTAC |
| Fen49_101 | CCTACAAAGGTACCGTTAAATCTGATNNKGGTACCTACGACATCTACACC |
| Fen49_102 | AAAGGTACCGTTAAATCTGATGGTNNKACCTACGACATCTACACCACC |
| Fen49_103 | TACCGTTAAATCTGATGGTGGTNNKTACGACATCTACACCACCACC |
| Fen49_104 | CCGTTAAATCTGATGGTGGTACCNNKGACATCTACACCACCACCC |
| Fen49_105 | CCGTTAAATCTGATGGTGGTACCTACNNKATCTACACCACCACCCGT |
| Fen49_106 | TCTGATGGTGGTACCTACGACNNKTACACCACCACCCGTTACA |
| Fen49_107 | TGATGGTGGTACCTACGACATCNNKACCACCACCCGTTACAAC |
| Fen49_108 | TGGTGGTACCTACGACATCTACNNKACCACCCGTTACAACGC |
| Fen49_109 | TGGTACCTACGACATCTACACCNNKACCCGTTACAACGCGC |
| Fen49_110 | ACCTACGACATCTACACCACCNNKCGTTACAACGCGCCGT |
| Fen49_111 | ACGACATCTACACCACCACCNNKTACAACGCGCCGTCTATC |
| Fen49_112 | ATCTACACCACCACCCGTNNKAACGCGCCGTCTATCG |
| Fen49_113 | TCTACACCACCACCCGTTACNNKGCGCCGTCTATCGACG |
| Fen49_114 | CACCACCACCCGTTACAACNNKCCGTCTATCGACGGTGAC |
| Fen49_115 | ACCACCCGTTACAACGCGNNKTCTATCGACGGTGACCGT |
| Fen49_116 | ACCCGTTACAACGCGCCGNNKATCGACGGTGACCGTACC |
| Fen49_117 | CGTTACAACGCGCCGTCTNNKGACGGTGACCGTACCAC |
| Fen49_118 | TACAACGCGCCGTCTATCNNKGGTGACCGTACCACCTTC |
| Fen49_119 | AACGCGCCGTCTATCGACNNKGACCGTACCACCTTCACC |
| Fen49_120 | GCGCCGTCTATCGACGGTNNKCGTACCACCTTCACCCAG |
| Fen49_121 | GCCGTCTATCGACGGTGACNNKACCACCTTCACCCAGTACT |
| Fen49_122 | GTCTATCGACGGTGACCGTNNKACCTTCACCCAGTACTGGT |
| Fen49_123 | ATCGACGGTGACCGTACCNNKTTCACCCAGTACTGGTCTGT |
| Fen49_124 | GACGGTGACCGTACCACCNNKACCCAGTACTGGTCTGTTCG |
| Fen49_125 | CGGTGACCGTACCACCTTCNNKCAGTACTGGTCTGTTCGTCA |
| Fen49_126 | TGACCGTACCACCTTCACCNNKTACTGGTCTGTTCGTCAGTCT |
| Fen49_127 | CCGTACCACCTTCACCCAGNNKTGGTCTGTTCGTCAGTCTAAAC |
| Fen49_128 | CGTACCACCTTCACCCAGTACNNKTCTGTTCGTCAGTCTAAACGT |
| Fen49_129 | CCACCTTCACCCAGTACTGGNNKGTTCGTCAGTCTAAACGTCCG |
| Fen49_130 | ACCTTCACCCAGTACTGGTCTNNKCGTCAGTCTAAACGTCCGA |
| Fen49_131 | TTCACCCAGTACTGGTCTGTTNNKCAGTCTAAACGTCCGACCG |
| Fen49_132 | ACCCAGTACTGGTCTGTTCGTNNKTCTAAACGTCCGACCGGT |
| Fen49_133 | CCAGTACTGGTCTGTTCGTCAGNNKAAACGTCCGACCGGTTC |
| Fen49_134 | TACTGGTCTGTTCGTCAGTCTNNKCGTCCGACCGGTTCTAAC |
| Fen49_135 | ACTGGTCTGTTCGTCAGTCTAAANNKCCGACCGGTTCTAACGC |
| Fen49_136 | GTCTGTTCGTCAGTCTAAACGTNNKACCGGTTCTAACGCTACCA |
| Fen49_137 | TTCGTCAGTCTAAACGTCCGNNKGGTTCTAACGCTACCATCACC |
| Fen49_138 | CGTCAGTCTAAACGTCCGACCNNKTCTAACGCTACCATCACCTTC |
| Fen49_139 | GTCTAAACGTCCGACCGGTNNKAACGCTACCATCACCTTCTC |
| Fen49_140 | AAACGTCCGACCGGTTCTNNKGCTACCATCACCTTCTCTAACC |
| Fen49_141 | ACGTCCGACCGGTTCTAACNNKACCATCACCTTCTCTAACCAC |
| Fen49_142 | CCGACCGGTTCTAACGCTNNKATCACCTTCTCTAACCACGTTAA |
| Fen49_143 | CGACCGGTTCTAACGCTACCNNKACCTTCTCTAACCACGTTAACG |
| Fen49_144 | ACCGGTTCTAACGCTACCATCNNKTTCTCTAACCACGTTAACGCG |
| Fen49_145 | CGGTTCTAACGCTACCATCACCNNKTCTAACCACGTTAACGCGT |
| Fen49_146 | TTCTAACGCTACCATCACCTTCNNKAACCACGTTAACGCGTGG |
| Fen49_147 | AACGCTACCATCACCTTCTCTNNKCACGTTAACGCGTGGAAAT |
| Fen49_148 | ACGCTACCATCACCTTCTCTAACNNKGTTAACGCGTGGAAATCTCAC |
| Fen49_149 | TACCATCACCTTCTCTAACCACNNKAACGCGTGGAAATCTCACG |
| Fen49_150 | CCATCACCTTCTCTAACCACGTTNNKGCGTGGAAATCTCACGGT |
| Fen49_151 | TCACCTTCTCTAACCACGTTAACNNKTGGAAATCTCACGGTATGAACC |
| Fen49_152 | TCTCTAACCACGTTAACGCGNNKAAATCTCACGGTATGAACCTGG |
| Fen49_153 | AACCACGTTAACGCGTGGNNKTCTCACGGTATGAACCTGGG |
| Fen49_154 | ACCACGTTAACGCGTGGAAANNKCACGGTATGAACCTGGGTT |
| Fen49_155 | CACGTTAACGCGTGGAAATCTNNKGGTATGAACCTGGGTTCTAACT |
| Fen49_156 | CGTTAACGCGTGGAAATCTCACNNKATGAACCTGGGTTCTAACTGG |
| Fen49_157 | CGCGTGGAAATCTCACGGTNNKAACCTGGGTTCTAACTGGGC |
| Fen49_158 | CGCGTGGAAATCTCACGGTATGNNKCTGGGTTCTAACTGGGCG |
| Fen49_159 | CGTGGAAATCTCACGGTATGAACNNKGGTTCTAACTGGGCGTACC |
| Fen49_160 | GGAAATCTCACGGTATGAACCTGNNKTCTAACTGGGCGTACCAGG |
| Fen49_161 | CTCACGGTATGAACCTGGGTNNKAACTGGGCGTACCAGGT |
| Fen49_162 | CACGGTATGAACCTGGGTTCTNNKTGGGCGTACCAGGTTATGG |
| Fen49_163 | ACGGTATGAACCTGGGTTCTAACNNKGCGTACCAGGTTATGGCG |
| Fen49_164 | TATGAACCTGGGTTCTAACTGGNNKTACCAGGTTATGGCGACCG |
| Fen49_165 | CCTGGGTTCTAACTGGGCGNNKCAGGTTATGGCGACCGC |
| Fen49_166 | TGGGTTCTAACTGGGCGTACNNKGTTATGGCGACCGCGG |
| Fen49_167 | GGTTCTAACTGGGCGTACCAGNNKATGGCGACCGCGGG |
| Fen49_168 | AACTGGGCGTACCAGGTTNNKGCGACCGCGGGTTAC |
| Fen49_169 | ACTGGGCGTACCAGGTTATGNNKACCGCGGGTTACCAGT |
| Fen49_170 | GGCGTACCAGGTTATGGCGNNKGCGGGTTACCAGTCTTCTG |
| Fen49_171 | CGTACCAGGTTATGGCGACCNNKGGTTACCAGTCTTCTGGTTCT |
| Fen49_172 | CAGGTTATGGCGACCGCGNNKTACCAGTCTTCTGGTTCTTCCA |
| Fen49_173 | GTTATGGCGACCGCGGGTNNKCAGTCTTCTGGTTCTTCCAATG |
| Fen49_174 | ATGGCGACCGCGGGTTACNNKTCTTCTGGTTCTTCCAATGTGA |
| Fen49_175 | GCGACCGCGGGTTACCAGNNKTCTGGTTCTTCCAATGTGACC |
| Fen49_176 | ACCGCGGGTTACCAGTCTNNKGGTTCTTCCAATGTGACCGT |
| Fen49_177 | CCGCGGGTTACCAGTCTTCTNNKTCTTCCAATGTGACCGTTTGG |
| Fen49_178 | GCGGGTTACCAGTCTTCTGGTNNKTCCAATGTGACCGTTTGGC |
| Fen49_179 | GGGTTACCAGTCTTCTGGTTCTNNKAATGTGACCGTTTGGCTCG |
| Fen49_180 | GTTACCAGTCTTCTGGTTCTTCCNNKGTGACCGTTTGGCTCGA |
| Fen49_181 | ACCAGTCTTCTGGTTCTTCCAATNNKACCGTTTGGCTCGAGGG |
| Fen49_182 | AGTCTTCTGGTTCTTCCAATGTGNNKGTTTGGCTCGAGGGAGG |
| Fen49_183 | TTCTGGTTCTTCCAATGTGACCNNKTGGCTCGAGGGAGGC |
| Fen49_184 | TGGTTCTTCCAATGTGACCGTTNNKCTCGAGGGAGGCGGAT |

| Fen49_1R | CATATGGCTAGCCGACCCT |
| --- | --- |
| Fen49_2R | AGACATATGGCTAGCCGACCC |
| Fen49_3R | GGTAGACATATGGCTAGCCGA |
| Fen49_4R | GTCGGTAGACATATGGCTAGCC |
| Fen49_5R | GTAGTCGGTAGACATATGGCTAGC |
| Fen49_6R | CCAGTAGTCGGTAGACATATGGC |
| Fen49_7R | CAGCCAGTAGTCGGTAGACA |
| Fen49_8R | GTTCAGCCAGTAGTCGGTAGA |
| Fen49_9R | GAAGTTCAGCCAGTAGTCGGT |
| Fen49_10R | GGTGAAGTTCAGCCAGTAGTC |
| Fen49_11R | GTCGGTGAAGTTCAGCCAG |
| Fen49_12R | ACCGTCGGTGAAGTTCAGC |
| Fen49_13R | ACCACCGTCGGTGAAGTT |
| Fen49_14R | ACCACCACCGTCGGTGAA |
| Fen49_15R | GATACCACCACCGTCGGT |
| Fen49_16R | AACGATACCACCACCGTCG |
| Fen49_17R | GTTAACGATACCACCACCGTC |
| Fen49_18R | CGCGTTAACGATACCACCAC |
| Fen49_19R | AACCGCGTTAACGATACCAC |
| Fen49_20R | GTTAACCGCGTTAACGATACCA |
| Fen49_21R | ACCGTTAACCGCGTTAACGA |
| Fen49_22R | AGAACCGTTAACCGCGTTA |
| Fen49_23R | ACCAGAACCGTTAACCGCG |
| Fen49_24R | ACCACCAGAACCGTTAACCG |
| Fen49_25R | GTTACCACCAGAACCGTTAACC |
| Fen49_26R | GTAGTTACCACCAGAACCGTT |
| Fen49_27R | AGAGTAGTTACCACCAGAACCGT |
| Fen49_28R | AACAGAGTAGTTACCACCAGAACC |
| Fen49_29R | GTTAACAGAGTAGTTACCACCAGAA |
| Fen49_30R | CCAGTTAACAGAGTAGTTACCACCA |
| Fen49_31R | GGACCAGTTAACAGAGTAGTTACCA |
| Fen49_32R | GTTGGACCAGTTAACAGAGTAGTT |
| Fen49_33R | GGTGTTGGACCAGTTAACAGAG |
| Fen49_34R | ACCGGTGTTGGACCAGTT |
| Fen49_35R | AGAACCGGTGTTGGACCAG |
| Fen49_36R | GAAAGAACCGGTGTTGGACCA |
| Fen49_37R | AACGAAAGAACCGGTGTTGGA |
| Fen49_38R | AACAACGAAAGAACCGGTGTT |
| Fen49_39R | ACCAACAACGAAAGAACCGGT |
| Fen49_40R | TTTACCAACAACGAAAGAACCGG |
| Fen49_41R | ACCTTTACCAACAACGAAAGAACC |
| Fen49_42R | CCAACCTTTACCAACAACGAAA |
| Fen49_43R | GGTCCAACCTTTACCAACAACG |
| Fen49_44R | GGTGGTCCAACCTTTACCAA |
| Fen49_45R | ACCGGTGGTCCAACCTTT |
| Fen49_46R | AGAACCGGTGGTCCAACCT |
| Fen49_47R | CGGAGAACCGGTGGTCCA |
| Fen49_48R | GAACGGAGAACCGGTGGT |
| Fen49_49R | ACGGAACGGAGAACCGGT |
| Fen49_50R | GGTACGGAACGGAGAACCG |
| Fen49_51R | GATGGTACGGAACGGAGAACC |
| Fen49_52R | GTTGATGGTACGGAACGGAG |
| Fen49_53R | GTAGTTGATGGTACGGAACGGA |
| Fen49_54R | GTTGTAGTTGATGGTACGGAACG |
| Fen49_55R | CGCGTTGTAGTTGATGGTACG |
| Fen49_56R | ACCCGCGTTGTAGTTGATGG |
| Fen49_57R | AACACCCGCGTTGTAGTTG |
| Fen49_58R | CCAAACACCCGCGTTGTA |
| Fen49_59R | CGCCCAAACACCCGCGTT |
| Fen49_60R | CGGCGCCCAAACACCCGC |
| Fen49_61R | GTTCGGCGCCCAAACACC |
| Fen49_62R | ACCGTTCGGCGCCCAAAC |
| Fen49_63R | CCAACCGTTCGGCGCCCA |
| Fen49_64R | ACCCCAACCGTTCGGCGC |
| Fen49_65R | CGCACCCCAACCGTTCGG |
| Fen49_66R | CAGCGCACCCCAACCGTT |
| Fen49_67R | CGCCAGCGCACCCCAACC |
| Fen49_68R | CAGCGCCAGCGCACCCCA |
| Fen49_69R | AACCAGCGCCAGCGCACC |
| Fen49_70R | ACCAACCAGCGCCAGCGC |
| Fen49_71R | CCAACCAACCAGCGCCAG |
| Fen49_72R | GGTCCAACCAACCAGCGC |
| Fen49_73R | ACGGGTCCAACCAACCAG |
| Fen49_74R | AGAACGGGTCCAACCAACC |
| Fen49_75R | CGGAGAACGGGTCCAACC |
| Fen49_76R | CAGCGGAGAACGGGTCCA |
| Fen49_77R | GATCAGCGGAGAACGGGT |
| Fen49_78R | CGCGATCAGCGGAGAACG |
| Fen49_79R | GTACGCGATCAGCGGAGA |
| Fen49_80R | GTAGTACGCGATCAGCGGA |
| Fen49_81R | AACGTAGTACGCGATCAGCG |
| Fen49_82R | AACAACGTAGTACGCGATCAG |
| Fen49_83R | GTCAACAACGTAGTACGCGA |
| Fen49_84R | AGAGTCAACAACGTAGTACGCG |
| Fen49_85R | CCAAGAGTCAACAACGTAGTACG |
| Fen49_86R | ACCCCAAGAGTCAACAACGT |
| Fen49_87R | GGTACCCCAAGAGTCAACAAC |
| Fen49_88R | GTAGGTACCCCAAGAGTCAACA |
| Fen49_89R | ACGGTAGGTACCCCAAGAGT |
| Fen49_90R | CCAACGGTAGGTACCCCAA |
| Fen49_91R | GGTCCAACGGTAGGTACCCC |
| Fen49_92R | ACCGGTCCAACGGTAGGT |
| Fen49_93R | GGTACCGGTCCAACGGTA |
| Fen49_94R | GTAGGTACCGGTCCAACGG |
| Fen49_95R | TTTGTAGGTACCGGTCCAACG |
| Fen49_96R | ACCTTTGTAGGTACCGGTCCA |
| Fen49_97R | GGTACCTTTGTAGGTACCGGT |
| Fen49_98R | AACGGTACCTTTGTAGGTACCG |
| Fen49_99R | TTTAACGGTACCTTTGTAGGTACCG |
| Fen49_100R | AGATTTAACGGTACCTTTGTAGGTA |
| Fen49_101R | ATCAGATTTAACGGTACCTTTGTAGG |
| Fen49_102R | ACCATCAGATTTAACGGTACCTTT |
| Fen49_103R | ACCACCATCAGATTTAACGGTA |
| Fen49_104R | GGTACCACCATCAGATTTAACGG |
| Fen49_105R | GTAGGTACCACCATCAGATTTAACGG |
| Fen49_106R | GTCGTAGGTACCACCATCAGA |
| Fen49_107R | GATGTCGTAGGTACCACCATCA |
| Fen49_108R | GTAGATGTCGTAGGTACCACCA |
| Fen49_109R | GGTGTAGATGTCGTAGGTACCA |
| Fen49_110R | GGTGGTGTAGATGTCGTAGGT |
| Fen49_111R | GGTGGTGGTGTAGATGTCGT |
| Fen49_112R | ACGGGTGGTGGTGTAGAT |
| Fen49_113R | GTAACGGGTGGTGGTGTAGA |
| Fen49_114R | GTTGTAACGGGTGGTGGTG |
| Fen49_115R | CGCGTTGTAACGGGTGGT |
| Fen49_116R | CGGCGCGTTGTAACGGGT |
| Fen49_117R | AGACGGCGCGTTGTAACG |
| Fen49_118R | GATAGACGGCGCGTTGTA |
| Fen49_119R | GTCGATAGACGGCGCGTT |
| Fen49_120R | ACCGTCGATAGACGGCGC |
| Fen49_121R | GTCACCGTCGATAGACGGC |
| Fen49_122R | ACGGTCACCGTCGATAGAC |
| Fen49_123R | GGTACGGTCACCGTCGAT |
| Fen49_124R | GGTGGTACGGTCACCGTC |
| Fen49_125R | GAAGGTGGTACGGTCACCG |
| Fen49_126R | GGTGAAGGTGGTACGGTCA |
| Fen49_127R | CTGGGTGAAGGTGGTACGG |
| Fen49_128R | GTACTGGGTGAAGGTGGTACG |
| Fen49_129R | CCAGTACTGGGTGAAGGTGG |
| Fen49_130R | AGACCAGTACTGGGTGAAGGT |
| Fen49_131R | AACAGACCAGTACTGGGTGAA |
| Fen49_132R | ACGAACAGACCAGTACTGGGT |
| Fen49_133R | CTGACGAACAGACCAGTACTGG |
| Fen49_134R | AGACTGACGAACAGACCAGTA |
| Fen49_135R | TTTAGACTGACGAACAGACCAGT |
| Fen49_136R | ACGTTTAGACTGACGAACAGAC |
| Fen49_137R | CGGACGTTTAGACTGACGAA |
| Fen49_138R | GGTCGGACGTTTAGACTGACG |
| Fen49_139R | ACCGGTCGGACGTTTAGAC |
| Fen49_140R | AGAACCGGTCGGACGTTT |
| Fen49_141R | GTTAGAACCGGTCGGACGT |
| Fen49_142R | AGCGTTAGAACCGGTCGG |
| Fen49_143R | GGTAGCGTTAGAACCGGTCG |
| Fen49_144R | GATGGTAGCGTTAGAACCGGT |
| Fen49_145R | GGTGATGGTAGCGTTAGAACCG |
| Fen49_146R | GAAGGTGATGGTAGCGTTAGAA |
| Fen49_147R | AGAGAAGGTGATGGTAGCGTT |
| Fen49_148R | GTTAGAGAAGGTGATGGTAGCGT |
| Fen49_149R | GTGGTTAGAGAAGGTGATGGTA |
| Fen49_150R | AACGTGGTTAGAGAAGGTGATGG |
| Fen49_151R | GTTAACGTGGTTAGAGAAGGTGA |
| Fen49_152R | CGCGTTAACGTGGTTAGAGA |
| Fen49_153R | CCACGCGTTAACGTGGTT |
| Fen49_154R | TTTCCACGCGTTAACGTGGT |
| Fen49_155R | AGATTTCCACGCGTTAACGTG |
| Fen49_156R | GTGAGATTTCCACGCGTTAACG |
| Fen49_157R | ACCGTGAGATTTCCACGCG |
| Fen49_158R | CATACCGTGAGATTTCCACGCG |
| Fen49_159R | GTTCATACCGTGAGATTTCCACG |
| Fen49_160R | CAGGTTCATACCGTGAGATTTCC |
| Fen49_161R | ACCCAGGTTCATACCGTGAG |
| Fen49_162R | AGAACCCAGGTTCATACCGTG |
| Fen49_163R | GTTAGAACCCAGGTTCATACCGT |
| Fen49_164R | CCAGTTAGAACCCAGGTTCATA |
| Fen49_165R | CGCCCAGTTAGAACCCAGG |
| Fen49_166R | GTACGCCCAGTTAGAACCCA |
| Fen49_167R | CTGGTACGCCCAGTTAGAACC |
| Fen49_168R | AACCTGGTACGCCCAGTT |
| Fen49_169R | CATAACCTGGTACGCCCAGT |
| Fen49_170R | CGCCATAACCTGGTACGCC |
| Fen49_171R | GGTCGCCATAACCTGGTACG |
| Fen49_172R | CGCGGTCGCCATAACCTG |
| Fen49_173R | ACCCGCGGTCGCCATAAC |
| Fen49_174R | GTAACCCGCGGTCGCCAT |
| Fen49_175R | CTGGTAACCCGCGGTCGC |
| Fen49_176R | AGACTGGTAACCCGCGGT |
| Fen49_177R | AGAAGACTGGTAACCCGCGG |
| Fen49_178R | ACCAGAAGACTGGTAACCCGC |
| Fen49_179R | AGAACCAGAAGACTGGTAACCC |
| Fen49_180R | GGAAGAACCAGAAGACTGGTAAC |
| Fen49_181R | ATTGGAAGAACCAGAAGACTGGT |
| Fen49_182R | CACATTGGAAGAACCAGAAGACT |
| Fen49_183R | GGTCACATTGGAAGAACCAGAA |
| Fen49_184R | AACGGTCACATTGGAAGAACCA |
