# Supplementary material for: Computational design of environmental sensors for the potent opioid fentanyl
Source: eLife. 2017 Sep 19;6:e28909. doi: 10.7554/eLife.28909 (PMC5655540; doi:10.7554/eLife.28909)
Supplement: Supplementary file 5. [file elife-28909-supp5.docx]

**Supplementary Table 5 | Next-generation sequencing statistics for Fen49 Library Sorts**

| **ID** | **Part** | **Library** | **Forward Reads** | **Reverse Reads** | **Pair Reads Successfully Assembled** | **Pair Reads Failed to Assemble** |
| --- | --- | --- | --- | --- | --- | --- |
| MB02 | 5-Prime | Naïve | 437,636 | 446,975 | 369,633 | 65,256 |
| MB04 | 5-Prime | Sort 1 | 510,079 | 514,110 | 279,051 | 226,532 |
| MB06 | 5-Prime | Sort 2 | 739,360 | 753,134 | 620,714 | 113,726 |
| MB07 | 5-Prime | Sort 3 | 399,645 | 403,443 | 335,594 | 61,454 |
| MB08 | 5-Prime | Sort 4 | 699,005 | 705,593 | 595,930 | 98,653 |
| MB10 | 3-Prime | Naïve | 359,375 | 366,995 | 297,690 | 59,402 |
| MB12 | 3-Prime | Sort 1 | 548,621 | 557,135 | 419,992 | 124,840 |
| MB14 | 3-Prime | Sort 2 | 361,804 | 368,395 | 285,129 | 74,257 |
| MB15 | 3-Prime | Sort 3 | 359,581 | 365,407 | 295,149 | 62,190 |
| MB16 | 3-Prime | Sort 4 | 602,414 | 612,745 | 493,991 | 104,486 |
|  |  | **Total** | 5,017,520 | 5,093,932 | 3,992,873 | 990,796 |
