# Supplementary material for: Computational design of environmental sensors for the potent opioid fentanyl
Source: eLife. 2017 Sep 19;6:e28909. doi: 10.7554/eLife.28909 (PMC5655540; doi:10.7554/eLife.28909)
Supplement: Supplementary file 6. [file elife-28909-supp6.docx]

**Supplementary Table 6 | Crystallographic Data Collection and Refinement Statistics**

|  | Fen49 | Fen49*_Apo | Fen49*_Complex |
| --- | --- | --- | --- |
| Wavelength | 0.75141 | 0.976246 | 0.999878 |
| Resolution range | 44.33 - 1.0  (1.022 - 1.00) | 43.86 - 1.788  (1.852 - 1.788) | 51.87 - 1.67  (1.73 - 1.67) |
| Space group | P 1 21 1 | P 21 21 21 | P 21 21 21 |
| Unit cell (Å, °) | 40.38 79.568 54.459 90 101.381 90 | 54.76 73.259 137.558 90 90 90 | 56.07 73.25 136.64 90 90 90 |
| Total reflections | 1,474,174 | 1,351,234 | 278,335 |
| Unique reflections | 181,340 (8,991) | 52,294 (4,773) | 65,282 (6,499) |
| Multiplicity | 4.5 (3.7) | 7.8 (5.9) | 4.3(4.4) |
| Completeness (%) | 100 (99.9) | 98.5 (92.8) | 98.9(99.9) |
| Mean I/sigma(I) | 21.0 (0.89) | 15.89 (1.25) | 14.2(2.1) |
| Wilson B-factor | 8.14 | 18.55 | 15.3 |
| R-merge | 0.058 (0.973) | 0.110 (0.778) | 0.079(0.69) |
| R-meas | 0.065 (1.137) | 0.118 (0.837) | 0.10(0.89) |
| R-pim | 0.030 (0.578) | 0.044 (0.461) | 0.048(0.40) |
| CC1/2 | 0.557 | 0.573 | 0.69 |
| Reflections used in refinement | 181,281 (11,115) | 52,282 (4,768) | 65,282 (6,491) |
| Reflections used for R-free | 2,254 (140) | 2,659 (248) | 3,321 (337) |
| R-work | 0.1080 (0.2604) | 0.2058 (0.3106) | 0.1662 (0.2486) |
| R-free | 0.1215 (0.2500) | 0.2258 (0.2765) | 0.2025 (0.2948) |
| Number of non-hydrogen atoms | 3,863 | 4,723 | 5,280 |
| macromolecules | 3,160 | 4,350 | 4,423 |
| ligands | 55 | 3 | 143 |
| solvent | 648 | 370 | 714 |
| Protein residues | 375 | 554 | 555 |
| RMS(bonds) | 0.007 | 0.005 | 0.006 |
| RMS(angles) | 1.058 | 0.72 | 0.84 |
| Ramachandran favored (%) | 98.53 | 96 | 98 |
| Ramachandran allowed (%) | 1.47 | 3.9 | 2.3 |
| Ramachandran outliers (%) | 0 | 0.18 | 0 |
| Rotamer outliers (%) | 0.3 | 0.23 | 0.22 |
| Clashscore (MolProbity) | 1.14 | 2.16 | 1.27 |
| MolProbity Score (percentile) | 0.82 (99^th^) | 1.25 (99^th^) | 0.86 (100^th^) |
| Average B-factor | 13.33 | 26.47 | 16.43 |
| macromolecules | 10.12 | 25.95 | 14.15 |
| ligands | 31.37 | 26.81 | 21.15 |
| solvent | 27.26 | 32.64 | 29.57 |
| Number of TLS groups | 0 | 20 | 18 |
