# Supplementary material for: Computational design of environmental sensors for the potent opioid fentanyl
Source: eLife. 2017 Sep 19;6:e28909. doi: 10.7554/eLife.28909 (PMC5655540; doi:10.7554/eLife.28909)
Supplement: Peer review history

# Decision letter

**DOI:** 10.7554/eLife.28909.028

**Affiliations:** The Scripps Research InstituteUnited States

In the interests of transparency, eLife includes the editorial decision letter and accompanying author responses. A lightly edited version of the letter sent to the authors after peer review is shown, indicating the most substantive concerns; minor comments are not usually included.

Thank you for submitting your article "Computational Design of Environmental Sensors for the Potent Opioid Fentanyl" for consideration by *eLife*. Your article has been reviewed by two peer reviewers, and the evaluation has been overseen by a Reviewing Editor and Arup Chakraborty as the Senior Editor. The following individual involved in review of your submission has agreed to reveal his identity: Brian K. Shoichet (Reviewer #3).

The reviewers have discussed the reviews with one another and the Reviewing Editor has drafted this decision to help you prepare a revised submission.

Summary:

This is a very impressive paper on the joint computational and experimental design of proteins to bind fentanyl, a hydrophobic drug of much current interest. The binding designs can be used as an antidote or sensor component for the drug. The work is absolutely state-of-the-art for protein design. The paper is dense with report of the initial designs, their optimization, binding assays, crystal structures for apo and complexed proteins, and initial results for sensor systems in plants. The fact that they were able to redesign a sugar (polar molecule) binding protein to bind fentanyl is striking, as is the performance of Fen49.1. There are no significant issues with the manuscript.

Experiment for consideration during revision.

The observation of the bridging chloride ion must have come as a surprise, and its properties will differ greatly from the design bridging water. How important is that chloride? Without it, the fentanyl's cationic nitrogen – which for the Mu Opioid Receptor is certainly the key recognition feature – is not complemented by any single protein group. This not only distinguishes it from MOR recognition, but from almost all aminergic transmitters and drugs (some, it is true, are recognized by groups other than acids, but they are almost always recognized by *something*, such as Tryp/Tyr cages). Here, absent the chloride, that is not the case.

Have you looked at binding in the absence of chloride ion? It appears that all the assays were in PBS--how about using a simple KPi buffer? If there was no chloride, would fentany still bind?

# Reply: Author response

**DOI:** 10.7554/eLife.28909.029

*Experiment for consideration during revision.*

The observation of the bridging chloride ion must have come as a surprise, and its properties will differ greatly from the design bridging water. How important is that chloride? Without it, the fentanyl's cationic nitrogen – which for the Mu Opioid Receptor is certainly the key recognition feature – is not complemented by any single protein group. This not only distinguishes it from MOR recognition, but from almost all aminergic transmitters and drugs (some, it is true, are recognized by groups other than acids, but they are almost always recognized by *something*, such as Tryp/Tyr cages). Here, absent the chloride, that is not the case.

*Have you looked at binding in the absence of chloride ion? It appears that all the assays were in PBS--how about using a simple KPi buffer? If there was no chloride, would fentany still bind?*

Fen49 was designed to make indirect protein contacts with the fentanyl tertiary amine through a bridging water. The chloride observed in the structure very closely mimics this water; it is precisely positioned at the design location, and satisfies the design interaction of the fentanyl tertiary amine with Y80. We agree with the reviewers that the presence of a chloride in the structure was a surprise. The chloride appears to be dispensable for binding, however, as fentanyl is still able to bind to Fen49 in a buffer system without chloride, albeit with slightly reduced affinity. Chloride would be better suited than water to complement the positive charge of the tertiary amine. However, in the absence of chloride, water appears to be sufficient for binding in a manner that is dependent primarily on apolar interactions and shape complementarity.
